# WatCon: A Python Tool for Analysis of Conserved Water Networks Across Protein Families

**DOI:** 10.1021/jacsau.5c00447

**Published:** 2025-11-20

**Authors:** Alfie-Louise R. Brownless, Travis Harrison-Rawn, Shina C. L. Kamerlin

**Affiliations:** † School of Chemistry and Biochemistry, 1372 Georgia Institute of Technology, 901 Atlantic Drive NW, Atlanta, Georgia 30332-0400, United States; ‡ School of Chemical and Biomolecular Engineering, Georgia Institute of Technology, 311 Ferst Drive, Atlanta, Georgia 30332-0400, United States; § Department of Chemistry, Lund University, Box 124, 221 00 Lund, Sweden

**Keywords:** Water Networks, Graph Theory, Protein Tyrosine Phosphatases, Protein Structure, Water Conservation

## Abstract

Water structure is crucially important to protein function and catalysis and can be conserved throughout related proteins despite differences in sequence. The complex hydrogen-bonding networks formed by water molecules and protein residues have been studied extensively, and graph-theory-based methods have frequently been used to describe these networks. Although there exist a number of tools which can be used to track water positions and networks, corresponding methods for easily analyzing complex water network structure across related proteins are limited. To address this challenge, we present here a new tool, WatCon, an open-source Python package which can be used to analyze water positions and water network structure across protein families using both dynamic and static structural information. Importantly, WatCon can be used to classify conservation of water networks, characterize water networks across structures, and project subsequent results for easy visual interpretation. To illustrate WatCon usage, we provide five example applications illustrating WatCon analyses of static structures, dynamic trajectories, and cross-family analysis. This in turn showcases the utility of WatCon for enhancing our understanding of biochemical systems, predicting water hotspots of potential relevance to protein engineering and predicting pathogenic mutations. WatCon can be downloaded at https://github.com/kamerlinlab/WatCon and is available under the GNU General Public License v3.0.

## Graphical abstract

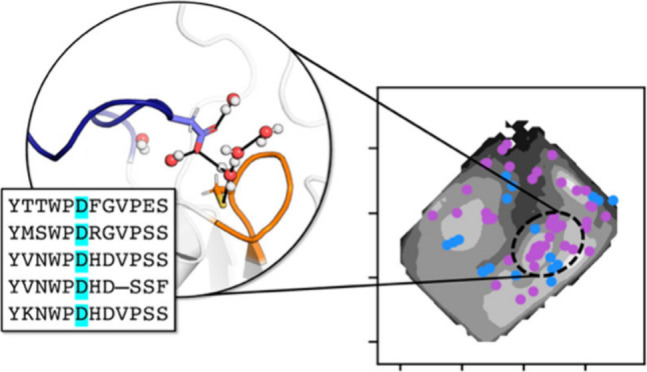

## Introduction

Biological macromolecules such as proteins and nucleic acids are well-known to be highly dependent on the structure and dynamics of internal and external solvent, especially water.^1^ In particular, water molecules play an important role in correct protein folding, stability, and function, as detailed in refs 2–24 (among many others). Because of the functional importance of water to proteins, tracking the existence and conservation of water networks is not just important for understanding protein evolution, but also plays an important role in protein engineering and design,^25^ and in drug discovery.^23,26–33^

Due to its strong hydrogen-bonding affinity, water molecules can form complex interaction networks across and within protein scaffolds, contributing to the stability of these macromolecular structures.^10^ Furthermore, water network structures have been observed to change dependent on enzyme conformation, whether or not ligand is bound, and other external factors such as temperature.^34,35^ It is in this regard that water can notably play both an enthalpic and entropic role in ligand binding, and thus can contribute toward catalytic prowess of a given enzyme.^23,34^ It has recently been shown that a single point mutation can dramatically effect enzymatic catalytic efficiency via disruption of highly ordered water network structures.^34^ Given the importance of water molecules to protein structure and function, there exist a number of tools for calculating water density hotspots and tracking protein–water interactions across molecular dynamics trajectories.^27,33,36–41^ However, there are negligible tools available to aid in water network analysis across crystal structures,^36,41^ let alone across dynamic trajectories of ensembles of multiple proteins (for instance across a protein family or superfamily). Such a tool would provide important insight into how variations in protein sequence can affect enzyme catalysis and protein structure/function, via alterations in evolutionarily conserved water–protein interactions.

There has been great interest in applying network models and graph theory in biological sciences, to understand a wide range of biological problems.^42^ More specifically, graph theory has been successfully exploited both to understand protein structure, function and evolution,^42–45^ and as a tool for characterizing water networks in proteins.^23,33,41,46–48^ We recently developed a Python-based tool, Key Interaction Networks (KIN),^49^ which uses residue interaction networks (RINs) to characterize evolutionarily conserved noncovalent interaction networks in proteins. KIN focuses on interactions within proteins and across protein subunits; however, it has been demonstrated that inclusion of water molecules in RINs improve the description of the protein interaction network, highlighting the importance of water to protein function.^50^ Here, we build on KIN to present WatCon (Figure 1), a multipurpose water network analysis tool and water tracker. We outline the utilization of WatCon to find conserved water-network features across protein families and to use as a water-tracker for analysis of molecular dynamics trajectories. WatCon is an open-source Python package available on https://github.com/kamerlinlab/WatCon, under the GNU General Public License v3.0 (GPLv3).

**Figure 1:**
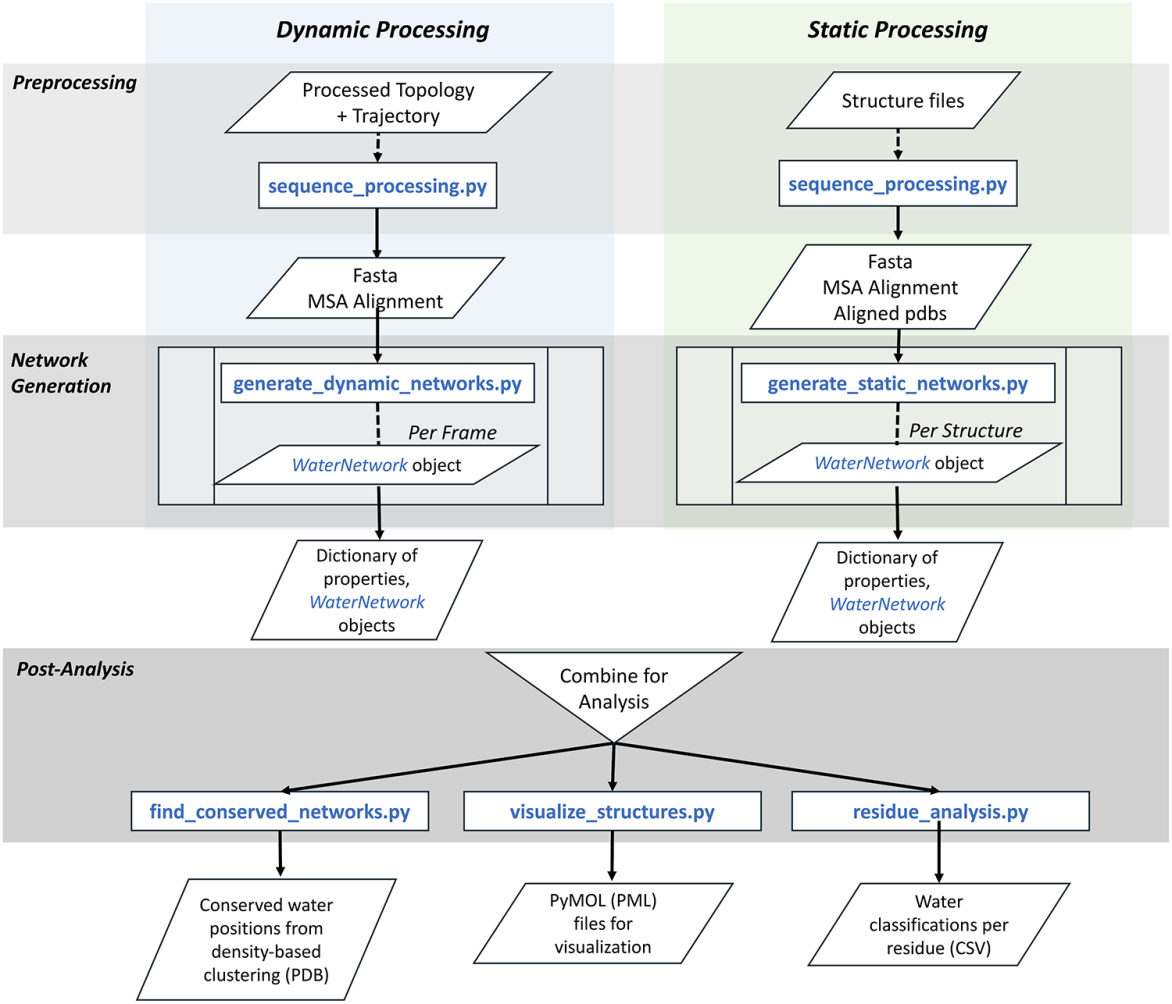
Schematic overview of the WatCon Python package. Note that analysis can be performed using either static structures (from e.g. X-ray crystallography), or simulation trajectories, or a mix of the two.

## Experimental Design

Below we describe the layout of WatCon along with a standard workflow (Figure 1) that can be used as a starting point for conserved water network analysis.

WatCon has been designed to provide the ability to track conserved water network models across a series of static structures (from e.g. X-ray crystallography), a series of molecular dynamics trajectories, or a mix of the two, based on analyzing either water networks present in experimentally resolved structures, or water networks present in molecular simulations after solvation and equilibration of experimental or predicted structures. WatCon is, therefore, divided into two main sections: a static structure processing pipeline and a dynamic trajectory processing pipeline. Regardless of the path the user takes, our analyses revolve around defining a graph where the locations of water molecules are nodes and any connections among them are edges. Further, we provide the user the option to focus on only water–water interactions, only water–protein interactions, and/or a subset of each (i.e., focusing on a given region of interest instead of their entire system). WatCon, therefore, has been constructed as a flexible tool capable of addressing a variety of problems.

### Dependencies

WatCon requires a number of dependencies. We list here both the dependencies and the version of each package utilized for these tests. WatCon was written and tested in Python3.9,^51^ with the following dependencies: (1) MDAnalysis 2.2.0;^52,53^ (2) Modeller 10.5;^54^ (3) NetworkX 3.1;^55^ (4) NumPy 1.24.3;^56^ (5) Pandas 2.0.2;^57^ (6) Scikit-learn 1.4.2;^58^ (7) SciPy 1.10.1;^59^ (8) joblib 1.3.2. W examples are provided both as part of the WatCon documentation at https://watcon.readthedocs.io and on GitHub at https://github.com/kamerlinlab/WatCon.

### Preparing Structures for WatCon Analysis

In order to generate water networks that can be compared across both individual selections of proteins and whole protein families, both physical three-dimensional structural alignments and sequence alignments need to be conducted. First, spatial alignment is crucial for our tool to function properly, as appropriate alignment allows us to cluster shared water molecules by physical location, and to identify conserved water molecules across independent protein structures. Second, it is equally important to apply a sequence alignment to determine a common residue indexing scheme across independent structures, to make it easier to ensure that conserved water molecules are correctly identified, despite sequence differences among related proteins. Specifically, this information can help identify water–protein interactions (and water molecules) that persist throughout protein families and identify individual family members where water–protein interactions conserved across the broader protein family are lost.

Since a major component of WatCon analysis involves three-dimensional density-based clustering (which WatCon can perform using either DBSCAN,^60^ hdbscan^61^ and/or OPTICS,^62^ as implemented in Scikit-learn^58^), WatCon has specific structure processing requirements for input structures. In the case of Protein Data Bank^63^ (PDB) input file format, we require all structures to be aligned to a common reference, which can be done easily using WatCon. Additionally, WatCon requires that all simulation trajectories have been postprocessed, accounting for any periodic boundary condition (PBC) wrapping that may be required. WatCon leaves postprocessing trajectories up to user preference. We note that WatCon is able to read simulation trajectories in all formats that can be handled by MDAnalysis,^52,53^ which in turn is able to read all common simulation formats.

Following initial structure processing, WatCon interfaces with the Modeller package^54^ in order to perform an initial multiple sequence alignment (MSA). The alignment follows the standard procedure as described in the Modeller manual, using the residue type as the alignment feature, and with a maximum gap length of 20. The subsequent percentage identity matrix is obtained using Clustal Omega.^64^ We then use Modeller in order to perform a structural alignment, aligning everything onto one structure within an identified directory of structures. Alignment is a mandatory step of the WatCon analysis process, but if desired, the user can instead perform sequence and structural alignments separately using their preferred software and omit the use of our sequence alignment implementation for this step. For instance, WatCon can also use an existing MSA in Protein Information Resource (PIR) format.

### Water Network Generation

WatCon is able to construct both water–water and/or protein–water networks utilizing either experimental or predicted protein structural data from X-ray crystallography, NMR spectroscopy, cryo-electron microscopy, or protein structure predictions (using e.g. AlphaFold^65^ and other prediction tools after solvation using appropriate water prediction tools), or dynamical trajectories from biomolecular simulations. We note that in the present study, we have retained all crystallographic waters in our molecular dynamics simulations; however, WatCon is designed to follow user-preference, and the user can either retain crystallographic water molecules or remove them and resolvate prior to setting up MD simulations for dynamic trajectory analysis (see also further discussion in “WatCon Pitfalls and Caveats” in the Supporting Information).

When constructing the water network, by default, WatCon uses a 3.8 Å (user-adjustable) distance cutoff on the donor–acceptor distance, and, in the case of structures where protons are present (e.g., from simulation trajectories or neutron structures) an additional (user-adjustable) 150° donor–hydrogen–acceptor angle cutoff to define hydrogen bonds. These are slightly higher than the values presented in ref 66, as having a slightly looser definition of hydrogen bonding criteria as a default allows WatCon to identify prospective hydrogen bonds in lower-resolution structures, as discussed in “H-bond Definitions” in the Supporting Information. We note importantly that the H-bond tolerances can be modified by the user and increased or lowered as needed for a specific system.

Once potential water–water and water–protein interactions are determined using MDAnalysis^52,53^ to parse structure files, WatCon uses the NetworkX^55^ Python library to create a graph which contains all of the coordinates and connections involved in the water network, which is then utilized for further analysis. Depending on the computational resources available to the user, WatCon provides the option of generating either an undirected network involving only oxygen atoms of the water molecules, or a high resolution directed network also involving the hydrogen atoms (where their positions are known). Further, WatCon can generate either water–water, water–protein, or full (both) networks, and, if necessary to save computational cost, WatCon further allows for a flexible “active-region” to be defined as a user-tunable sized sphere centered on atom(s) of interest (or the center of mass of atoms of interest), rather than calculating a full network for the whole system.

Networks are generated using a k-d tree algorithm in order to efficiently find the distances between the closest members of each water coordinate based on a user-defined cutoff (other approaches such as Bridge^67^ have implemented a similar idea). We suggest using a default of 10 closest members as we see comfortable convergence in the number of calculated water–water interactions and overall graph density values at this cutoff (Figure S1), but this value may be adjusted for the needs of a specific system, both through increasing the cutoff to capture more interactions, and through decreasing the cutoff to save computational time. These connections are then added as edges into the network object if each distance is below a certain user-tunable distance threshold. Once the full network is created, a number of useful metrics are returned and described in Example Applications. Additionally, WatCon can be used to generate PyMOL^68^ projection .pml files that can be used to visualize the water networks of interest at each trajectory frame or PDB. Finally, network processing using WatCon has been parallelized using the Joblib Python package (see https://github.com/joblib).

### Water Network Analysis

Since we construct WaterNetwork objects in order to generate NetworkX^55^ graph structures (Figure 1), the entire NetworkX^55^ library can be utilized for analysis of water network structures. However, we include in WatCon certain key metrics which we believe are valuable for analyzing complex water networks in proteins. Specifically, we can calculate the density of a network as the ratio of its edges and number of possible edges.^69^ Graph density can be very useful to calculate as the density of waters within a particular region can carry information regarding protein conformation. Additionally, static (experimental) structures can have drastically different numbers of resolved waters depending on the resolution of the structure, which is important to consider when conducting an extensive analysis on water networks.

WatCon provides a number of additional analysis options to the user, specifically WatCon gives the users the ability to: (1) Obtain the number of connected components for each network using NetworkX^55^ directly, which can provide valuable information regarding how lengths of water chains change between structures. (2) Retrieve the characteristic path length, or average path length, of a given network. The characteristic path length is defined as the shortest number of edges between two connected vertices, averaged over all vertices and can be used to indicate the “tightness” of a graph.^70^ Since the calculated WaterNetwork objects typically do not correspond to fully connected graphs, we average the NetworkX^55^ calculated characteristic path length over all connected components within a given water network. (3) Calculate the characteristic path length for the longest connected component of a graph. (4) Compute the clustering coefficient for each node (atom) within the graph using built-in NetworkX functionality.^55^ (5) Calculate the shortest path connecting two atoms of interest using NetworkX functionality. (6) Calculate the density of an entire graph (defined as the ratio of edges and possible edges within a graph).^71^ (7) Calculate the entropy of a graph by determining how ordered the graph is based on the distribution of node degrees; a more ordered graph will have nodes with similar numbers of corresponding edges, while a less ordered graph will be more variable.

### Tracking Conserved Water Molecules and Networks within Protein Families

A major component of the WatCon package is that it can track individual water molecules and compare protein water networks within a larger protein family. In this way, WatCon becomes useful as a way to combine enormous collections of three-dimensional data and extrapolate subtle yet important information in an automated manner. In addition, WatCon is able to create conserved networks across trajectories and static protein structures utilizing combined water positions and via analysis of conserved water–protein interactions across sequences.

In the context of this application, WatCon provides the ability to identify conserved water positions and compare with conserved sequence structures within a protein family, akin to analyses performed by our recent tool Key Interaction Networks (KIN),^49^ which uses network modeling to characterize conserved noncovalent interaction networks in protein families. In brief, WatCon first creates or utilizes an existing multiple sequence alignment for all proteins within a family of interest (to ensure a common indexing scheme for the family), and then compares all protein–water interactions across each scaffold to determine whether individual water locations are conserved and how those conserved water positions align with conserved sequences.

Often, it is useful to characterize residues based upon the number of water–protein interactions that can form across either a series of independent structures or a series of trajectory frames. To address this, WatCon provides an interaction score to classify water–protein interactions. This is calculated as the total number of water–protein interactions divided by either the total number of structures (in the case of static structure analysis) or by the total number of frames (in the case of dynamic trajectory analysis). This for example, provides a metric whereby if a residue interacts with a water molecule 100% of the time or with two water molecules 50% of the time, then that residue would have an interaction score of 1; we therefore recommend presenting this number along with the average number of simultaneously interacting molecules to get a holistic view on per-residue water interactions.

Importantly, we can characterize water positions via the measurement of two angles (Figure 2); in this way we can effectively distinguish between distinctly different water positions using only two dimensions, while also taking into account any differences in sequence structure (see “Differentiating Between Water Molecules Using WatCon” in the Supporting Information). The distribution of these two angles can then be projected onto a 2-D plot, allowing for easy detection of any notable conservation of water positions, as well as any outliers in this conservation. Importantly, this allows for highly conserved structural water positions to be identified and easily compared to differences in sequence composition across similar proteins within a given family.

**Figure 2:**
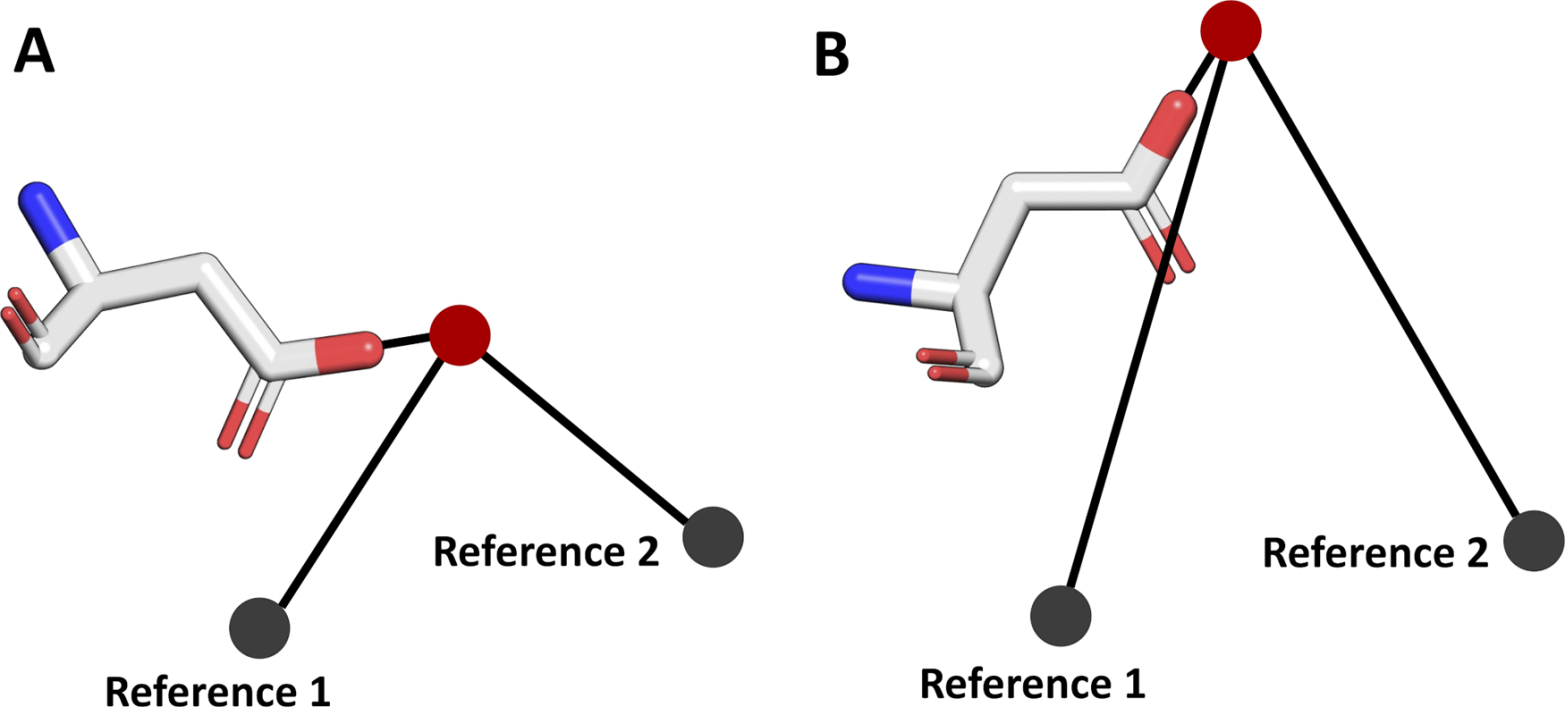
Visualization of how WatCon differentiates individual water–protein interactions. Two reference points are defined, and the interacting protein atom – water oxygen – reference point angles are measured. The Reference 1 and Reference 2 coordinates are set as using (0, 10, 0) and (10, 0, 10) Å by default (assuming protein structures are aligned to the same coordinate axis), but can also be adjusted to any relevant C_α_ atom or other coordinate description. In doing so, WatCon can effectively distinguish between (for instance) the interacting water molecules between conformations A and B of the same residue, while also taking into account conserved sequence information. This is useful for analyzing cases where an interacting residue varies within a given family but the interacting water position remains constant.

Following from this, WatCon is also able to generate a conserved water network via clustering of available water positions. This allows for water hotspots to be easily identified, agnostic of individual water–protein interactions. In other words, WatCon provides the ability to compare water positions to a local “average”, which can pick up on highly conserved water–water interactions along with isolated hydrophobic pockets, among other properties. After generating networks for each crystal structure (or trajectory frame), WatCon creates a combined network with all water positions. WatCon then applies a density-based clustering method (the user can choose between hdbscan,^61^ OPTICS,^62^ and DBSCAN^60^), and creates a summary water network using the centroids of each cluster. In this regard, WatCon defines the conserved water network and then provides a conservation “score” for each protein in sequence. This score is calculated using eq 1:

1 $$\frac{1}{N_{s}}\underset{i}{\sum }\frac{a_{i}}{1+\underset{j}{\sum }w{\left(a_{i}\right)}_{j}}$$

where *a*_*i*_ indicates the number of conserved waters which are matched within the given PDB structure and *w*(*a*_*i*_)_*j*_ indicates the number of total waters within a user-definable sphere (default 6 Å) around the indicated water, *a*_*i*_. The resulting value is then normalized by the factor 1/*N*_s_ where *N*_s_ is the total number of summary waters.

Generally, the probability of finding a “match” between a conserved water and a water present within a PDB structure is proportional to the local density of waters which surround a given point of interest. As a result, we effectively normalize our conservation score via the use of local density pockets around our areas of interest, and we confirm that this conservation score is not correlated with the density of any given water-network structure (Figure S2). Note that we normalize by our sum of local waters plus 1 to include the original conserved water molecule, in the case that there are no additional local waters within the neighboring sphere. Therefore, under the “perfect” case where all waters are perfectly matched, the conservation score would be 1 (an example of this is provided in Application 4).

Additionally, we can calculate a conservation score for each water cluster by summing the instances that there exists a water within a user-adjustable (default 1 Å) distance of each cluster. Similarly, we further define a pairwise conservation score in the same manner by tracking, for each pair of water clusters within a user-defined cutoff distance (default 2 Å), how often there exist waters within a user-adjustable (default 1 Å) distance of both clusters within the pair.

By calculating a conservation score for each protein within a group, it is immediately easy to identify which structures have water–water and water–protein interactions which are closest to the “average” and those which have more unique structures. These structures can then be investigated more closely, if desired. By developing WatCon, we aimed to increase the throughput and ease of comprehensive water network analysis across both static and dynamic structures. Our conservation metrics were therefore designed to aid determination of protein structural regions which showcase notable changes in water network structures. To showcase these ideas, we present the use of our tool in five example applications below. In the case of analysis of dynamical trajectories, computational details of how the simulations underlying these applications were performed are provided in the Supporting Information.

## Example Applications

### Example Application 1: WatCon as a Water Tracking Tool

As a simple starting point, we present an example of the application of WatCon in tracking water–water and water–protein interactions across molecular dynamics trajectories. As with protein structures, water networks are also dynamic, and thus it is necessary to be able to track fluctuations in water networks over time (for instance, by analyzing molecular simulation trajectories). This is particularly important, given the importance of protein–water interaction networks to maintaining and regulating protein dynamics.^1,33,72–77^ In this context, WatCon can be used as a water tracker tool to observe dynamic changes in water networks as a function of time. We note that there already exist a number of tools that can track water molecules in MD simulations (e.g., refs 30, 33, 38, 41, 52, 78, and 79, among others), and thus, this application is presented mainly to showcase WatCon capabilities.

In this section, we provide an example of using WatCon to glean insight in how the structure of an enzyme active site can change dependent on both time and protein sequence, via changes in internal water networks. Being able to characterize water networks can be particularly useful for protein engineering applications, where changes in solvation patterns can impact activity and selectivity,^1,10,80^ as well as providing information about how water–protein interactions fluctuate over time. This functionality can be especially crucial when a complex network of water molecules is found within a particular active site, or when water molecules are known to play a catalytic role in an enzymatic reaction. Additionally, in enzymes that are regulated by catalytic loop motion, the motion of solvent molecules into and out of the active site plays a fundamental role in catalysis,^81^ and WatCon allows for easy tracking of the mobile water molecules.

We provide an example of this functionality of WatCon using the protein tyrosine phosphatase (PTP) PTP1B as a model system. This enzyme contains a key catalytic residue, D181, which facilitates acid–base catalysis during the PTP-catalyzed reaction (Figure S3). D181 is located on a mobile catalytic loop, the WPD-loop (Figure S3).^82^ The p*K*_a_ of the D181 side chain in PTP1B is elevated, due to both the local protein and solvent environments in the PTP1B active site, allowing it to act as a general acid.^83^ Further, the nucleophile in the rate-limiting hydrolysis step^84,85^ of the two-step reaction catalyzed by PTP1B (and other PTPs) is an active site water molecule (Figure S3), and once again, correct water positioning becomes important to catalysis. Thus, tracking water networks in PTP1B (and other enzymes regulated by conformational changes) can potentially provide insight into the catalytic function of these enzymes.

Considering that the time-dependent movement of the mobile WPD-loop is intimately connected with PTP1B-mediated catalysis, we first demonstrate the utilization of WatCon as a trajectory water tracker to understand how solvation effects are modulated by the movement of this key mobile loop. Here, we collected 8 replicas of 1.5 μs trajectories initiated from each of the unliganded closed WPD-loop and open WPD-loop conformations (24 μs simulation time in total) to form our starting data sets. Using the generate_dynamic_networks module of WatCon, we created a series of WaterNetwork objects to describe each frame of our simulations. A region of interest can be defined as a sphere around a user-inputted center: here, we roughly define the PTP1B active site by a sphere of 9 Å radius around the center of mass between the catalytic cysteine C215 residue and the R221 residue, located on helix α4, which connects to the phosphate binding P-loop and influences catalytic activity.^86^

Using WatCon, we calculated the number of water–water only and water–protein only interactions to compare with the distance between the center of mass of the P-loop and WPD-loop (Figure 3). In doing so, we can see that as the distance between the P-loop and WPD-loop increases, the number of water–water interactions also tends to increase (Figure 3). In contrast, the number of water–protein interactions tends to subtly decrease as this transition occurs. Furthermore, we can use WatCon to calculate graph density and entropy (described in ref 87) under these same conditions. We observe that both graph density and entropy can tend to increase with WPD-loop opening, albeit subtly (Figure S4). This is a logical trend as the increased solvation due to the more open WPD-loop allows for more water–water interactions to be present. This results in a larger graph density, as there are more possible connections that can be made due to flexibility in movement of water molecules compared to protein atoms. Furthermore, graph entropy increases as there are more combinations of water–water interactions that can form as local water concentration increases. In this way, graph entropy is a useful metric for determining how “ordered” a water network is. Using WatCon, we observe that WPD-loop closure not only sequesters the active site from solvent, but also enforces a more ordered water network across the remaining active site water molecules. More generally WatCon can be used to easily observe how water network structure changes due to modulations in structure, information which can guide protein engineering practices via directed modulation of structured water networks.

**Figure 3:**
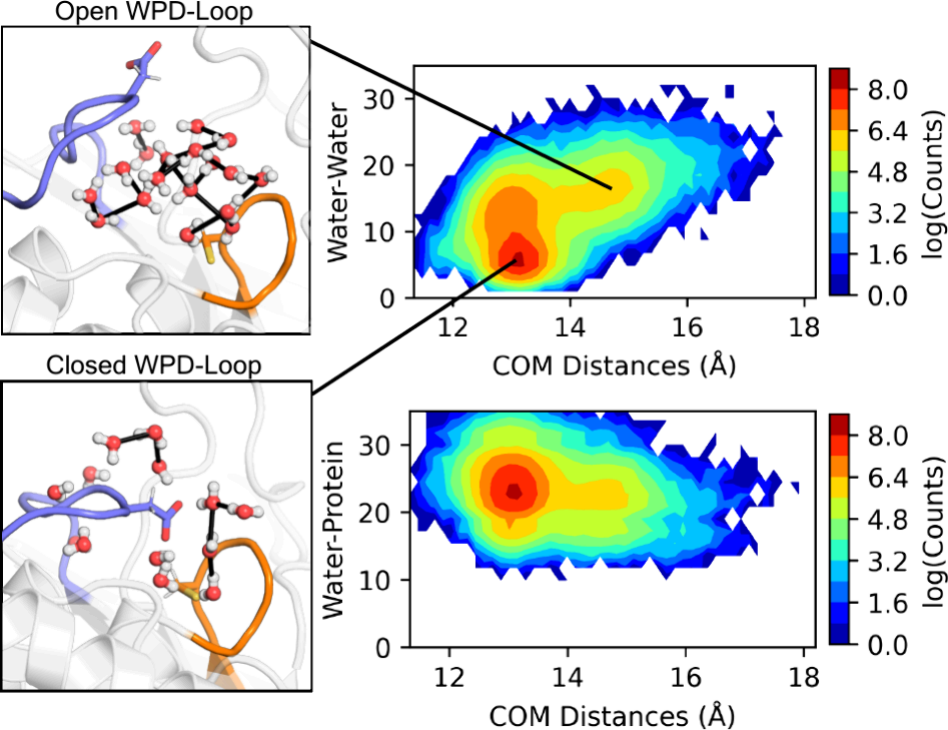
Comparison of center of mass (COM) distances (Å) between the P-loop and WPD-loop of PTP1B and the calculated number of water–water and water–all (combined water–water and water–protein, denoted Water–Protein) interactions across 8 × 1.5 μs simulation replicas of PTP1B, initiated from each of the WPD-loop closed and open positions (16 trajectories in total). Shown here also are representative structures of the active site water–protein interaction networks in each of the calculated minima on the water–water histogram.

Since WatCon can act as a water tracker, we demonstrate its utility for determining prolonged water–protein interactions across a simulation trajectory. WatCon makes it simple to determine which residues interact with the most waters simultaneously, playing major roles in coordinating complex water networks, and which water–protein interactions persist for the longest amount of simulation time. Both of these concepts allow us to predict the outcome of residue mutations on complex water network structure formation. To demonstrate, we determined which residues interact with the most waters simultaneously (Figure 4).

**Figure 4:**
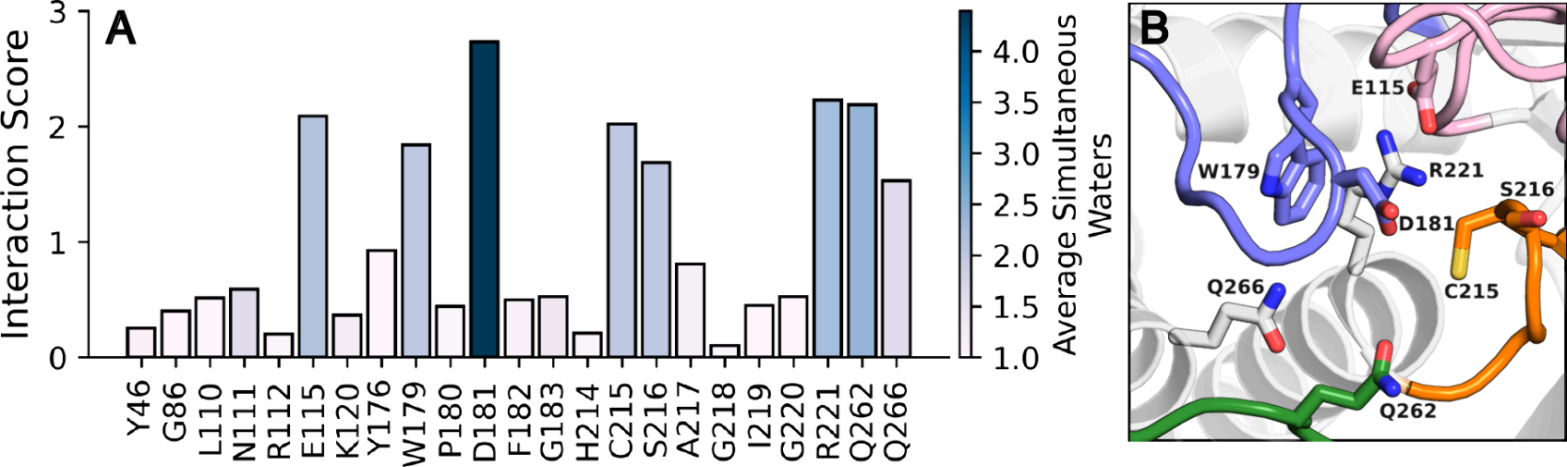
(A) Number of water interactions per residue during 8 × 1.5 μs simulations of PTP1B from each of the WPD-loop closed and open conformations (16 trajectories total), normalized by the total number of trajectory frames with key active site residues for PTP1B simulations. Bars are colored by the average number of (nonzero) simultaneous water molecules each residue forms interactions with, to discriminate between, for instance, transient interactions with multiple water molecules simultaneously, vs sustained interactions with a single water molecule. (B) Visualization of residues with a normalized water interaction score > 1. Of the key catalytic loops in PTPs, the WPD-loop is colored blue, the P-loop is colored orange, the E-loop is colored pink, and the Q-loop is colored green.

Based on this analysis (Figure 4), we notice that the side chains of residues E115, W179, D181, C215, S216, R221, Q262, and Q266 (and the backbones of W179, D181, S216 and R221) interact with far more water molecules simultaneously than the other active site residues. As a result, targeted modification at these residue sites would be expected to cause dramatic shifts in water network structure and thus potentially dramatic shifts in enzymatic efficiency (which may, or may not, be disruptive). In fact, previous studies have indicated that these residues are crucial in regulating active site dynamics. In particular, other than the residues D181 and C215 which play active roles in the catalytic mechanism, Q262 serves to strategically position a nucleophilic water molecule,^88^ S216 plays a major role in stabilizing substrate position,^89^ E115 and R221 stabilize active site hydrogen-bonding networks,^90^ and Q266 has been proposed to play a major role in catalytic residue coordination.^91^ We also note that both S216 and Q266 coordinate water molecules, and these coordination patterns can be modified by allosteric changes.^90^ We further point out that nonpolar residues, such as L110, can exhibit notable interaction scores, due to interactions occurring among backbone atoms (Figure S5), which WatCon can easily differentiate from side-chain interactions. Since WatCon can be used to identify the residues that are most important in water network coordination, WatCon-based analysis can in turn be used to supplement protein engineering studies and predict which amino acid substitutions can affect enzyme efficiency via modification of water-network structure.

### Example Application 2: Comparison of Static and Dynamic Water Networks

One strength of WatCon is that while it can generate water networks based on either static structural information or dynamical trajectories, it can also combine the two. This means, for instance, if one is interested in analyzing water networks across an entire protein family, one does not necessarily need to simulate every single member of the family to obtain dynamical trajectories, but rather can analyze static water networks across the full family, and only simulate key proteins in the family to obtain more detailed dynamical information.

Before we provide an example of combining static and dynamic information, we first compare network properties obtained between static and dynamic structures separately. We will compare water networks calculated from all available X-ray crystallographic structures of PTP1B with a closed WPD-loop conformation that are available in the Protein Data Bank^63^ (PDB) (Table S1), to results calculated from 8 replicas of 1.5 μs simulation data initiated from the WPD-loop closed structure of PTP1B. In doing so, we comment both on the expected distributions of our calculated metrics from an ensemble of static structures and how those differ from simulation data, noting particularly the differences in conclusions that one might draw between analysis of either static experimental structures or molecular dynamics trajectories alone (rather than combining the two). We then use this information to justify the aggregation of both simulation trajectories and static structural data to obtain a better picture of water network structure and dynamics.

We note that our model enzyme, PTP1B, is particularly well placed to be used as a model system to showcase this application, due to the rich structural data available in the PDB (∼400 structures of wild-type and mutant forms of this enzyme), which allows for extensive analysis. For the purposes of this application example, we removed mutant structures as well as structures lacking resolved water molecules, as described in “WatCon Analysis” in the Supporting Information, leaving us with 306 experimental structures of PTP1B, of which 82 structures have closed WPD-loops and were used for analysis (Table S1). As introduced in Application 1, we further generated new molecular dynamics trajectories, following protocols utilized in our prior work,^92^ as a point of reference for comparison to static structure analysis presented in this Application. Such comparative analysis is important, given that prior work has also indicated that there can be significant differences between the water networks available in crystal structures and those calculated from molecular dynamics trajectories.^93^

To compare water network properties between the static and dynamic water networks, we utilized 8 × 1.5 μs trajectories run from unliganded PTP1B in the WPD-loop closed conformation. We note that, despite the lack of a ligand in the active site, the WPD-loop remained closed 98% of simulation time. For better direct comparison, only frames with <14 Å distance between the centers of mass of the WPD-loop and P-loop were used for simulation analysis (average distance in the closed crystal structures ∼ 13.1 ± 0.3 Å). As before, we define the active site of PTP1B as a 9 Å sphere centered on the center of mass of residues R221 and C215.

We first point out that, perhaps surprisingly, despite the difference in distributions, the average density of the graphs is not dramatically greater for MD trajectories versus static structures (Figure 5A). This is a result of how the density is calculated, as a ratio of actual connections to possible connections; since we utilize a generous hydrogen-bonding cutoff for our crystal structures (3.7 Å O–O) distance, we observe a high percentage of connections compared to the relatively low number of water molecules that are resolved. This occurs simply due to the fact that it is very challenging to resolve water molecules in crystal structures if they are not tightly bound to the protein, and thus the crystal structures do not provide the full water network, but the water positions that are resolved are bound very tightly to the protein and thus are consistently likely to form interactions.^94^ Related to this, when analyzing the simulation trajectories, we find a subtly higher graph entropy for the dynamic networks compared to the static networks (Figure 5B), which occurs because the larger number of interacting waters results in a less-predictable network structure, and thus a higher entropy. Further, the average characteristic path lengths (CPL) are also very similar irrespective of the nature of the data used (Figure 5C). Finally, we observe more water–water and water–protein interactions compared to static structures, due to the increased number of water molecules present (Figures 5D,E).

**Figure 5:**
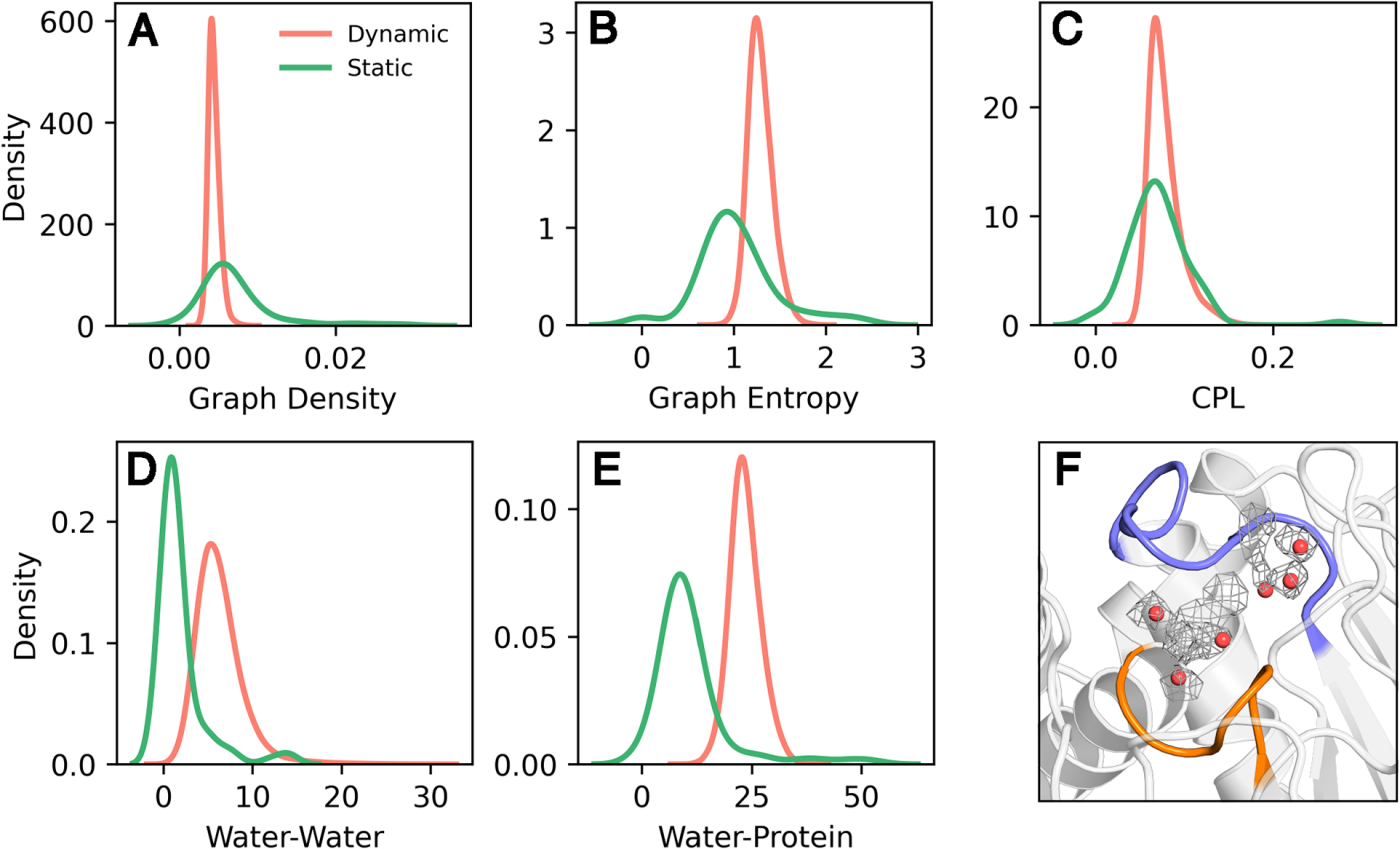
(A–E) Kernel density estimate (KDE) plots of calculated properties, showing a comparison between data obtained from analysis of dynamic trajectories (8 replicas × 1.5 μs simulations of PTP1B initiated from the WPD-loop closed conformation) and of static structures. Shown here are (A) the graph density, (B) graph entropy, (C) the average characteristic path length (CPL) (D) the number of water–water interactions (Water–Water), and (E) the number of water-all interactions (combined water–protein and water–water interactions, denoted Water–Protein). (F) Comparison of highest water occupancy regions obtained from analysis of molecular dynamics trajectories of PTP1B vs clustering on available crystal structures, with the WPD-loop in its catalytically closed conformation. Densities are shown in wire mesh representation, presented at an isovalue of 1.0, calculated from a 9 Å sphere and centered on the center of mass of the R221 and C215 residues, based on 8 × 1.5 μs molecular dynamics simulations of PTP1B. Static structure clusters were calculated using hdbscan clustering^61^ based on 82 closed WPD-loop structures of PTP1B (Table S1).

For further comparison of water networks in our simulated trajectories versus the experimental structures, we have explored the position of water hotspots provided by WatCon (see technical considerations in “Supplementary Technical Considerations” in the Supporting Information). Based on this analysis, we demonstrate that the MD simulations are able to identify many of the highly conserved water positions identified from crystal structure analysis, with all six of the six identified clusters from the MD simulations being located very near or directly within a density hotspot. In all cases, there are water hotspots present in positions which mimic the phosphoenzyme intermediate (Figure S3), which are known to be highly conserved across all PTPs.^95^ Further, the MD simulations also identify additional water hotspots, which is to be expected as the experimentally determined positions are a subset of the full water network present in the protein. This highlights the value of combining analysis of static structures with data from molecular simulations, when sufficient computational resources are available to perform them.

We then classified our conserved water molecules based on angle calculations (as described in Experimental Design), creating a smooth distribution of angles from trajectory data and then overlaying with values calculated from crystal structures (Figure S6). In our analysis, we focused on three key catalytic residues: E120, D181, and Q262, due to their functional importance to the PTP reaction.^88^ We note that all three residues are located on mobile loops decorating the active site (Figure 4B). Our analysis demonstrates that while static structures show high conservation of water–protein interactions involving each of these residues, the MD trajectories sample a range of water positions involving either residue, a subset of which align well with the corresponding water positions sampled by the static structures, plus additional interactions not observed in the static structures. The exception to this is residue D181, located on the PTP WPD-loop, which shows a broad spread of hotspots from both the static structures and the simulation trajectories, and richer experimental hotspot information than in the case of the other two side chains. This again showcases the benefits of supplementing experimental with simulation data and performing combined analysis of the two data sets, which WatCon can facilitate.

### Example Application 3: Comparison of Water–Protein Networks Across Protein Families

An important core application of WatCon (and the primary reason we designed this tool) is the ability to evaluate conserved water networks across protein families. To illustrate this, we use the broader PTP family as an example. This protein family is very diverse while still maintaining a high degree of structural similarity in the catalytic domain, and a high degree of sequence similarity within the active site, including the conserved mobile WPD-loop, and the conformationally rigid phosphate binding P-loop with sequence ((H/V)­CX_5_R­(S/T)).^82,96,97^ The catalytic mechanisms of PTPs, how they are regulated by loop motion, and structure–function-dynamics relationships more broadly have been studied extensively elsewhere, and in particular an active site water molecule plays an important role as nucleophile (Figure S3).^68,72–93^ In this section, we showcase how WatCon can be used to track conserved waters across a family, and especially how the active site water networks in related PTPs differ from those of the WPD-loop-closed form of PTP1B.

We note that, as showcased in Application 2, analysis of water networks can be performed using either simulation trajectories, experimentally determined structures, or a combination of both, and this applies also to analysis across protein families. For the sake of this Application, we desired a rigorous comparison between water networks in PTP structures with open and closed WPD-loops, over evolutionarily related PTPs. Here, for simplicity, we focus only on experimentally determined structures. As a model family, we have chosen nonreceptor PTPs,^98,99^ because of the wealth of structural information available in the Protein Data Bank^63^ of PTPs from this family, with both closed and open WPD-loops. Specifically, structural data currently exists for the structures corresponding to the genes PTPN1, PTPN2, PTPN3, PTPN4, PTPN5, PTPN6, PTPN7, PTPN9, PTPN11, PTPN12, PTPN13, PTPN14, PTPN18, PTPN21, and PTPN22. Thus, we have analyzed all available structures of these PTPs present in the Protein Data Bank^63^ (Table S2). We note that, as discussed in “WatCon Pitfalls and Caveats” in the Supporting Information, resolution is an important factor to consider when selecting experimental structures for static analysis, as lower resolution structures will contain less reliable information about water positioning (and miss more water molecules). However, we also note that when working across a protein family, there can be variation in the quality of the experimental data available, and this can be a limiting factor when examining static structures without additional simulations.

We first begin by generating WaterNetwork objects conserved across all available experimental structures, once again defining a 9 Å sphere centered about the center of mass of R221 and C215 (PTP1B numbering) as our active region. Structures were selected as described in “Supplementary Technical Considerations” in the Supporting Information. In order to compare the structure of water networks for all of the given nonreceptor PTPs to the PTP1B WPD-loop-closed structure as a reference, we took the combined clustered water hotspots from the closed PTP1B crystal structures (Application 2) and calculated the conservation score for each PTP to this “average” structure (Figure 6).

**Figure 6:**
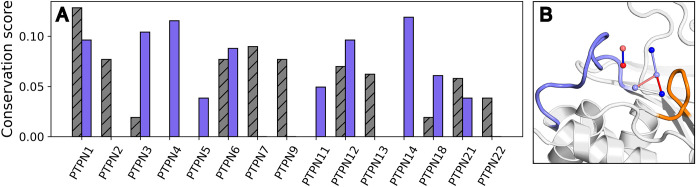
(A) Conservation score to PTP1B closed crystal structure summary network calculated for a set of nonreceptor PTPs using active region waters only. Purple bars correspond to structures with open WPD-loops, and gray hashed bars correspond to structures with closed WPD-loops. (B) Projection of water clusters identified for PTP1B closed crystal structures colored by conservation of particular water positions and by neighbor conservation (blue = less conserved, red = more conserved). WPD-loop is colored blue, and P-loop is colored orange.

We note that this conservation score is not linearly correlated with network density (Figures S2 and S7), confirming that our conservation score is indifferent to inherent water density. Interestingly, we see that when using only combined WPD-loop closed crystal structures as our reference for the “true” PTP1B closed water network structure, there is not a clear trend indicating that the WPD-loop closed structures consistently showcase higher conservation scores. This is only the case for PTPN1 and PTPN21, and we note that this difference in conservation score is very subtle. We further see that PTPN3, PTPN6, PTPN12, and PTPN18 all show higher conservation scores for open structures. We believe this phenomenon is an artifact of the WPD-loop closed crystal structures typically containing either strategic amino acid substitutions or added ligands to crystallize in the closed structure, causing variability in potential water locations, which is in turn reflected in these conservation scores.

Specifically, the WPD-loop open structures of PTPN5 and PTPN21 along with the WPD-loop closed structures of PTPN3, PTPN18, and PTPN22 exhibit very low conservation scores. All of these structures contain either no resolved water molecules near the catalytic cysteine or bulky ions which block this region within the active site, thus providing incomplete information about active site solvation. In this context, we have also tested the impact of active region sphere size (Figure 6A shows data using a 6 Å cutoff for the active region radius) on the conservation score (Figure S8) and demonstrated a correlation between calculated conservation scores and resolution of the structures used for analysis, emphasizing that it is important for the user to take resolution into account when selecting active region radius (more detailed discussion about how the size of the active site region, and resolution more broadly, impacts calculated conservation scores is provided in “Supplementary Technical Considerations” in the Supporting Information and in Figure S9).

Using WatCon, we can also project a calculated conservation for each clustered water to analyze which particular water molecules are most conserved (Figure 6B). We can then supplement this by calculating conservation of nearby clusters. in doing so, we can showcase both how often certain water molecules are conserved within a collection of structures, and also how often two nearby clusters form simultaneously. This can provide us with a useful indication as to how often larger, more complex, networks form (and how often networks form within general locations of interest) without requiring manual examination of every frame or every structure. Furthermore, this component of the tool allows the user to gain insight into which water networks to look for and analyze within individual structures. This in turn would dramatically speed up analysis time. We first note that there are fleeting water positions located around the WPD-loop that are not highly conserved across structures, which makes sense given both how flexible the WPD-loop is, and the variety of different conformations that the corresponding water-interacting WPD-loop residue side-chains can occupy. However, there are highly conserved water positions located on the N-terminal end of the WPD-loop which are present in almost all of the crystal structures, but do not interact directly with water networks deeper within the active site. We can also see that the three water clusters corresponding to the oxygen atoms of the phosphate are not actually highly conserved across all structures (Figure 6B). This is likely due to the presence of inhibitors or other ligands;^100^ since many of the PTP1B closed crystal structures contain a ligand for the purposes of resolving a WPD-loop closed structure, thus blocking water molecules from entering the active site.

We continue by classifying all of our protein–water interactions in tandem with performing a multiple sequence alignment to determine the presence of key structural waters dependent on specific sequence constructions. To demonstrate the use of our tool, we point out the general acid (D181 in PTP1B), which is known to interact with a nucleophilic water during the rate-limiting hydrolysis step of the generalized PTP mechanism (Figure S3). Using our two-angle classification system (explained in Experimental Design), we can effectively classify interacting water molecules as potentially catalytic or noncatalytic. We can first observe that the only structures that could contain a catalytic water interacting with this general acid have a WPD-loop closed position, and our angle classifications reflect this (Figure 7A). Furthermore, the closed structures which do not show this position of water either contain a ligand which blocks this position (PDB ID 2F71 ^101^), contain a general acid substitution (PDB IDs 6KZQ,^102^ 4S0G,^103^ and 4GFU^63^), or contain extremely few crystallized water molecules (PDB ID 2QEP^99^).

**Figure 7:**
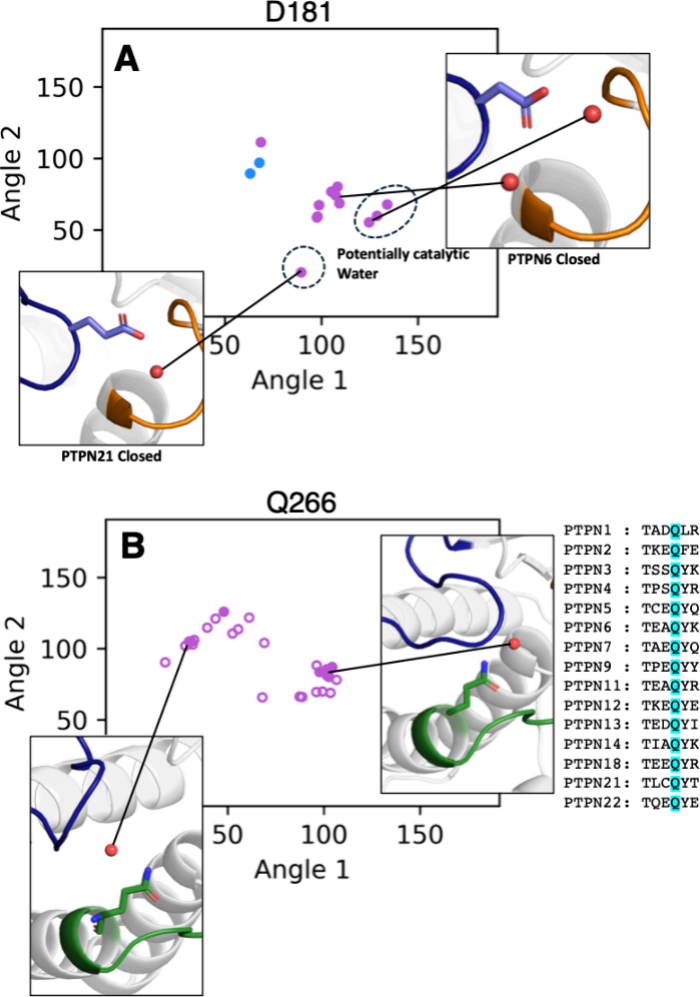
(A) Calculated protein–water angles for nonreceptor PTPs at D181 (general acid, PTP1B numbering). (B) Calculated protein–water angles for nonreceptor PTPs at Q266 (PTP1B numbering). Backbone interactions are shown in blue and side chain interactions shown in purple. Coordinates associated with open WPD-loop structures are shown as open circles and closed WPD-loop structures with filled circles. Angle 1 was calculated as the angle between the closest interacting protein atom of each residue, the water oxygen, and the C_α_ atom of residue L71. Angle 2 was calculated as the angle between the interacting protein atom, the water oxygen, and the C_α_ atom of residue T154.

Additionally, we have analyzed residue Q266 (PTP1B numbering), which is located toward the N-terminal end of the Q-loop and was previously determined to exhibit a substantial number of water interactions in Application 2 (Figures 4 and 7B). This residue is highly conserved, but the neighboring residues are quite variable. Further, since this residue does not need to be modified to ensure that a particular WPD-loop conformation can be resolved, differences in water interactions near this residue could indicate differences in active site conformation due to sequence and conformation alone, rather than artificial amino acid modifications. At this position, we observe that there are two dominant clusters of water positions involving this residue, neither of which is dependent on WPD-loop conformation. Using WatCon, we can evaluate all conserved residue positions in this manner to see if there are specific water–protein interactions which arise from particular sequence characteristics (or by contrast, if there are structural waters which no longer are present if certain residues are mutated), providing a valuable tool for comprehensive water structure analysis in related proteins.

### Example Application 4: Finding Catalytically Important Water Hotspots

Triosephosphate Isomerase (TPI) is an integral enzyme in glycolytic energy production.^104^ TPI has been thoroughly characterized because of its importance in metabolism, its role in human disease, its near perfect efficiency, and its high genetic conservation between animals, plants, fungi, and bacteria (see, e.g., refs 105–108 , among many others). *In vivo*, TPI catalyzes the reversible isomerization of dihydroxyacetone phosphate and glyceraldehyde-3-phosphate. A rare genetic disease called TPI deficiency disrupts this reaction and causes hemolytic anemia and progressive neuromuscular dysfunction leading to premature death.^109,110^ Further, due to its key role in glycolysis, TPI is becoming increasingly appreciated as a cancer target.^111,112^

Biochemically, TPI has been described as a “catalytically perfect” enzyme.^113^ This catalytic perfection is facilitated by a ligand gated conformational change (Figure S10), where a mobile catalytic loop closes over the TPI active site,^106^ creating a solvent-excluded “cage” that is optimally aligned for catalysis. While the importance of creating such a preorganized active site to TPI catalysis has been well-established,^81^ based on structural and simulation analysis, it has been shown that water positioning also plays an important role in determining catalytic TPI activity, with key active site mutations introducing additional water molecules that impair the efficiency of this enzyme based on the number and location of water molecules introduced.^114,115^

Given the importance of water molecules to TPI function and catalysis, as an example application, we have used WatCon to analyze water networks across the TPI family of enzymes. To this end, we have performed detailed analysis comparing a selection of 24 crystal structures from the wild-type TPI from different organisms. All the PDB structures included in the analysis are unliganded and have a resolution of 2.0 Å or lower (Table S3). As in prior examples, structures were aligned using the sequence_processing module, and then the generate_static_networks module was used to create WaterNetwork objects. Since 24 crystal structures pose a substantively lower volume of data than parsing MD trajectories as performed in prior applications, it was possible to computationally efficiently retain the entire protein structure for analysis without the need to truncate to an active region. Clustering was then performed using the hdbscan^61^ algorithm, as described in the Supporting Information.

Of the 197 clusters initially identified using this approach, 40 were found to be highly conserved (conservation score >0.5), with one of the clusters found to have a perfect conservation score of 1.0, i.e. it is perfectly conserved across organisms. Upon further inspection of the TPI structures, we observed that the cluster was very close to residue E105, which is particularly interesting as the p.E105D mutation (formerly known as the p.E104D mutation^116^) is the most common cause of TPI deficiency in humans, being associated with nearly 80% of all cases of TPI deficiency.^116^ Following from this, we then took the clusters generated from the 24 wild-type crystal structures, and overlaid them onto the experimentally resolved structures of human wild-type and E105D TPI (PDB IDs 2JK2 ^116^ and 2VOM,^117^ respectively) (Figure 8). This visualization highlights the disruption of the conserved water network described in prior work,^116^ and demonstrates WatCon’s ability to agnostically identify functional water molecules without user input-driven selection of water molecules and residues. This also further highlights that in addition to its applications in protein engineering, WatCon can be used to identify residue hotspots that could lead to pathogenic mutations through disruption of functionally important water networks.

**Figure 8:**
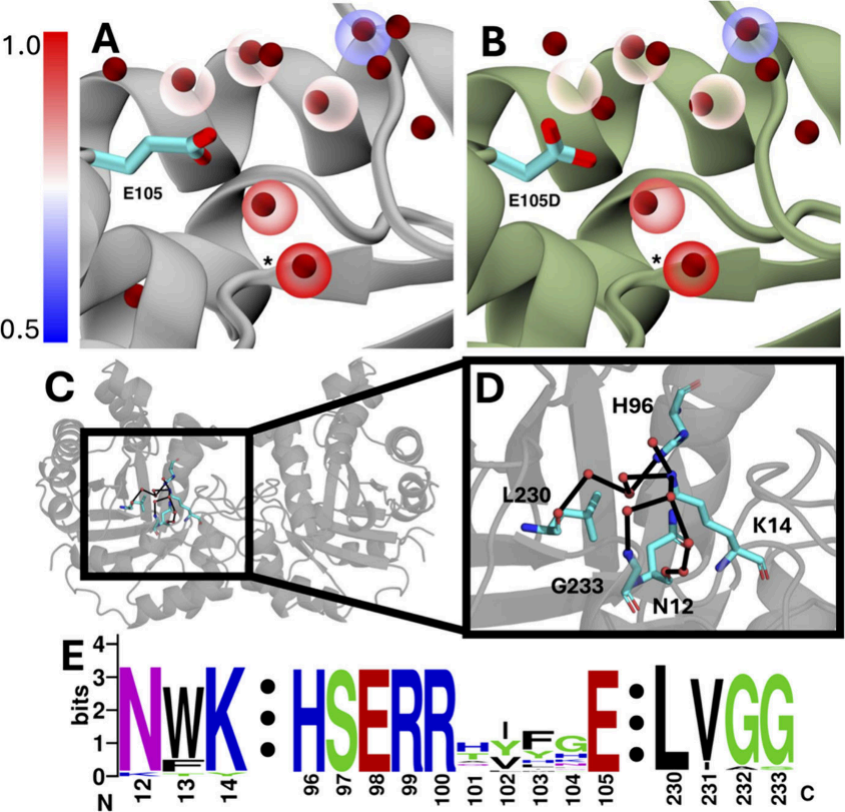
(A) Wild-type human TPI (PDB ID 2JK2 ^117^) with water clusters shown in transparent spheres colored by conservation score. Structural waters are shown in dark red. WatCon identified a water cluster in TPI that was perfectly conserved between 24 organisms (conservation score = 1.0). This cluster (marked with *) is near the side chain of E105, a residue that causes to TPI deficiency when mutated to aspartate.^116^ (B) Identical water clusters shown in A, with E105D mutant TPI (PDB ID 2VOM^117^) aligned. This mutation causes a visible disruption of the conserved network, as described in prior work.^117^ (C) Cartoon representation of the human TPI dimer focused on the active site of chain A. (D) A WatCon calculated water network is shown connecting the side chains of N12, K14, H96, L230, and G233. Water clusters are shown as red spheres with the network connected by black edges. (E) WebLogo^118^ sequence alignment highlighting the high conservation of all residues described in (A–D). Residue indexing based on human TPI. Alignment was performed using the MUSCLE^119^ web server from EMBL-EBI.^120^

Further, we utilized the find_conserved_networks and visualize_structures modules to visualize highly conserved water networks for further analysis. These networks are diverse in size and in conservation. Here, each of the 197 identified water clusters were included. Notably, WatCon identified a large water network located within the active site of TPI. The network connects eight water molecules to five highly conserved residues: N12, K14, H96, L230, and G233 (Figure 8). These residues have separately been reported to be impactful for enzymatic activity. Specifically, they are relevant for oxyanion stabilization, enediolate phosphate stabilization, catalytic proton transfer, active site water regulation as a hydrophobic clamp, and phosphate oxygen coordination respectively (see refs 121 and 122 and references cited therein for further details). This application of WatCon showcases its ability to not only identify water interactions with key catalytic residues, but also identify mutation hotspots for pathogenic mutations that act by disrupting functionally important water networks.

Finally, we note that, for simplicity, our analysis in Figure 8 was performed using only one subunit of each TPI shown in Table S3. This is due to the fact that a number of these structures (PDB IDs 4MKN,^123^ 4Y8F,^124^ 7RCQ,^63^ and 7RPN^63^) contain only the asymmetric unit of the TPI, reconstructing the full water shell reliable from the asymmetric unit poses a significant challenge (success in this area has been had when using biological small-angle X-ray scattering data; see ref 125). By considering only a single TPI chain, this allows us to use the full set of structures shown in Table S3 in our analysis, while avoiding the challenge of reconstructing the full water shell in structures where it is not provided. For comparison, however, Figure S11 shows analysis of the subset of only structures containing the full dimer and associated water shell from the set in Table S3, and shows that the results of this analysis is qualitatively similar. We note that this simplification can be made due to the fact that we are analyzing static structures with retained crystallographic water molecules; for dynamical WatCon analysis, however, it is important to retain the biological assembly to correctly model the behavior of the water network.

### Example Application 5: Tracking Active Site Water Hotspots in Enzymes with Molecular Tunnels

Although our prior example applications have shown a variety of use cases for WatCon, they have specifically focused on enzymes regulated by catalytic loop motions. Most use cases of WatCon will be far more straightforward, and not regulated by complex conformational transitions. However, another challenging use case for WatCon is being able to track the impact of water tunnels (and their alteration) on active site solvation. Such tunnels can be extremely important to enzyme function, for instance, heavily buried enzyme active sites may only be accessible by transportation via tunnels.^126^ For these types of proteins, understanding how targeted modifications alter tunnel transport is crucial to understanding changes in catalytic effects,^126^ both from a fundamental chemistry perspective, and also given the attractiveness of targeting tunnels in protein engineering efforts^127^ and as targets for drug discovery.^128^ Here, we showcase an example of the impact of tunnel modifications on the solvation of a buried active site.

While there exist a variety of enzymes in which tunnels are catalytically important,^126^ we have chosen the haloalkane dehalogenase LinB as our model system to showcase how WatCon can be used to study the impact of engineered tunnels. Haloalkane dehalogenases are biocatalytically important enzymes due to their potential applications in the bioremediation of environmentally toxic industrial byproducts.^129^ The active sites of haloalkane dehalogenases are deeply buried and connected with the surrounding solvent through many tunnels.^130^ Given their biocatalytic importance, haloalkane dehalogenases in general, and LinB included, have been the effort of substantive protein engineering efforts, including attempts to control LinB activity and specificity through targeting tunnel modifications (see, e.g., refs 131–145 , among others).

We note that characterizing enzyme tunnels is challenging, as modeling flux through tunnels becomes important,^146^ and the description of water migration times through tunnels can be sensitive to solvent model used.^147^ For our showcase example, we focus specifically on comparing wild-type LinB with an alternate LinB variant with a blocked native tunnel and *de novo* engineered transport tunnel, which in turn resulted in never previously observed changes in the specificity of this LinB variant.^142^ This variant, named LinB-Open^W^ (now referred to as LinB-Open for simplicity), possessess L177W, W140A, F143L, and I211L amino acid substitutions, which serve to close the native tunnel (typically gated by residues D147 and L177) and open a new tunnel, gated by residues W140 and F143 ^142^ (Figure S12). A previously observed variant was also utilized in their study, named LinB-Closed^W^ (now referred to as LinB-Closed), which has a blocked native tunnel and does not have the modifications to open the new tunnel.^148^ Damborsky and colleagues impressively demonstrated the impacts of these modifications on the size and presence of both tunnels.^142^ Here we extend that analysis as a showcase application of WatCon.

To facilitate our analysis, we conducted 3 × 300 ns molecular dynamics simulations for LinB-WT (PDB ID 1MJ5 ^148^), LinB-Open (PDB ID 5LKA^142^) and LinB-Closed (PDB ID 4WDQ^148^). We note that, importantly, for the purposes of this application, we use WatCon to track active site water hotspots in conjunction with already well-characterized tunnels. For *de novo* tunnel identification and characterization, WatCon can be coupled with tools such as CAVER^149^ and AQUA-DUCT,^146,150^ to identify and characterize water flux through the tunnels, respectively, although we encourage care when selecting a water model.^147^ Based on our analysis, we demonstrate that the LinB-Open and LinB-Closed variants demonstrate notably different internal solvation structures, which certainly contribute to the differences in catalytic efficiency among the variants.

We first begin by analyzing the water hotspots within the buried active site of the LinB variants. Immediately, we observe that the wild-type protein has a much broader distribution of waters than either of the two variants (Figure 9). Specifically, the LinB-Closed variant appears to have the weakest distribution of waters within the active site (albeit subtly compared to the LinB-Open variant), as expected and consistent with the original engineering study.^142^ We further notice that the densities of the connected graphs are lowest for the LinB-Closed structure and highest for the wild-type structures. Importantly, however, the LinB-Open variant shows the most consistent interactions with catalytic residues D108 and H272 compared to either of other two structures. In other words, despite the larger distribution of water positions within the active site of the wild-type protein, the modified LinB-Open structure consistently allows for more water–protein interactions among both D108 and H272. The combination of these results, therefore, indicate that the adjusted tunnels prevent widespread solvation of the active site and instead allow for more consistent interactions with catalytic residues D108 and H272, likely contributing to the higher catalytic prowess of the LinB-Open variant compared to the wild-type structure.^142^ Taken together, these five applications showcase the diverse use cases of WatCon as a tool to understand the role of water molecules in protein evolution, identify hotspots for protein engineering, and expand our understanding of the role of perturbed water in human disease.

**Figure 9:**
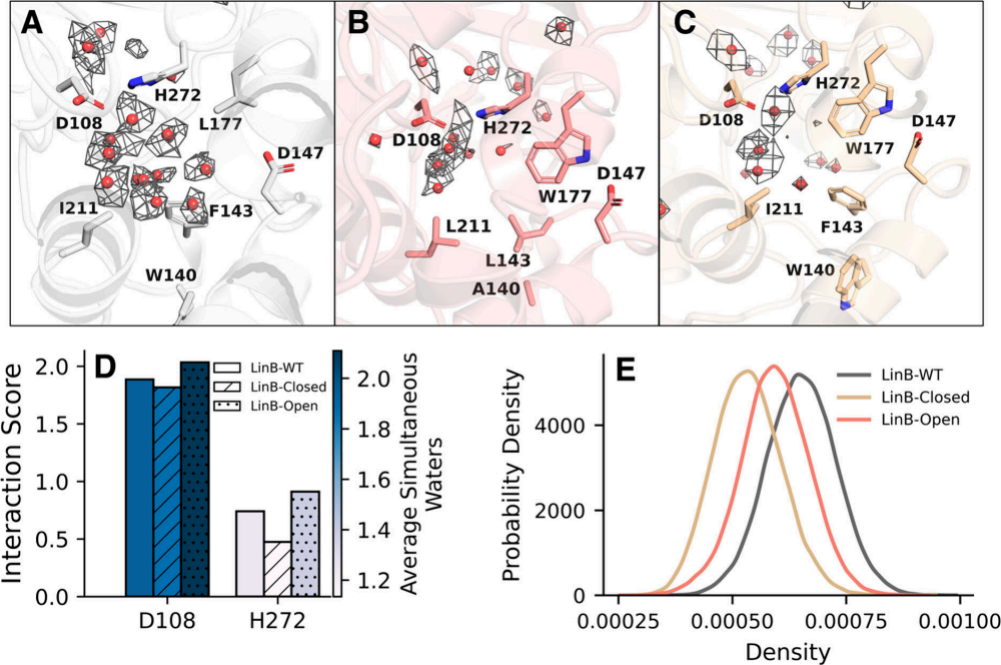
(A–C) Projections of water density hotspots obtained from simulations performed on the (A) WT LinB, (B) LinB-Open^W^, and (C) LinB-Closed^W^ structures. Densities are visualized at 1.5σ, and red spheres indicate points of highest density. (D) Calculated interaction scores for catalytic residues D108 and H272. (E) Calculated graph density for each variant. Results were calculated for molecular dynamics trajectories of 3 replicas × 300 ns for each variant.

## Discussion

We introduce a Python-based tool, WatCon, which can be utilized to perform a variety of water network analyses on both experimental structures and/or simulation trajectories. WatCon can be used either as a standalone tool or can, in principle, easily be applied within a pipeline of further analysis. WatCon’s interface between MDAnalysis^52,53^ and NetworkX^55^ allows for ease in user-desired modification. In addition to presenting the tool, we have showcased a range of applications of WatCon to analyzing water networks within both individual proteins and protein families, focusing on triosephosphate isomerases and protein tyrosine phosphatases as model systems, as well as additional technical considerations provided in the Supporting Information, including a detailed overview of WatCon pitfalls and caveats to consider. We further present a showcase application using WatCon to track water hotspots in native and engineered water tunnels in dehalogenases. These example applications illustrate the ability of WatCon to track water molecules, compare static and dynamic water networks, compare individual proteins with a family to a consensus water network for the family, and to identify functionally important water hotspots. In doing so, we demonstrate that WatCon is a powerful tool to understand the role of water molecules in protein evolution, predict hotspots for protein engineering, and even predict hotspots for pathogenic mutations that act by disrupting water networks. WatCon is an open-source package, available under the GNU General Public License v3.0 (GPLv3) license, the source code for which can be found at https://github.com/kamerlinlab/WatCon.

## Supplementary Material



## Data Availability

WatCon source code is available at https://github.com/kamerlinlab/WatCon, and a simulation data pack to support our analysis is available on Zenodo at DOI: 10.5281/zenodo.15213224.

## Notes

Additional simulation details, additional technical considerations, water analysis, and lists of PDB IDs used for analysis in this work (PDF)

## References

[1] Bellissent-Funel M.-C., Hassanali A., Havenith M., Henchman R., Pohl P., Sterpone F., van der Spoel D., Xu Y., Garcia A. E. (2016). Water Determines
the Structure and Dynamics of Proteins. Chem.
Rev..

[2] Finney J. L. (1977). The Organization
and Function of Water in Protein Crystals. Philos.
Trans. R. Soc. London, Ser. B.

[3] Schoenborn B. P., Garcia A., Knott R. (1995). Hydration in Protein Crystallography. Prog. Biophys. Mol. Biol..

[4] Bryant R. G. (1996). The Dynamics
of Water-Protein Interactions. Annu. Rev. Biophys.
Biomol. Struct..

[5] Schwabe J. W. R. (1997). The
Role of Water in Protein-DNA Interactions. Curr.
Opin. Struct. Biol..

[6] Mattos C. (2002). Protein-Water
Interactions in a Dynamic World. Trends Biochem.
Sci..

[7] Papoian G. A., Ulander J., Eastwood M. P., Luthey-Schulten Z., Wolynes P. G. (2004). Water in Protein Structure Prediction. Proc. Natl. Acad. Sci. U.S.A..

[8] Rand R. P. (2004). Probing
the Role of Water in Protein Conformation and Function. Philos. Trans. R. Soc. London, Ser. B.

[9] Nakasako M. (2004). Water-Protein
Interactions form High-Resolution Protein Crystallography. Philos. Trans. R. Soc. London, Ser. B.

[10] Levy Y., Onuchic J. N. (2004). Water and Proteins:
A Love-Hate Relationship. Proct. Natl. Acad.
Sci. U. S. A..

[11] Raschke T. M. (2006). Water Structure
and Interactions With Protein Surfaces. Curr.
Opin. Struct. Biol..

[12] Doster W., Gutberlet T. (2010). The Protein-Water Energy Seascape. Biochim. Biophys. Acta.

[13] Ahmed M. H., Spyrakis F., Cozzini P., Tripathi P. K., Mozzarelli A., Scarsdale J. N., Safo M. A., Kellogg G. E. (2011). Bound Water
At Protein-Protein
Interfaces: Partners, Roles and Hydrophobic Bubbles as a Conserved
Motif. PLoS One.

[14] Vajda T., Perczel A. (2014). Role of Water in Protein Folding,
Oligomerization,
Amyloidosis and Miniprotein. Pept. Sci..

[15] Gerwert K., Freier E., Wolf S. (2014). The Role of
Protein-Bound Water Molecules
in Microbial Rhodopsins. Biochim. Biophys. Acta,
Bioenerg..

[16] Carugo O. (2015). Structure
and Function of Water Molecules Buried in the Protein Core. Curr. Protein Pept. Sci..

[17] Chong S.-H., Ham S. (2017). Dynamics of Hydration
Water Plays a Key Role in Determining the Binding
Thermodynamics of Protein Complexes. Sci. Rep..

[18] Schiebel J., Gaspari R., Wulsdorf T., Ngo K., Sohn C., Schrader T. E., Cavalli A., Ostermann A., Heine A., Klebe G. (2018). Intriguing Role of Water in Protein-Ligand
Binding Studied by Neutron Crystallography on Trypsin Complexes. Nat. Commun..

[19] Maurer M., Oostenbrink C. (2019). Water in Protein Hydration and Ligand
Recognition. J. Mol. Recognit..

[20] Schirò G., Weik M. (2019). Role of Hydration Water
in the Onset of Protein Structural Dynamics. J. Phys.: Condens. Matter.

[21] Mukherjee S., Nithin C., Divakaruni Y., Bahadur R. P. (2019). Dissecting Water
Binding Sites at Protein-Protein Interfaces: A Lesson From the Atomic
Structures in the Protein Data Bank. J. Biomol.
Struct. Dyn..

[22] Crilly C. J., Eicher J. E., Warmuth O., Atkin J. M., Pielak G. J. (2021). Water’s
Variable Role in Protein Stability Uncovered by Liquid-Observed Vapor
Exchange NMR. Biochemistry.

[23] Samways M. L., Bruce Macdonald H. E., Taylor R. D., Essex J. W. (2023). Water Networks in
Complexes Between Proteins and FDA-Approved Drugs. J. Chem. Inf. Model..

[24] Hishida M., Kaneko A., Yamamura Y., Saito K. (2023). Contrasting
Changes
in Strongly and Weakly Bound Hydration Water of a Protein upon Denaturation. J. Phys. Chem. B.

[25] Qing R., Hao S., Smorodina E., Jin D., Zalevsky A., Zhang S. (2022). Protein Design:
From the Aspect of Water Solubility and Stability. Chem. Rev..

[26] de
Beer S. B. A., Vermeulen N. P. E., Oostenbrink C. (2010). The Role of
Water Molecules in Computational Drug Design. Curr. Top. Med. Chem..

[27] López E. D., Arcon J. P., Gauto D. F., Petruk A. A., Modenutti C. P., Dumas V. G., Marti M. A., Turjanski A. G. (2015). WATCLUST:
A Tool for Improving the Design of Drugs Based on Protein-Water Interactions. Bioinformatics.

[28] Jukič M., Konc J., Gobec S., Janežič D. (2017). Identification
of Conserved Water Sites in Protein Structures for Drug Design. J. Chem. Inf. Model..

[29] Spyrakis F., Ahmed M. H., Bayden A. S., Cozzini P., Mozzarelli A., Kellogg G. E. (2017). The Roles of Water in the Protein
Matrix: A Largely
Untapped Resource for Drug Discovery. J. Med.
Chem..

[30] Schaller D., Pach S., Wolber G. (2019). PyRod: Tracing Water Molecules in
Molecular Dynamics Simulations. J. Chem. Inf.
Model..

[31] Sobhia M. E., Ghosh K., Kumar G. S., Sivangula S., Laddha K., Kumari S., Kumar H. (2022). The Role of Water Network
Chemistry in Proteins: A Structural Bioinformatics Perspective in
Drug Discovery and Development. Curr. Top. Med.
Chem..

[32] Morningstar-Kywi N., Wang K., Asbell T. R., Wang Z., Giles J. B., Lai J., Brill D., Sutch B. T., Haworth I. S. (2022). Prediction of Water
Distributions and Displacement at Protein-Ligand Interfaces. J. Chem. Inf. Model..

[33] Tošović J., Fijan D., Jukič M., Bren U. (2022). Conserved Water Networks
Identification for Drug Design Using Density Clustering Approaches
on Positional and Orientational Data. J. Chem.
Inf. Model..

[34] Darby J. F., Hopkins A. P., Shimizu S., Roberts S. M., Brannigan J. A., Turkenburg J. P., Thomas G. H., Hubbard R. E., Fischer M. (2019). Water Networks
Can Determine the Affinity of Ligand Binding to Proteins. J. Am. Chem. Soc..

[35] Stachowski T. R., Vanarotti M., Seetharaman J., Lopez K., Fischer M. (2022). Water Networks
Repopulate Protein-Ligand Interfaces With Temperature. Angew. Chem., Int. Ed..

[36] Patel H., Grüning B. A., Günther S., Merfort I. (2014). PyWATER: A PyMOL Plug-In
to Find Conserved Water Molecules in Proteins By Clustering. Bioinformatics.

[37] Fusani L., Wall I., Palmer D., Cortes A. (2018). Optimal Water
Networks
in Protein Cativities with GAsol and 3D-RISM. Bioinformatics.

[38] Cuzzolin A., Deganutti G., Salmaso V., Sturlese M., Moro S. (2018). AquaMMapS:
An Alternative Tool to Monitor the Role of Water Molecules During
Protein-Ligand Association. ChemMedChem.

[39] Nittinger E., Gibbons P., Eigenbrot C., Davies D. R., Maurer B., Yu C. L., Kiefer J. R., Kuglstatter A., Murray J., Ortwine D. F. (2019). Water
Molcules in Protein-Ligand
Interfaces. Evaluation of Software Tools and SAR Comparison. J. Comput.-Aided Mol. Des..

[40] Zamanos A., Ioannakis G., Emiris I. Z. (2024). HydraProt: A New Deep Learning Tool
for Fast and Accurate Prediction of Water Molecule Positions for Protein
Structures. J. Chem. Inf. Model..

[41] Krieger J. M., Doljanin F., Bogetti A. T., Zhang F., Manivarma T., Bahar I., Mikulska-Ruminska K. (2024). WatFinder:
A ProDy Tool for Protein–Water
Interactions. Bioinformatics.

[42] Zitnik M., Li M. M., Wells A., Glass K., Morselli
Gysi D., Krishnan A., Murali T. M., Radivojac P., Roy S., Baudot A. (2024). Current and Future Directions in Network Biology. Bioinf. Adv..

[43] Böde C., Kovács I. A., Szalay M. S., Palotai R., Korcsmáros T., Csermely P. (2007). Network Analysis of Protein Dynamics. FEBS Lett..

[44] Sharan R., Ulitsky I., Shamir R. (2007). Network-Based Prediction
of Protein
Function. Mol. Syst. Biol..

[45] Yehorova D., Di Geronimo B., Robinson M., Kasson P. M., Kamerlin S. C. L. (2024). Using
Residue Interaction Networks (RINs) to Understand Protein Evolution
and Engineer New Proteins. Curr. Opin. Struct.
Biol..

[46] Bertalan E., Bondar A.-N. (2023). Graphs of Protein-Water
Hydrogen Bond Networks to Dissect
Structural Movies of Ion-Transfer Microbial Rhodopsins. Front. Chem..

[47] Qu X., Dong L., Luo D., Si Y., Wang B. (2024). Water Network-Augmented
Two-State Model for Protein–Ligand Binding Affinity Prediction. J. Chem. Inf. Model..

[48] Kuang X., Su Z., Liu Y. L., Lin X., Spencer-Smith J., Derr T., Wu Y., Meiler J. (2024). SuperWater:
Predicting
Water Molecule Positions on Protein Structures by Generative AI. bioRxiv.

[49] Yehorova D., Crean R. M., Kasson P. M., Kamerlin S. C. L. (2024). Key Interaction
Networks: Identifying Evolutionarily Conserved Non-Covalent Interaction
Networks Across Protein Families. Protein Sci..

[50] Brysbaert G., Blossey R., Lensink M. F. (2018). The Inclusion
of Water Molecules
in Residue Interaction Networks Identifies Additional Central Residues. Front. Mol. Biosci..

[51] Van Rossum, G. ; Drake, F. L. Python 3 Reference Manual; CreateSpace, 2009.

[52] Michaud-Agrawal N., Denning E. J., Woolf T. B., Beckstein O. (2011). MDAnalysis:
A Toolkit for the Analysis of Molecular Dynamics Simulations. J. Comput. Chem..

[53] Gowers, R. ; Linke, M. ; Barnoud, J. ; Reddy, T. ; Melo, M. ; Seyler, S. ; Domanski, J. ; Dotson, D. ; Buchoux, S. ; Kenney, I. ; MDAnalysis: A Python Package for the Rapid Analysis of Molecular Dynamics Simulations. In Proceedings of the 15th Python in Science Conference; SciPy.org, 2016. 10.25080/Majora-629e541a-00e.

[54] Webb B., Šali A. (2016). Comparative Protein Structure Modeling Using Modeller. Curr. Protoc. Bioinf..

[55] Hagberg, A. A. ; Schult, D. A. ; Swart, P. J. Exploring Network Structure, Dynamics and Function Using NetworkX. In Proceedings of the 7th Python in Science Conference; SciPy.org, 2008. 10.25080/TCWV9851.

[56] Harris C. R., Millman K. J., van der
Walt S. J., Gommers R., Virtanen P., Cournapeau D., Wieser E., Taylor J., Berg S., Smith N. J. (2020). Array Programming with NumPy. Nature.

[57] pandas-dev/pandas: Pandas; Zenodo, 2020.

[58] Pedregosa F., Varoquaux G., Gramfort A., Michel V., Thirion B., Grisel O., Blondel M., Prettenhofer P., Weiss R., Dubourg V. (2011). Scikit-learn: Machine
Learning in Python. J. Mach. Learn. Res..

[59] Virtanen P., Gommers R., Oliphant T. E., Haberland M., Reddy T., Cournapeau D., Burovski E., Peterson P., Weckesser W., Bright J. (2020). SciPy 1.0: Fundamental
Algorithm for Scientific Computing in Python. Nat. Methods.

[60] Ester, M. ; Kriegel, H.-P. ; Sander, J. ; Xu, X. A Density-Based Algorithm for Discovering Clusters in Large Spatial Databases with Noise. In KDD’96: Proceedings of the Second International Conference on Knowledge Discovery and Data Mining; AAAI Press, 1996; pp 226–231.

[61] McInnes L., Healy J., Astels S. (2017). hdbscan: Hierarchical density based
clustering. J. Open Source Software.

[62] Ankerst M., Breunig M. M., Kriegel H.-P., Sander J. (1999). OPTICS: Ordering Points
to Identify the Clustering Structure. ACM SIGMOD
Rec..

[63] Berman H. M., Westbrook J., Feng Z., Gilliland G., Bhat T. N., Weissig H., Shindyalov I. N., Bourne P. E. (2000). The Protein Data Bank. Nucleic
Acids Res..

[64] Sievers F., Wilm A., Dineen D., Gibson T. J., Karplus K., Li W., Lopez R., McWilliam H., Remmert M., Söding J. (2011). Fast, Scalable Generation of High-Quality Protein Multiple Sequence
Alignments Using Clustal Omega. Mol. Syst. Biol..

[65] Jumper J., Evans R., Pritzel A., Green T., Figurnov M., Ronneberger O., Tunyasuvunakool K., Bates R., Žídek A., Potapenko A. (2021). Highly Accurate Protein Structure Prediction
with AlphaFold. Nature.

[66] Mills J. E. J., Dean P. M. (1996). Three-Dimensional
Hydrogen-Bond Geometry and Probability
Information from a Crystal Survey. J. Comput.-Aided.
Mol. Des..

[67] Siemers M., Lazaratos M., Karathanou K., Guerra F., Brown L. S., Bondar A.-N. (2019). Bridge:
A GraphBased Algorithm to Analyze Dynamic H-Bond
Networks in Membrane Proteins. J. Chem. Theory
Comput..

[68] The PyMOL Molecular Graphics System; Schrödinger, LLC.

[69] Benzi M., Daidone I., Faccio C., Zanetti-Polzi L. (2022). Structural
Analysis of Water Networks. J. Complex Networks.

[70] Bartoli L., Fariselli P., Casadio R. (2007). The Effect of Backbone on the Small-World
Properties of Protein Contact Maps. Phys. Biol..

[71] Ponti A., Candelieri A., Giordani I., Archetti F. (2021). Probabilistic Measures
of Edge Criticality in Graphs: A Strudy in Water Distribution Networks. Appl. Network Sci..

[72] Meng W. S., von Grafenstein H., Haworth I. S. (2000). Water Dynamics at
the Binding Interface
of Four Different HLA-A2-Peptide Complexes. Int. Immunol..

[73] Doyle M. D., Bhowmick A., Wych D. C., Lassalle L., Simon P. S., Holton J., Sauter N. K., Yachandra V. K., Kern J. F., Yano J. (2023). Water Networks in Photosystem
II Using Crystalline Molecular Dynamics Simulations and Room-Temperature
XFEL Serial Crystallography. J. Am. Chem. Soc..

[74] Karvounis G., Nerukh D., Glen R. C. (2004). Water Network
Dynamics At the Critical
Moment of a Peptide’s β-Turn Formation: A Molecular Dynamics
Study. J. Chem. Phys..

[75] Law P. B., Daggett V. (2010). The Relationship Between
Water Bridges and the Polyproline
II Conformation: A Large-Scale Analysis of Molecular Dynamics Simulations
and Crystal Structures. Protein Eng. Des. Sel..

[76] Singh H., Vasa S. K., Jangra H., Rovó P., Päslack C., Das C. K., Zipse H., Schäfer L. V., Linser R. (2019). Fast Microsecond Dynamics of the
Protein–Water
Network in the Active Site of Human Carbonic Anhydrase II Studied
by Solid-State NMR Spectroscopy. J. Am. Chem.
Soc..

[77] Gelenter M. D., Mandala V. S., Niesen M. J. M., Sharon D. A., Dregni A. J., Willard A. P., Hong M. (2021). Water Orientation and Dynamics in
the Closed and Open Influenza B Virus M2 Proton Channels. Commun. Biol..

[78] Klyshko E., Kim J. S.-H., Rauscher S. (2022). LAWS: Local Alignment
for Water Sites—Tracking
Ordered Water Simulations. bioRxiv.

[79] McGibbon R. T., Beauchamp K. A., Harrigan M. P., Klein C., Swails J. M., Hernández C. X., Schwantes C. R., Wang L.-P., Lane T. J., Pande V. S. (2015). MDTraj:
A Modern Open Library for the Analysis of Molecular
Dynamics Trajectories. Biophys. J..

[80] Ladbury J. E. (1996). Just Add
Water! The Effect of Water on the Specificity of Protein-Ligand Binding
Sites and Its Potential Application to Drug Design. Chem. Biol..

[81] Richard J. P., Amyes T. L., Goryanova B., Zhai X. (2014). Enzyme Architecture:
On the Importance of Being in a Protein Cage. Curr. Opin. Struct. Biol..

[82] Tonks N. K. (2023). Protein
Tyrosine Phosphatases: Mighty Oaks From Little Acorns Grow. IUBMB Life.

[83] Tonks N. K. (2003). PTP1B:
From the Sidelines to the Front Lines!. FEBS
Lett..

[84] Cui D. S., Lipchock J. M., Brookner D., Loria J. P. (2019). Uncovering the Molecular
Interactions in the Catalytic Loop That Modulate the Conformational
Dynamics in Protein Tyrosine Phosphatase 1B. J. Am. Chem. Soc..

[85] Keng Y. F., Wu L., Zhang Z. Y. (1999). Probing the Function of the Conserved Tryptophan in
the Flexible Loop of the Yersinia Protein-Tyrosine Phosphatase. Eur. J. Biochem..

[86] Torgeson K. R., Clarkson M. W., Granata D., Lindorff-Larsen K., Page R., Peti W. (2022). Conserved Conformational
Dynamics
Determine Enzyme Activity. Sci. Adv..

[87] Choi J.-H., Lee H., Choi H. R., Cho M. (2018). Graph Theory and Ion and Molecular
Aggregation in Aqueous Solutions. Annu. Rev.
Phys. Chem..

[88] Brandão T. A. S., Hengge A. C., Johnson S. J. (2010). Insights into the Reaction of Protein-Tyrosine
Phosphatase 1B. J. Biol. Chem..

[89] Liu M., Wang L., Sun X., Zhao X. (2014). Investigating the Impact
of Asp181 Point Mutations on Interactions Between PTP1B and Phosphotyrosine
Substrate. Sci. Rep..

[90] Lipchock J. M., Hendrickson H. P., Douglas B. B., Bird K. E., Ginther P. S., Rivalta I., Ten N. S., Batista V. S., Loria J. P. (2017). Characterization
of Protein Tyrosine Phosphatase 1B Inhibition by Chlorogenic Acid
and Cichoric Acid. Biochemistry.

[91] Xiao P., Wang X., Wang H.-M., Fu X.-L., Cui F., Yu X., Wen S., Bi W.-X., Sun J.-P. (2014). The Second-Sphere
Residue T263 Is Important for the Function and Catalytic Activity
of PTP1B *via* Interaction with the WPD-Loop. Int. J. Biochem. Cell Biol..

[92] Crean R. M., Biler M., van der Kamp M. W., Hengge A. C., Kamerlin S. C. L. (2021). Loop
Dynamics and Enzyme Catalysis in Protein Tyrosine Phosphatases. J. Am. Chem. Soc..

[93] Caldararu O., Misini Ignjatović M., Oksanen E., Ryde U. (2020). Water Structure
in Solution and Crystal Molecular Dynamics Simulations Compared to
Protein Crystal Structures. RSC Adv..

[94] Carugo O., Bordo D. (1999). How Many Water Molecules
Can Be Detected By Protein Crystallography?. Acta Crystallogr., Sect. D: Biol. Crystallogr..

[95] Pedersen A. K., Peters G. H., Møller K. B., Iversen L. F., Kastrup J. S. (2004). Water-Molecule
Network and Active-Site Flexibility of Apo Protein Tyrosine Phosphatase
1B. Acta Crystallogr., Sect. D: Biol. Crystallogr..

[96] Zhang Z. Y., Wang Y., Dixon J. E. (1994). Dissecting
the Catalytic Mechanism
of Protein-Tyrosine Phosphatases. Proc. Natl.
Acad. Sci. U. S. A..

[97] Tabernero L., Aricescu A. R., Jones E. Y., Szedlacsek S. E. (2008). Protein
Tyrosine Phosphatases: Structure-Function Relationships. FEBS J..

[98] Alonso A., Pulido R. (2016). The Extended Human
PTPome: A Growing Tyrosine Phosphatase
Family. FEBS J..

[99] Barr A. J., Ugochukwu E., Lee W. H., King O. N., Filippakopoulos P., Alfano I., Savitsky P., Burgess-Brown N. A., Muller S., Knapp S. (2009). Large-Scale Structural Analysis of
the Classical Human Protein Tyrosine Phosphatome. Cell.

[100] Zhang S., Liu S., Tao R., Wei D., Chen L., Shen W., Yu Z. H., Wang L., Jones D. R., Dong X. C. (2012). A Highly Selective and
Potent PTP-MEG2 Inhibitor with Therapeutic Potential for Type 2 Diabetes. J. Am. Chem. Soc..

[101] Klopfenstein S. R., Evdokimov A. G., Colson A.-O., Fairweather N. T., Neuman J. J., Maier M. B., Gray J. L., Gerwe G. S., Stake G. E., Howard B. W. (2006). 1,2,3,4-Tetrahydroisoquinolinyl
Sulfamic Acids as Phosphatase PTP1B Inhibitors. Bioorg. Med. Chem. Lett..

[102] Xu Y. F., Chen X., Yang Z., Xiao P., Liu C. H., Li K. S., Yang X. Z., Wang Y. J., Zhu Z. L., Xu Z. G. (2021). PTP-MEG2 Regulates Quantal
Size and Fusion Pore Opening Through Two Distinct Structural Bases
and Substrates. EMBO Rep..

[103] Chen K. E., Li M. Y., Chou C. C., Ho M. R., Chen G. C., Meng T. C., Wang A. H. (2015). Substrate
Specificity
and Plasticity of FERM-Containing Protein Tyrosine Phosphatases. Structure.

[104] Reynolds S. J., Yates D. W., Pogson C. I. (1971). Dihydroxyacetone
Phosphate. Its Structure and Reactivity with Glycerophosphate Dehydrogenase,
Aldolase and Triosephosphate Isomerase and Some Possible Metabolic
Implications. Biochem. J..

[105] Wierenga R. K. (2001). The TIM-Barrel Fold: A Versatile
Framework for Efficient
Enzymes. FEBS Lett..

[106] Wierenga R. K., Kapetaniou E. G., Venkatesan R. (2010). Triosephosphate
Isomerase: A Highly Evolved Biocatalyst. Cell.
Mol. Life Sci..

[107] Malabanan M. M., Amyes T. L., Richard J. P. (2010). A Role for Flexible
Loops in Catalysis. Curr. Opin. Struct. Biol..

[108] Myers T. D., Palladino M. J. (2023). Newly Discovered
Roles of Triosephosphate
Isomerase Including Functions Within the Nucleus. Mol. Med..

[109] Schneider A. S. (2000). Triosephosphate
Isomerase Deficiency: Historical Perspectives
and Molecular Aspects. Baillieres Best Pract.
Res. Clin. Haematol..

[110] Orosz F., Oláh J., Ovádi J. (2006). Triosephosphate
Isomerase Deficiency: Facts and Doubts. IUBMB
Life.

[111] Pekel G., Ari F. (2020). Therapeutic Targeting of Cancer Metabolism
with Triosephosphate Isomerase. Chem. Biodiversity.

[112] Enríquez-Flores S., De la Mora-De
la Mora I., García-Torres I., Flores-López L.
A., Martínez-Pérez Y., López-Velázquez G. (2023). Human Triosephosphate Isomerase Is
a Potential Target in Cancer Due to Commonly Occurring Post-Translational
Modifications. Molecules.

[113] Knowles J. R., Albery W. J. (1977). Perfection in Enzyme
Catalysis: The
Energetics of Triosephosphate Isomerase. Acc.
Chem. Res..

[114] Richard J. P., Amyes T. L., Malabanan M. M., Zhai X., Kim K. J., Reinhardt C. J., Wierenga R. K., Drake E. J., Gulick A. M. (2016). Structure-Function
Studies of Hydrophobic Residues That Clamp a Basic Glutamate Side
Chain during Catalysis by Triosephosphate Isomerase. Biochemistry.

[115] Kulkarni Y. S., Liao Q., Petrovic D., Krüger D. M., Strodel B., Amyes T. L., Richard J. P., Kamerlin S. C. L. (2017). Enzyme
Architecture: Modeling the Operation of a Hydrophobic Clamp in Catalysis
by Triosephosphate Isomerase. J. Am. Chem. Soc..

[116] Selamioğlu A., Karaca M., Balcı M. C., Körbeyli H. K., Durmuş A., Yıldız E. P., Karaman S., Gökçay G. F. (2023). Triosephosphate
Isomerase Deficiency: E105D Mutation in Unrelated Patients and Review
of the Literature. Mol. Syndromol..

[117] Rodríguez-Almazán C., Arreola R., Rodríguez-Larrea D., Aguirre-López B., de Gómez-Puyou M. T., Pérez-Montfort R., Costas M., Gómez-Puyou A., Torres-Larios A. (2008). Structural
Basis of Human Triosephosphate Isomerase Deficiency: Mutation E104D
is Related to Alterations of a Conserved Water Network at the Dimer
Interface. J. Biol. Chem..

[118] Crooks G. e., Hon G., Chandonia J.-M., Brenner S. E. (2004). WebLogo: A Sequence Logo Generator. Genome Res..

[119] Edgar R. C. (2004). MUSCLE:
A Multiple Sequence Alignment With High Accuracy
and High Throughput. Nucleic Acids Res..

[120] Madeira F., Madhusoodanan N., Lee J., Eusebi A., Niewielska A., Tivey A. R. N., Lopez R., Butcher S. (2024). The EMBL-EBI
Job Dispatcher Sequence Analysis Tools Framework in 2024. Nucleic Acids Res..

[121] Mande S. C., Hol W. G.J., Mainfroid V., Goraj K., Martial J. A., Kalk K. H. (1994). Crystal Structure
of Recombinant Human Triosephosphate Isomerase at 2.8 Å Resolution.
Triosephosphate Isomerase-Related Human Genetic Disorders, and Comparison
with the Trypanosomal Enzyme. Protein Sci..

[122] Kulkarni Y. S., Amyes T. L., Richard J. P., Kamerlin S. C. L. (2019). Uncovering
the Role of Key Active-Site Side Chains in Catalysis: An Extended
Brønsted Relationship for Substrate Deprotonation Catalyzed by
Wild-Type and Variants of Triosephosphate Isomerase. J. Am. Chem. Soc..

[123] Zaffagnini M., Michelet L., Sciabolini C., Di Giacinto N., Morisse S., Marchand C. H., Trost P., Fermani S., Lemaire S. D. (2014). High-Resolution Crystal Structure
and Redox Properties of Chloroplastic Triosephosphate Isomerase from *Chlamydomonas reinhardtii*. Mol. Plant.

[124] Romero-Romero S., Costas M., Rodríguez-Romero A., Fernández-Velasco D. A. (2015). Reversibility
and Two State Behaviour
in the Thermal Unfolding of Oligomeric TIM Barrel Proteins. Phys. Chem. Chem. Phys..

[125] Prior C., Davies O. W., Bruce D., Pohl E. (2020). Obtaining
Tertiary Protein Structures by the Ab Initio Interpretation of X-ray
Scattering Data. J. Chem. Theory Comput..

[126] Kingsley L. J., Lill M. A. (2015). Substrate Tunnels
in Enzymes: Structure-Function
Relationships and Computational Methodology. Protein Struct., Funct. Bioinf..

[127] Kokkonen P., Bednar D., Pinto G., Prokop Z., Damborsky J. (2019). Engineering Enzyme Access Tunnels. Biotechnol. Adv..

[128] Marques S. M., Daniel L., Buryska T., Prokop Z., Brezovsky J., Damborsky J. (2017). Enzyme Tunnels
and Gates As Relevant
Targets in Drug Design. Med. Res. Rev..

[129] Koudelakova T., Bidmanova S., Dvorak P., Pavelka A., Chaloupkova R., Prokop Z., Damborsky J. (2013). Haloalkane
Dehalogenases: Biotechnological Applications. Biotechnol. J..

[130] Petrek M., Otyepka M., Banas M., Kosinova P., Koca J., Damborsky J. (2006). CAVER: A New Tool to Explore Routes
from Protein Clefts, Pockets and Cavities. BMC
Bioinf..

[131] Chaloupková R., Sýkorová J., Prokop Z., Jesenská A., Monincová M., Pavlová M., Tsuda M., Nagata Y., Damborský J. (2003). Modification
of Activity and Specificity of Haloalkane Dehalogenase from *Sphinogomonas paucimobilis* UT26 By Engineering of
its Entrance TunnelP. J. Biol. Chem..

[132] Nagata Y., Prokop Z., Marvanová S., Sýkorová J., Monincová M., Tsuda M., Damborský J. (2003). Reconstruction of Mycobacterial Dehalogenase
Rv2579 by Cumulative Mutagenesis of Haloalkane Dehalogenase LinB. Appl. Environ. Microbiol..

[133] Janssen D. B. (2004). Evolving Haloalkane Dehalogenases. Curr. Opin. Chem. Biol..

[134] Kmunícek J., Hynková K., Jedlicka T., Nagata Y., Negri A., Gago F., Wade R. C., Damborský J. (2005). Quantitative
Analysis of Substrate Specificity of Haloalkane Dehalogenase LinB
from *Sphingomonas paucimobilis* UT26. Biochemistry.

[135] Banás P., Otyepka M., Jerábek P., Petrek M., Damborský J. (2006). Mechanism
of Enhanced Conversion
of 1,2,3-Trichloropropane by Mutant Haloalkane Dehalogenase Revealed
by Molecular Modeling. J. Comput.-Aided. Mol.
Des..

[136] Pavlova M., Klvana M., Prokop Z., Chaloupkova R., Banas P., Otyepka M., Wade R. C., Tsuda M., Nagata Y., Damborsky J. (2009). Redesigning Dehalogenase Access Tunnels
as a Strategy for Degrading an Anthropogenic Substrate. Nat. Chem. Biol..

[137] Koudelakova T., Chovancova E., Brezovsky J., Monincova M., Fortova A., Jarkovsky J., Damborsky J. (2011). Substrate Specificity of Haloalkane Dehalogenase. Biochem. J..

[138] Biedermannová L., Prokop Z., Gora A., Chovancová E., Kovács M., Damborský J., Wade R. C. (2012). A Single Mutation
in a Tunnel to the Active Site Changes the Mechanism and Kinetics
of Product Release in Haloalkane Dehalogenase LinB. J. Biol. Chem..

[139] Okai M., Ohtsuka J., Imai L. F., Mase T., Moriuchi R., Tsuda M., Nagata K., Nagata Y., Tanokura M. (2013). Crystal Structure and Site-Directed Mutagenesis Analyses
of Haloalkane Dehalogenase LinB from *Sphingobium* sp. Strain MI1205. J. Bacteriol..

[140] Stepankova V., Khabiri M., Brezovsky J., Pavelka A., Sykora J., Amaro M., Minofar B., Prokop Z., Hof M., Ettrich R. (2013). Expansion
of Access Tunnels and Active-Site Cavities Influence Activity of Haloalkane
Dehalogenases in Organic Cosolvents. ChemBioChem.

[141] Mazur A., Grinkevich P., Chaloupkova R., Havlickova P., Kascakova B., Kuty M., Damborsky J., Kuta Smatanova I., Prudnikova T. (2021). Structural Analysis of the Ancestral
Haloalkane Dehalogenase AncLinB-DmbA. Int. J.
Mol. Sci..

[142] Brezovsky J., Babkova P., Degtjarik O., Fortova A., Gora A., Iermak I., Rezacova P., Dvorak P., Smatanova I. K., Prokop Z. (2016). Engineering
a De Novo Transport Tunnel. ACS Catal..

[143] Raczyńska A., Kapica P., Papaj K., Stańczak A., Shyntum D., Spychalska P., Byczek-Wyrostek A., Góra A. (2023). Transient Binding Sites at the Surface
of Haloalkane
Dehalogenase LinB as Locations for Fine-Tuning Enzymatic Activity. PLoS One.

[144] Verma H., Kaur J., Thakur V., Dhingra G. G., Lal R. (2024). Comprehensive Review on Haloalkane Dehalogenase (LinB): A β-Hexachlorocyclohexane
(HCH) Degrading Enzyme. Arch. Microbiol..

[145] Gelfand N., Orel V., Cui W., Damborský J., Li C., Prokop Z., Xie W. J., Warshel A. (2025). Biochemical and Computational
Characterization of Haloalkane Dehalogenase Variants Designed by Generative
AI: Accelerating the S_N_2 Step. J.
Am. Chem. Soc..

[146] Magdziarz T., Mitusińska K., Gołdowska S., Płuciennik A., Stolarczyk M., Ługowska M., Góra A. (2017). AQUA-DUCT: A Ligands Tracking Tool. Bioinformatics.

[147] Thirunavukarasu A. S., Szleper K., Tanriver G., Marchlewski I., Mitusinska K., Gora A., Brezovsky J. (2025). Water Migration
through Enzyme Tunnels Is Sensitive to the Choice of Explicit Water
Model. J. Chem. Inf. Model..

[148] Oakley A. J., Klvana M., Otyepka M., Nagata Y., Wilce M. C., Damborsky J. (2004). Crystal Structure
of Haloalkane Dehalogenase
LinB from *Sphingomonas paucimobilis* UT26 at 0.95 Å Resolution: Dynamics of Catalytic Residues. Biochemistry.

[149] Chovancová E., Pavelka A., Beneš P., Strnad O., Brezovský J., Kozlíková B., Gora A., Šustr V., Klvaňa M., Medek P. (2012). CAVER 3.0: A Tool for
the Analysis of Transport Pathways
in Dynamic Protein Structures. PLoS Comput.
Biol..

[150] Magdziarz T., Mitusińska K., Bzówka M., Raczyńska A., Stańczak A., Banas M., Bagrowska W., Góra A. (2020). AQUA-DUCT
1.0: Structural and Functional Analysis of
Macromolecules from an Intramolecular Voids Perspective. Bioinformatics.

[151] Naden L. N., Nash J., Crawford T. D., Ringer
McDonald A. (2024). Cookiecutter for Computational Molecular Sciences:
A Best Practices Ready Python Project Generator. J. Chem. Educ..

