# Supplementary material for: WatCon: A Python Tool for Analysis of Conserved Water Networks Across Protein Families
Source: JACS Au. 2025 Nov 20;5(12):6379–95. doi: 10.1021/jacsau.5c00447 (PMC12728608; doi:10.1021/jacsau.5c00447)
Supplement: Supplementary file 1 [file au5c00447_si_001.pdf]

Supporting Information for:

WatCon: A Python Tool for Analysis of Conserved Water Networks Across Protein Families

Alfie-Louise R. Brownless,[1] Travis Harrison-Rawn[1] and Shina C. L. Kamerlin[1-3,*]

1. School of Chemistry and Biochemistry, Georgia Institute of Technology, 901 Atlantic Drive NW, Atlanta, Georgia 30332-0400, USA
2. School of Chemical and Biomolecular Engineering, Georgia Institute of Technology, 311 Ferst Drive, Atlanta, Georgia, 30332-0400, USA
3. Department of Chemistry, Lund University, Box 124, 221 00 Lund, Sweden

Corresponding author email address: skamerlin3@gatech.edu

Table of Contents

Supplementary Methods S2

Processing PDB Files S2

Molecular Dynamics Simulations S2

WatCon Analysis S3

Supplementary Technical Considerations S6

H-bond Definitions S6

Differentiating Between Water Molecules Using WatCon S7

Comparison of Water Hotspots Obtained from Static Structures and Molecular Dynamics Simulations (Example Application 2) S7

Generation of the Non-Receptor PTP Water Network (Example Application 3) S8

Impact of Active Region Sphere Size on Conservation Scores (Example Application 3) S9

Impact of Protein Structure Resolution on Conservation Scores (Example Application 3) S10

WatCon Pitfalls and Caveats S12

Supplementary Figures S15

Supplementary Tables S29

Supplementary References S35

Supplementary Methods

We provide below details of WatCon analysis and additional simulations performed to aid in WatCon analysis of dynamical trajectories, presented in the main text.

Processing PDB Files

For all applications described below, PDB files were obtained from the Protein Data Bank,[1] and processed for simulations and analysis using AmberTools.[2] Required refinement and alignment procedures of structures are described in the WatCon documentation. The MolSSI Cookiecutter was used for development of the Python package.[3]

Molecular Dynamics Simulations

Molecular dynamics simulations used for Application 2 were performed following protocols outlined in previous work.[4] In brief, molecular dynamics simulations were performed using starting structures of PTP1B in unliganded WPD-loop open and closed states obtained from prior work.[4] Simulations were run using GROMACS v. 2022[5] and the AMBER ff14SB forcefield[6] and the TIP3P[7] water model. The system was minimized using the steepest descent algorithm before 100ps of heating to 300K using velocity rescaling. Then, 100ps of additional NPT equilibration utilizing a Parinello–Rahman barostat[8, 9] was conducted at 300K and 1atm, before initiating production using the same settings. For production, we simulated 8 independent replicas of 1.5μs simulation time each for both open and closed WPD-loop structures (16 total replicas to a cumulative simulation time of 24 μs). Simulation convergence is shown in **Figure S13**. The SHAKE[10] algorithm was used to constrain hydrogen bonds, the Particle-Mesh Ewald method[11] was utilized to handle long-range electrostatics, and simulations were conducted utilizing a 2fs timestep. Trajectory snapshots were saved every 250ps for further analysis.

The following starting structures were used for molecular dynamics simulations performed in Application 5: LinB-WT (PDB ID: 1MJ5[12]), LinB-Open (PDB ID: 5LKA[13]) and LinB-Closed (PDB ID: 4WDQ[12]). Structure construction was conducted following the protocols outlined in previous work.[4] Simulations were run using GROMACS v. 2024[5] along with the AMBER ff14SB forcefield[6] and the TIP3P[7] water model. The system was minimized using the steepest descent algorithm before 100ps of heating to 300K using velocity rescaling. Then, 100ps of additional NPT equilibration was performed utilizing a Parinello–Rahman barostat[8, 9] at 300K and 1atm before final production using the same settings. For each structure, three replicas of 300ns simulations were conducted as final production, and snapshots were saved every 25ps for further analysis. Simulation convergence is shown in **Figure S14**.

WatCon Analysis

We provide a variety of user-tunable parameters in the WatCon package, in order to be able to tailor to the needs of specific systems/scientific questions. We provide a summary of the choices made for the example applications in this manuscript, but note that ideal parameter choices can vary dependent on system.

For the trajectory data analyzed in Applications 1 and 2, we created water-protein networks including all hydrogen atoms, defining hydrogen bonds as having a maximum acceptor – water hydrogen (A…H) distance of 2.7Å (corresponding to a maximum O-O distance of 3.7Å) and a minimum water oxygen (donor) – water hydrogen - acceptor (D-H…A) angle of 120 degrees. We define our active region by the center of mass between residues Arg221 and Cys215 and calculate water networks for the active region only in order to save computational cost.

For the PTP1B wild-type crystal structures analyzed in Application 2 and Application 3, we utilized all available wild-type PDB structures of PTP1B, due to the high number of available

structures of wild-type and mutant forms of this well-studied protein (>300). In order to ensure that we studied only wild-type sequences, we first collected all structures presented on the Uniprot page[14] for human PTPN1 (PTP1B). We then removed any structures that were associated directly with mutants (*via* information in the PDB description). We then performed a multiple sequence alignment to ensure that the remaining structures were of the wild-type protein (using the sequence identified as PTPN1 on Uniprot[14] as a reference) and not an unlabeled mutant. This reduced our dataset from 402 structures to 306.

We do, however, note that some amino acid modifications are necessary in order to properly crystallize a given protein. These include but are not limited to, notable modifications at the N- and C-terminal regions of the protein and direct modifications of the catalytic cysteine residue (in particular for PTPs). As a result, we kept in our dataset any structures which noticeably had these types of modifications. Further, we noticed many structures (on the order of 100s) contained particular modifications C92V or C32S. Since these modifications were overwhelmingly common (and clustered water positions did not vary substantially with or without these structures included in our dataset), we decided to keep these structures in order to ensure the highest sample size possible. We then created water-protein networks that did not include hydrogen atoms, and thus we defined hydrogen bonds as having a maximum Donor - Acceptor (D - A) distance of 3.7Å. We also included a custom residue type (parameters provided in the Zenodo data package, DOI: 10.5281/zenodo.15213225) to represent the phosphorylated cysteine residues involved in the PTP-catalyzed reaction for our molecular dynamics simulations (**Figure S3**). When classifying water angles for WatCon calculations as described in **Figure 2**, we utilized the reference residue coordinates of the C_α-atoms of L71 and T154 (PTP1B numbering), to ensure

consistency across MD trajectories and crystal structures, despite different coordinate alignments.

When clustering coordinates of static structures for Application 2, we performed clustering using the hdbscan algorithm,[15] selecting a minimum of 10 samples per cluster and an ε value of 0.0, In all cases, we use the 'default' water name, which includes water residues labeled as WAT, HOH, H2O, and SOL. When using WatCon to calculate conservation scores, we consistently used a distance cutoff of 1Å to classify a water as conserved, and a radius of 6Å around each water to calculate the local density.

For Application 4, we used the hdbscan[15] for clustering with a minimum of 10 samples per cluster and an ε of 0.0. We used a distance cutoff of 1.0 Å when calculating cluster conservation scores. Finally, when analyzing the impact of engineered tunnels on the solvation of the LinB active site in Application 5, we created water-protein networks including all hydrogen atoms, defining hydrogen bonds as having a maximum acceptor – water hydrogen (A…H) distance of 2.0Å (corresponding to a maximum O-O distance of 3.0Å) and a minimum water oxygen (donor) – water hydrogen - acceptor (D-H…A) angle of 120 degrees. In this case, we defined our active region by a sphere of 14Å centered on the D108 residue.

Supplementary Technical Considerations

In this section, we will provide further detailed information on both technical considerations that have gone into the construction and design of WatCon, as well as additional pitfalls and caveats that users should consider when setting up and executing WatCon analysis.

H-bond Definitions

As outlined in the main text, when constructing water networks, WatCon by default uses a 3.8Å distance cutoff on the donor-acceptor distance. Furthermore, in structures where protons are present (for instance simulation trajectories or structures obtained from neutron scattering experiments), an additional 150° donor-hydrogen-acceptor angle cutoff is introduced to define hydrogen bonds. Both these cutoffs are user-adjustable and can be adapted to system as needed. We note that these cutoffs are slightly higher than the values presented in ref. [16], for a number of reasons. Firstly, as pointed out in the main text, having a slightly looser definition of hydrogen bonding criteria as a default allows WatCon to identify prospective hydrogen bonds also in lower-resolution structures. Following from this, this more flexible cut-off also takes into account both the impact of local environment on the strengths of water hydrogen bonds,[17] as well as variations in water-protein H-bond geometries caused by the identity of the protein side chain.[18] For instance, as pointed out in ref. [18], the sulfur atoms in Met and Cys side chains are capable of forming H-bonds with water, both as H-bond donors and as acceptors, but with relatively long and weak geometries (maximum H-bond lengths of 3.6Å and H-bond angles of 104° ± 30°).[19] Importantly, however, these are not hard-coded cutoffs and can be modified as desired by the user.

Differentiating Between Water Molecules Using WatCon

To differentiate between water molecules, WatCon calculates water positions *via* measuring two angles, based on defining two reference points (**Figure 2**). This allows distinctly different water molecules to be differentiated between using only two dimensions, while also taking into account differences in sequence structure. The reference points used to calculate the angles are flexible, can be chosen to be static coordinate values, or relative C_α-atom coordinates. If using relative atom coordinates, it is important that the user chooses atom positions which are rigid across all structures analyzed. The flexibility of the relative atom coordinate options allow for comparison of water positions across different structures and trajectories, without the need for structural alignment. The distribution of these two angles can then be projected onto a 2-D plot, in order to characterize the network, as discussed in the main text.

Comparison of Water Hotspots Obtained from Static Structures and Molecular Dynamics Simulations (Example Application 2)

A technical challenge when comparing water networks generated from static structures *vs.* molecular dynamics trajectories is the fact that the dynamical trajectories contain substantively more water molecules than the crystallographic structures. As a result, it is non-trivial to directly compare water hotspots between the two data sets when performing separated analysis. On the one hand, an accurate (but computationally expensive) clustering method such as hdbscan[15] will provide an incredibly large number of clusters for the dynamical trajectories, which makes subsequent analysis of the most stable water locations more challenging. However, if the required number of samples per cluster are increased to reduce the overall number of clusters, this in turn causes significant problems with memory allocations, as even a truncated active subregion can require increasingly large memory allocations to perform the clustering. In contrast, the

experimental data sets are often too sparse to create meaningful histograms of the densities, making it difficult to compare the two data sets with the same analysis approach (note that this is not an issue when combining the data sets, as would be expected in routine usage of WatCon).

To address this issue, we have coupled the two approaches using hdbscan[15] to perform clustering on our static structures, whereas we have constructed density histograms followed by calculation of highest occupancy coordinates when analyzing the dynamical data. While not ideal, this allows for a consistent method to obtain a useful number of clusters for both the static and dynamical data, and results in positions of clusters that are reflective of the true locations of the water hotspots. Therefore, in order to analyze positions of water hotpots in both data sets, we first performed hdbscan clustering,[15] with a criteria of minimum 5 cluster samples on all available PTP1B crystal structures in the WPD-loop closed conformation (**Table S1**). Based on this, we then used WatCon to interface with MDAnalysis[20] to compute a density analysis for the dynamic structures, calculate locations of density maxima, and compare these coordinates to the clusters determined for the static structures (**Figure 5F**).

Generation of the Non-Receptor PTP Water Network (Example Application 3)

A challenge in selecting structures for generating our water network across non-receptor PTPs using experimental structures is that there exists significant variation in the number of crystal structures available across all non-receptor PTPs, ranging from over 200 for PTPN1 (PTP1B) to only two in PTPN12. For better direct comparison, we chose only one structure of each PTP in each conformation of the WPD-loop (**Table S2**) for our analyses. This avoids biasing of the distribution of water locations by, for example, introducing hundreds of structures of PTPN1 while only including two structures of PTPN12. Furthermore, we focused exclusively on wild-type PTPs, with the exception of structures with C->S substitution at the catalytic cysteine or a

D->X substitution at the general acid, which are often made in order to resolve a structure at the WPD-loop closed configuration.[21, 22]

We note, conversely, that not all non-receptor PTP genes contain resolved crystal structures, and many which do only contain one WPD-loop conformation, resulting in a subset of 24 crystal structures of PTPs with their WPD-loops in both conformations, 13 of which are in the WPD-loop open conformation and 11 in the WPD-loop closed conformation (**Table S2**). Further, almost all of the crystal structures contain either a ligand (in the case of PDB IDs: 2F71,[23] 4GE6[24]), phosphate ion (in the case of PDB IDs: 1WCH,[25] 1ZC0,[26] 3BRH,[1] 4GRZ,[21] 5HDE,[1] 8GVV,[27] 2I75,[22] 8SLS[28]), or other solvent molecule (in the case of PDB ID: 3O4U[29]). As a result, a complete WPD-loop closed water network of these PTPs in their unliganded states cannot be generated from these structures alone. Since limitations such as these are inherent to crystal structure analysis (due to the often high difficulty of resolving structures in desired conformations), we choose to include these structures in our analysis. We also note, however, that further simulation of structures in desired conformational states can greatly aid in determining full water networks, especially for static structures which contain added ligands, ions, or other solvent.

Impact of Active Region Sphere Size on Conservation Scores (Example Application 3)

As it is possible that the active region sphere size can impact calculated conservation scores, we have tested the impact of modulating active region sphere size against protein structure resolution (**Figures 6** and **S8**) on the calculated conservation scores. In doing so, we observe a correlation between conservation score and resolution of the structure: for instance, in the case of PTPN14 (1.8Å resolution structure), conservation scores tend to be high using a 1Å cutoff, and much lower using a 10Å cutoff. Since this structure has a very high resolution, there are numerous

resolved water molecules, as opposed to the lower resolution structures available. As a result, it is statistically more likely that the water molecules that are present will align with our conserved clusters. Therefore, we suggest the user to increase the density cutoff radius to mitigate impacts of resolution-based conservation score inflation if this type of effect is undesired. In contrast, in the case of PTPN2 (2.5Å structure), which has hardly any resolved water molecules in the structure, conservation scores dramatically increase as the density radius increases. Thus, it's important to find a sweet spot, depending on the quality of the structures available to the user for analysis. In particular, the user should be careful to not use very large sphere sizes with low resolution structures, as this will artificially inflate the conservation scores compared to high resolution structures, providing potentially misleading results.

Impact of Protein Structure Resolution on Conservation Scores (Example Application 3)

As shown in the prior section, the resolution of the crystal structures used in WatCon analysis of static structures can impact calculated conservation scores. In the case of the PTPs used for this Application (**Table S2**), structures range from 1.37Å (PTPN6, 4GRZ[21]) to 2.5Å (PTPN22, 3OLR[1]) resolution, with 18/23 structures having a resolution of 2.0A or less. Detailed analysis of the ratio of expected water molecules to the number of amino-acid residues in the Protein Data Bank[1] has shown that this ratio exceeds 1.5 in atomic resolution structures (1.2Å or higher[30]), but drops to 0.25 at structures of around 2.5Å, with 800 protein crystal structures determined at resolutions > 2.5Å lacking any water molecules at all.[31] It was pointed out that the typical hydrogen-bonding distances of water molecules in the first hydration layer are ~2.8Å,[32] so one would expect at least some water molecules to be clearly discernible at map resolutions of 2.75Å or better.[31] It was thus argued that the absence of water molecules in structural data that was determined at resolutions ~2Å or greater likely indicated problems with (possibly a combination

of) the diffraction data, the refinement process, or the deposition process.[31] This clearly suggests caution when working with static structures with resolutions of 2Å or greater to determine water maps.

To test the impact of this issue on our calculations, we re-calculated the conservation scores presented in **Figure 6** using only a subset of the structures presented in **Table S2** *(i.e.*, all 18 structures with resolutions 2.0Å or less) for comparison, with this data presented in **Figure S9**. We observe that, the clustered water hotspot positions (**Figure S9B**) remain similar to those determined from the large collection of WPD-loop closed structures analyzed in **Figure 6**. This is likely because the water molecules resolved in the low-resolution crystal structures are highly stable (otherwise they would not have been resolved at low resolution), and so would almost certainly align with the hotspots obtained from higher resolution structures. We then classified the conservation of each of the high-resolution non-receptor PTP structures to this set of water clusters, and note that when removing lower resolution structures, it is clearer that PTPN1 (PTP1B) closer WPD-loop structures share the highest similarity with the closed cluster hotspots. In this way, removing low resolution crystal structures can serve to filter out some noise that can occur from limited data, provided there is sufficient structural data available across the protein family to still have a meaningfully sized data set after filtering (as is the case in **Application 4**). Overall, both issues with missing residues and structural resolution have the potential to pose challenges when studying water maps using only experimentally derived structures across full protein families (where structural quality can be highly variable), and we therefore recommend supplementing this analysis with molecular simulations of fully solvated systems to obtain "complete" water networks, where computational resources allow.

WatCon Pitfalls and Caveats

We have developed WatCon as a powerful tool to track the position of conserved water hotspots across protein variants and protein families, both to provide fundamental biochemical insight into the role of water in specific systems, and also as a tool to guide drug discovery and protein design. We have showcased several example applications of the ways in which WatCon can be used to achieve this goal. Here, we outline important pitfalls and caveats to take into account when setting up WatCon analysis, that are important for users to be mindful of. In particular, it is necessary to remember that WatCon is a water *tracking* tool, not a water *placement* tool (several excellent tools exist in both categories,[33-49] although these focus on individual proteins or trajectories and not the large-scale comparative analysis WatCon can provide). This means that the quality of WatCon results is directly related to the quality of the underlying data, and there are several factors that will, in turn, impact the quality of the underlying data.

The first, and most obvious of these, is the quality of the structural information used. In **Example Application 2**, we provide a comparison of results obtained from analysis of static structures, *vs.* analysis of molecular simulations. When designing WatCon, we believe it to be important for WatCon to have the capacity to perform analysis using static experimental structures and not only simulation trajectories, for the simple reason that performing extensive molecular simulations requires substantive computational resources, which will not be available to all users (note that, as outlined in **Figure 1**, WatCon can also combine results from static and dynamic analysis, and therefore one can also focus more extensive sampling on a subset of family members while supplementing with static structures for the rest of the family).

There are several issues to take into account when performing analysis using only static experimental structures, the most important of which is that even at high resolution, such

structures will contain incomplete water networks, due to the fact that it is very challenging to resolve water molecules in crystal structures. For this reason, crystal structures typically contain only tightly bound water molecules,[50] missing more mobile waters (assuming all structural refinement was performed correctly). Further, as discussed in **Application 3**, lower resolution structures (2.5Å or greater, possibly even 2.0-2.5Å) can miss key water molecules due to any combination of problems with the diffraction data, the refinement process, or the deposition process.[31] The lower the resolution of the structures used, the more incomplete the water network, and the more important it becomes to supplement static structure analysis with system solvation and MD analysis, if computational resources permit. Similarly, missing residues in crystal structures will lead to potentially missed interactions with side chains that might be important, and as we illustrated in Example Application 3, the quality of structural data available across a protein family can vary greatly depending on how much interest there has been individual family members, further limiting analysis possible using static structures alone. This is not to say that analysis of static structures by themselves is not useful, but that it is important to bear in mind that such network analysis may be incomplete based on the quality of the structural data available.

As shown in **Example Application 2**, one work around to this is to (further) solvate experimental structures and subject them to rigorous MD equilibration, to capture more complete water networks (this can also be done when performing analysis based on predicted structures using *e.g.*, AlphaFold,[51] which are not solvated). When doing so, we strongly recommend retaining information available on crystallographic water molecules, where available, as it is not guaranteed that the position of crystallographic water molecules will be recovered when performing unrestrained MD simulations,[52, 53] an issue which can also be highly relevant if performing simulations of enzymes in organic solvent.[54] However, even with extensive

equilibration, results will be dependent on force fields, and on water models, and the question of how best to solvate a system and perform such simulations is a challenging question that has been addressed in detail elsewhere (*e.g.*, refs. [52-59], among others). It is always important for the underlying simulations to be properly validated, using best practices in the field.

We emphasize that these pitfalls and caveats are not unique to WatCon, and will impact any water tracking tool, as these will only be as good as the input information provided to them, and despite significant progress, there is not yet a perfect water prediction tool, although, as pointed out in ref. [52], advances in room temperature crystallography, neutron diffraction, cryoEM and crystalline MD simulations will all contribute to both significantly improving the quality of structures available and simulations of biomolecular solvation more broadly. This being said, provided the user is careful in their choice of input structural information, and ensures that any simulations are performed as rigorously as possible, WatCon provides a valuable tool that has both standard features as a water tracker for individual simulation trajectories, and can be extended to more advanced analysis across large ensembles of structures and simulations to provide important biochemical data and insights.

Supplementary Figures

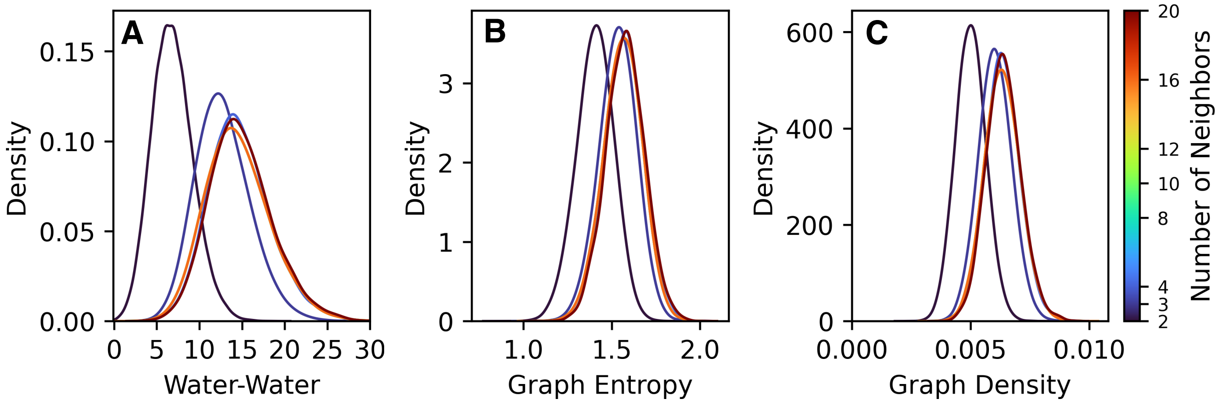

Figure S1. Kernel density estimate (KDE) plots of (**A**) water-water interactions, (**B**) graph entropy, and (**C**) graph density values as the maximum number of neighbors is varied within the k-d tree algorithm (using neighbor cutoffs of 2, 3, 4, 8, 10, 12, 16 and 20 neighbors). The active region was defined as a sphere of 9Å around the center of mass between residues 215 and 221. Values were collected for each frame of 8 x 1.5μs trajectories of PTP1B with an open WPD-loop conformation. This data shows that as the neighbor cutoff increases, (**A**) the total number of water-water interactions calculated within the active region also increases, converging at around 15 interactions in total. Similarly, the (**B**) graph entropy and (**C**) density also increase as this neighbor cut-off increases, and also appear to converge nicely. Importantly, while smaller neighbor cutoffs (2 or 3) exhibit notably different distribution centers compared to other choices tested, cutoffs greater than 8 neighbors produce very similar behavior, making 10 neighbors a reasonable cutoff compromise. However, WatCon users can increase or decrease this cutoff as dictated by the needs of their specific system, or to save computational cost.

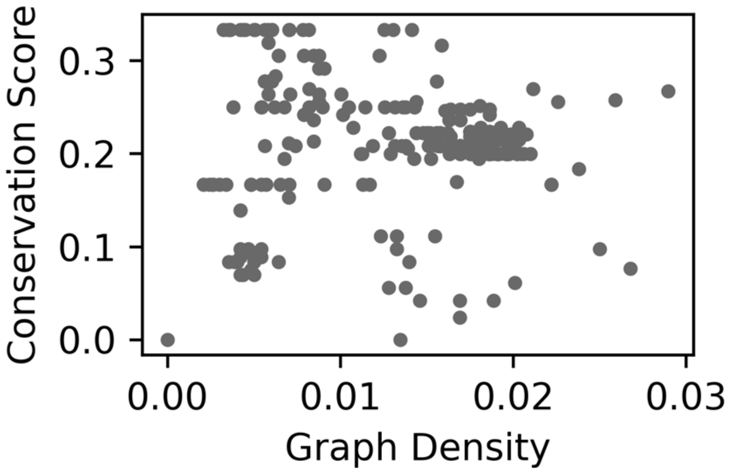

Figure S2. Graph density *vs*. conservation scores with respect to PTP1B WPD-loop closed water coordinates, based on clustering of all water positions in the crystal structures presented in **Table S1**. Clustering was performed using hdbscan[15] (min_samples=10) over all PTP1B WPD-loop closed crystal structures presented. Conserved water molecules were defined as those which lay within 1.0Å of a cluster centroid, and were normalized by the local number of waters around a 6Å sphere centered around the conserved water.

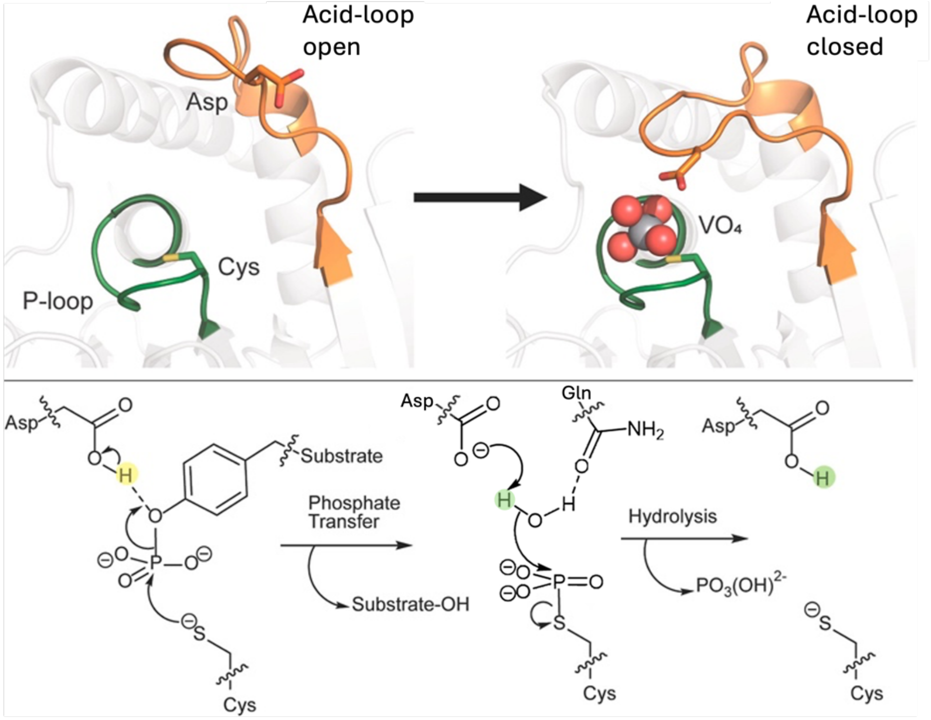

Figure S3. (Top) Visualization of the WPD-loop (acid-loop) open conformations in PTPs. (Bottom) Generalized two-step PTP mechanism. From Ref. [60], published by the American Chemical Society, under a CC-BY license. Copyright © 2024 The Authors.

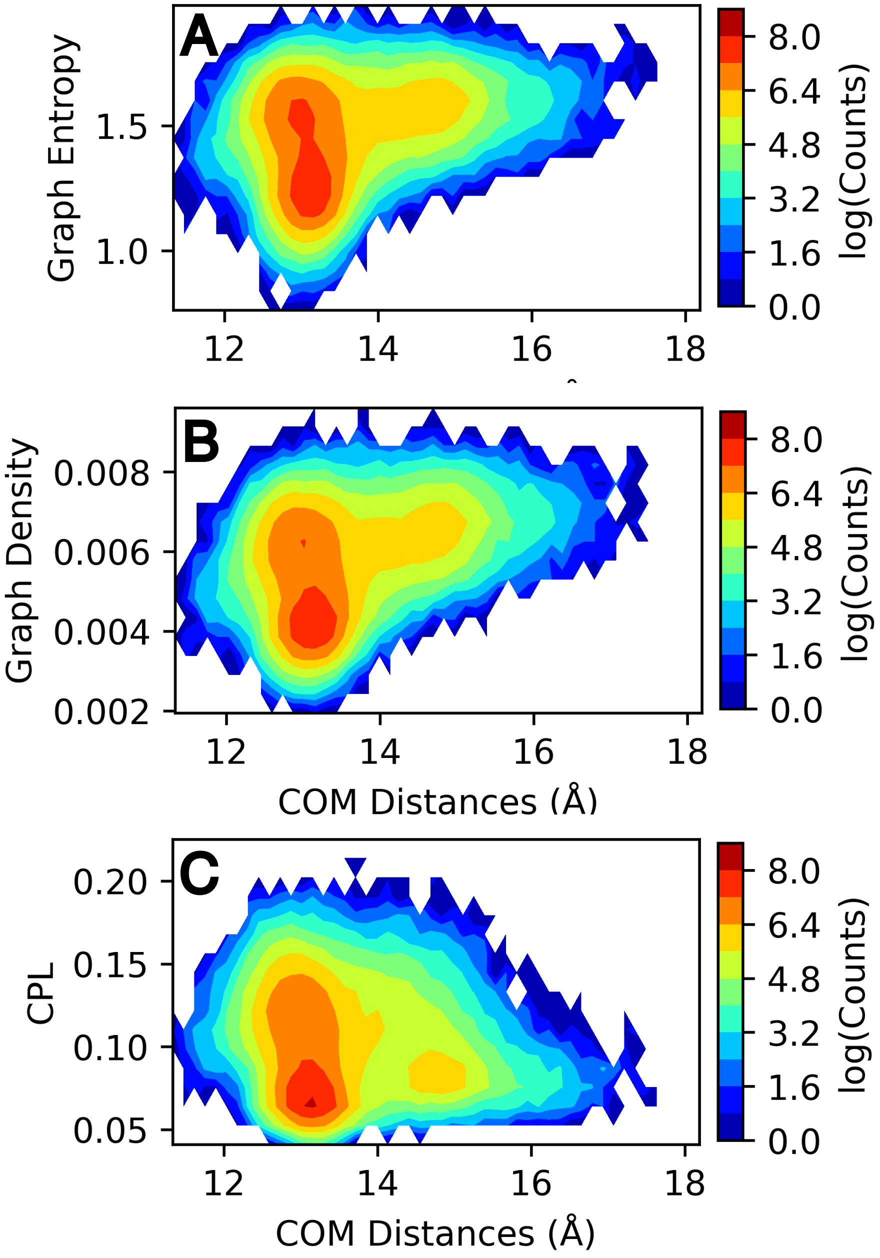

Figure S4. Comparison of center of mass (COM) distances (Å) between the WPD- and P-loops of PTP1B (**Figure S3**), and the calculated (**A**) graph entropy, (**B**) graph density, and (**C**) characteristic path lengths, calculated from 8 x 1.5μs simulations of PTP1B, initiated from each of the WPD-loop closed and open positions (16 trajectories in total).

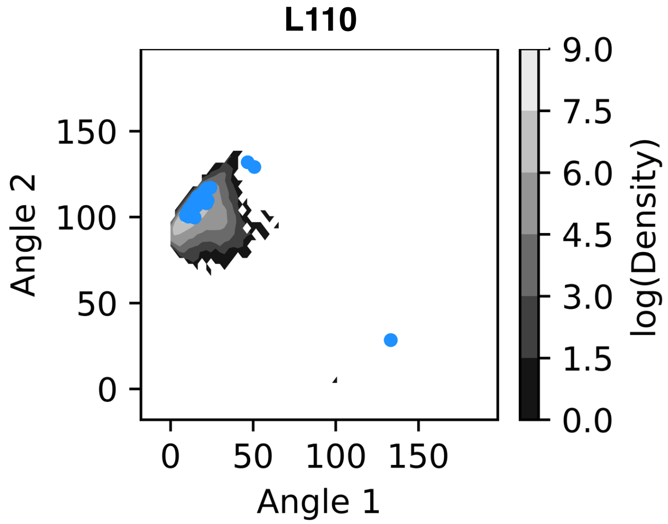

Figure S5. Projection of water classification angles computed for residue L110 (PTP1B numbering). The gray background surface shows angles sampled across 8 x 1.5µs molecular dynamics simulations of PTP1B initiated form the WPD-loop closed conformation. The blue solid circles show angles calculated from the static structures interacting with backbone atoms. The reference points used for the two angle calculations were the C_α-atoms of residues L71 and T154. Angle 1 was calculated as the angle between the closest interacting protein atom of residue L110, the water oxygen, and the C_α-atom of residue L71. Angle 2 was calculated as the angle between the interacting protein atom of residue 115, the water oxygen, and the C $_\alpha$-atom of residue T154.

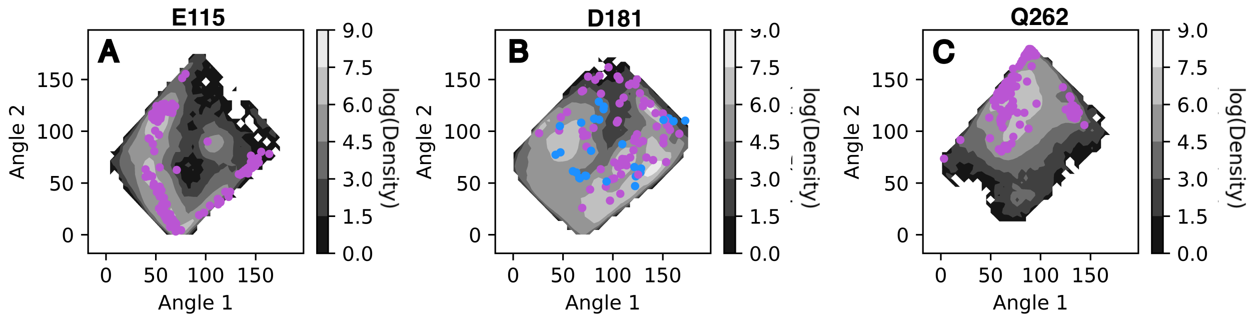

Figure S6. Comparison of water classification angles computed for (**A**) E115, (**B**) D181, and (**C**) Q262, using PTP1B numbering. Note that only side chains are shown here, as only the side chains form interactions with water molecules. Numbers refer to residue positions in the MSA. The gray background surface shows angles sampled across 8 x 1.5μs molecular dynamics simulations of PTP1B initiated form the WPD-loop closed conformation. The solid circles projected onto the surfaces show angles calculated from the static structures, with backbone interactions colored blue and sidechain interactions colored purple. The reference points used for the two angle calculations were the C_{α}-atoms of residues L71 and T154, at the first simulation frame and at one of our static structures, as arbitrarily chosen but consistent reference values for the two data sets (note that here, consistency is more important than the exact reference, in order to avoid any potential alignment issues that can impact the analysis). Angle 1 was calculated as the angle between the closest interacting protein atom of each residue, the water oxygen, and the C_{α}-atom of residue L71. Angle 2 was calculated as the angle between the interacting protein atom, the water oxygen, and the C_{α}-atom of residue T154.

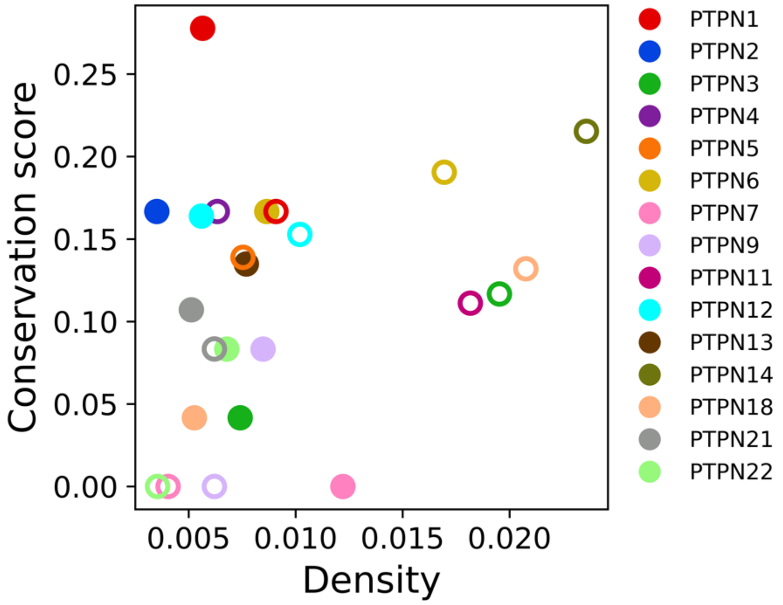

Figure S7. Graph density versus conservation score in relation to closed PTP1B crystal structures for the active site only for a set of non-receptor PTPs. Open circles correspond to structures with open WPD-loops, and closed circles correspond to structures with closed WPD-loops. Conserved water molecules were defined as those which lay within 1.0Å of a cluster centroid and were normalized by the local number of waters around a 6Å sphere centered around the conserved water molecule.

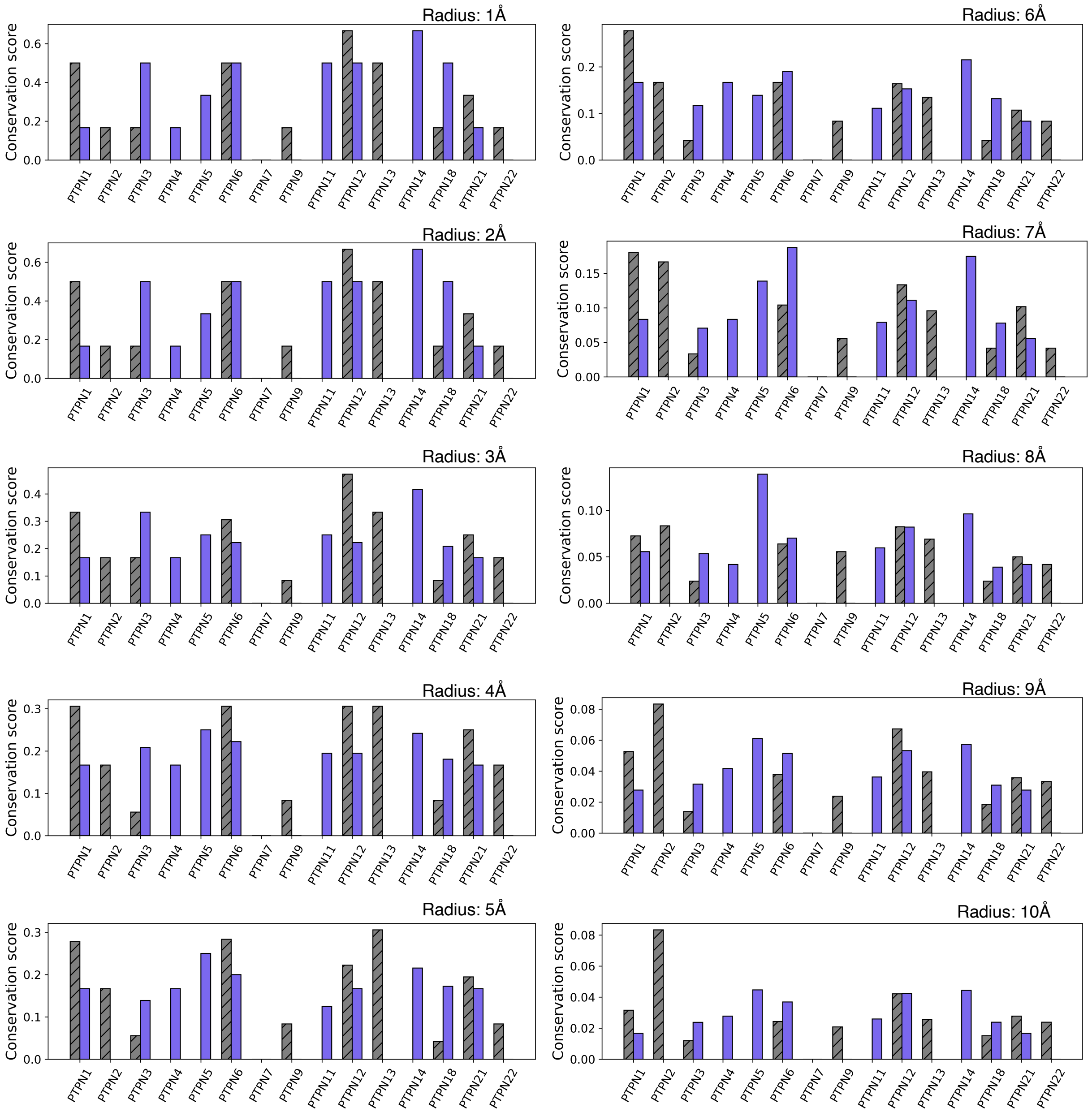

Figure S8. Comparison of conservation scores to clustered WPD-loop closed PTP structures from **Table S2**, depending on the active region radius used to calculate local waters. Water molecules were counted as 'conserved' if they lie within 1Å of a clustered water hotspot.

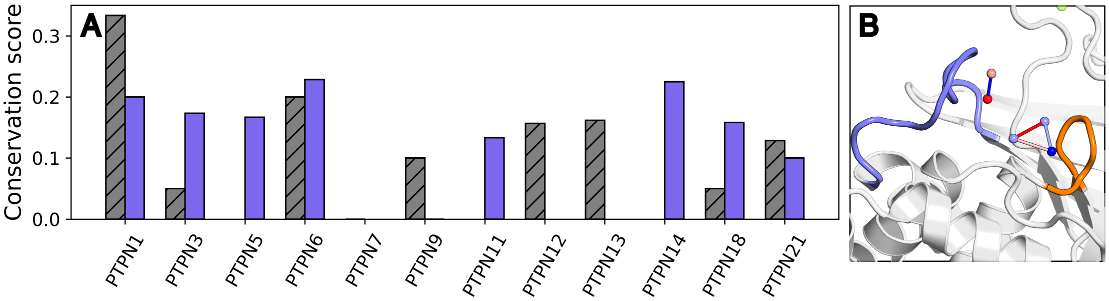

Figure S9. (**A**) Conservation score to PTP1B closed crystal structure summary network calculated for a set of high resolution (≤2Å) non-receptor PTPs using active region waters only. Purple bars correspond to structures with open WPD-loops, and gray hashed bars correspond to structures with closed WPD-loops. (**B**) Projection of water clusters identified for high resolution PTP1B closed crystal structures colored by conservation of particular water positions and by neighbor conservation (blue = less conserved, red = more conserved). The WPD-loop is colored blue, and the P-loop is colored orange.

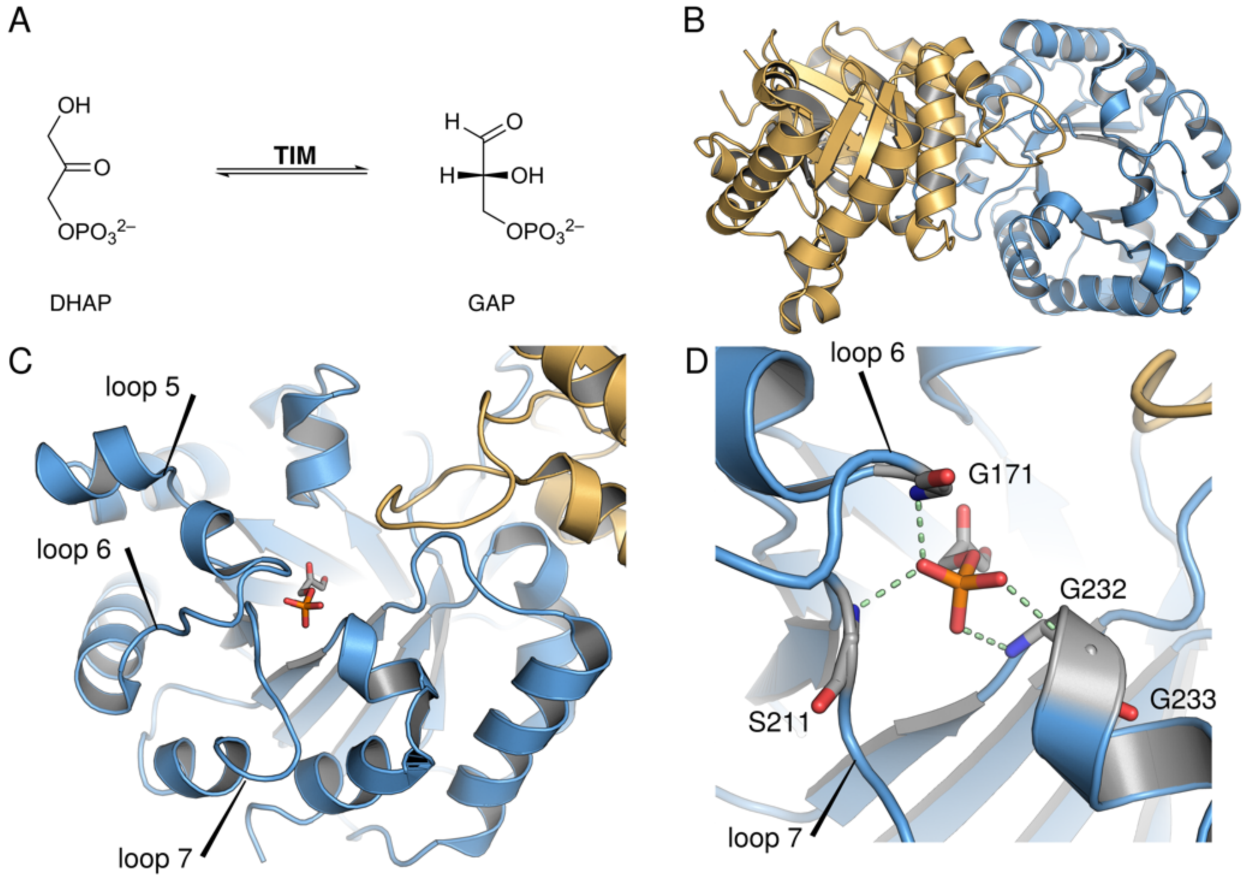

Figure S10. Overview of the (**A**) catalytic mechanism, (**B**) overall dimeric structure, (**C**) catalytic loops and (**D**) substrate stabilizing interactions in the triosephosphate isomerase (TPI) from *Saccharomyces cerevisiae* (*y*TPI, PDB ID: 1NEY[61]). Specifically, in panel **D**, interactions between TPI and the non-reactive phosphodianion group of the substrate play an important role in closure of loop 6 over the active site.[62] From Ref. [63], published by the American Chemical Society, under a CC-BY license. Copyright © 2018 The Authors.

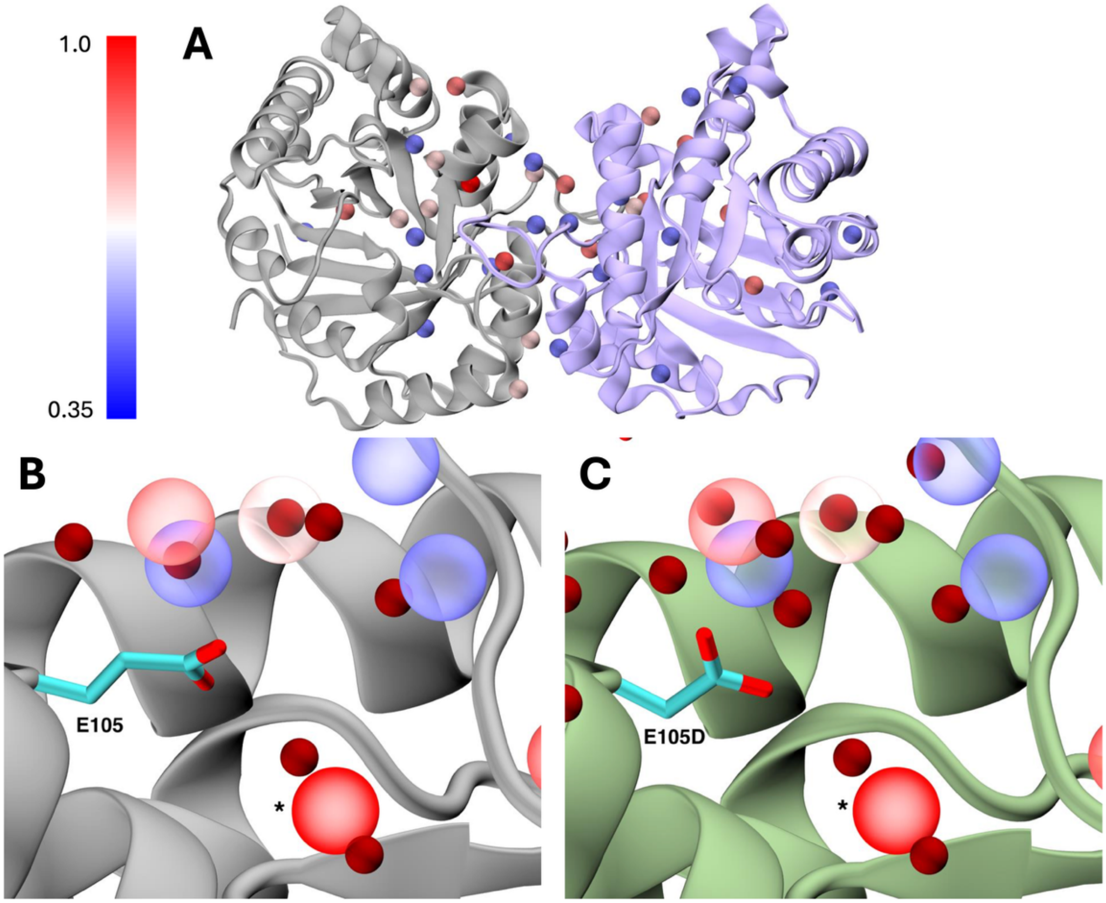

Figure S11. WatCon static structure analysis of the subset of TPI structures shown in **Table S3** that contain the full biological assembly. Shown here are the structures of: (**A**) The human TPI (PDB ID: 2JK2[64]) colored by chain. Conserved clusters are shown as spheres colored by conservation score. (**B**) A close-up of wild-type human TPI (PDB: 2JK2[64]) in grey, with residue E105 shown in licorice. Water clusters are shown as transparent spheres colored by conservation. Structural waters are shown as dark red spheres. The highest conserved cluster (conservation score = 1.00) is highlighted with an asterisk. (**C**) A close-up of E105D human TPI (PDB: 2VOM[64]) in green, aligned to water clusters from the analyzed dimers. Even though we analyzed different oligomerization states compared to data shown in **Figure 8**, the highest conserved cluster is still adjacent to residue E105. This use case showcases WatCon's consistent ability to identify functional regions critical for structural water regulation despite using varied input strategies.

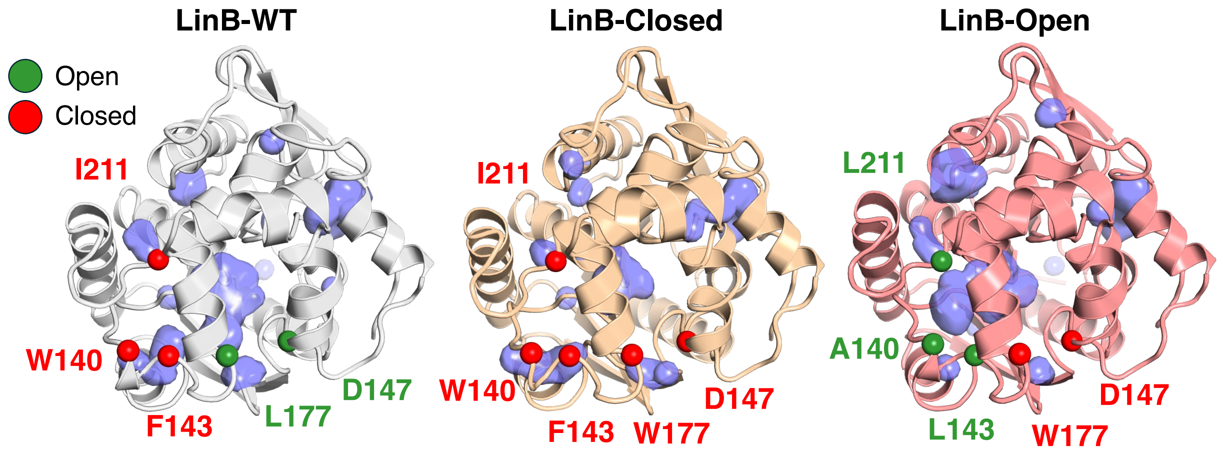

Figure S12. Comparison of open tunnel cavities for LinB-WT, LinB-Closed, and LinB-Open structures (PDB IDs: 1MJ5[12], 4WDQ[12] and 5LKA[13], respectively). Red spheres indicate residues which prevent a tunnel from being open (thus the tunnel is closed), and green spheres indicate residues which allow a tunnel to be open. Blue surfaces indicate internal cavities.

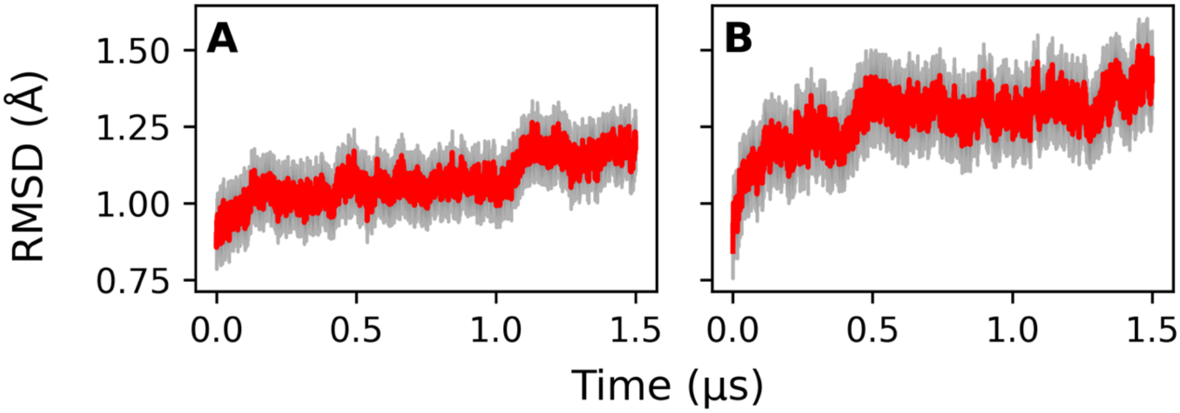

Figure S13. Root mean squared deviations (RMSD (Å)) of all C_α atoms for PTP1B initiated from unliganded WPD-loop (**A**) closed and (**B**) open PTP1B structures. Red lines indicate averages over 8 x 1.5μs replicas, and gray shading indicates corresponding standard deviations. Due to the high flexibility of the N-terminal α1-helix in the PTP1B structure, this region was excluded from the above calculations.

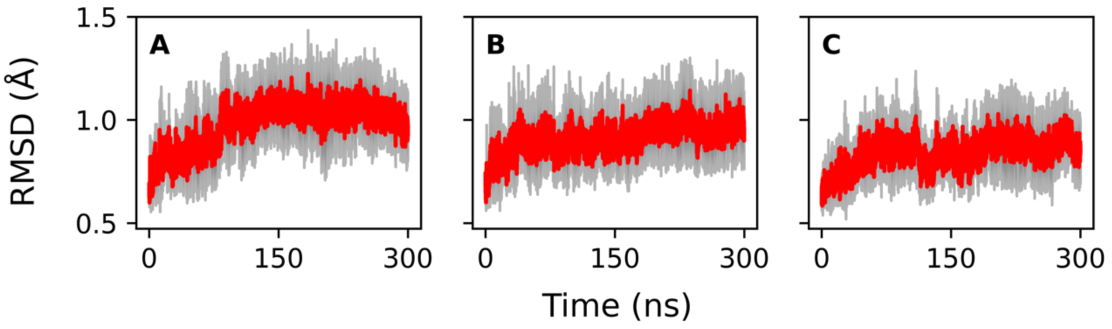

Figure S14. Root mean squared deviations (RMSD (Å)) of all C_α atoms for **A)** LinB-WT, **B)** LinB-Open and **C)** LinB-Closed structures. Red lines indicate averages over 3 x 300ns replicas, and gray shading indicates corresponding standard deviations. Due to high flexibility of the first 10 residues of the structure, RMSD calculations were performed excluding this region.

Supplementary Tables

Table S1. Crystal structures of PTP1B used for analysis in this work (PDB IDs).[a]

PTP1B Structures	WPD-loop Conformation
1JF7,[65] 1NL9,[66] 1NNY,[66] 1NO6,[66] 1NZ7,[67] 1OEM,[68] 1OES,[69] 1OET,[69] 1OEU,[69] 1OEV,[69] 1ONY,[70] 1ONZ,[70] 1PH0,[71] 1PYN,[72] 1T48,[73] 1T49,[73] 1XBO,[74] 2CM2,[75] 2CM3,[75] 3A5J,[1] 3A5K,[1] 3D9C,[76] 3EAX,[77] 3EB1,[77] 3EU0,[78] 3SME,[79] 4I8N,[80] 4QBW,[81] 5K9V,[82] 5KA0,[82] 5QDE,[83] 5QDF,[83] 5QDG,[83] 5QDH,[83] 5QDI,[83] 5QDJ,[83] 5QDK,[83] 5QDL,[83] 5QDM,[83] 5QDN,[83] 5QDO,[83] 5QDP,[83] 5QDQ,[83] 5QDR,[83] 5QDS,[83] 5QDT,[83] 5QDU,[83] 5QDV,[83] 5QDW,[83] 5QDX,[83] 5QDY,[83] 5QDZ,[83] 5QE0,[83] 5QE1,[83] 5QE2,[83] 5QE3,[83] 5QE4,[83] 5QE5,[83] 5QE6,[83] 5QE7,[83] 5QE8,[83] 5QE9,[83] 5QEA,[83] 5QEB,[83] 5QEC,[83] 5QED,[83] 5QEE,[83] 5QEF,[83] 5QEG,[83] 5QEH,[83] 5QEI,[83] 5QEJ,[83] 5QEK,[83] 5QEL,[83] 5QEM,[83] 5QEN,[83] 5QEO,[83] 5QEP,[83] 5QEQ,[83] 5QER,[83] 5QES,[83] 5QET,[83] 5QEU,[83] 5QEV,[83] 5QEW,[83] 5QEX,[83] 5QEY,[83] 5QEZ,[83] 5QF0,[83] 5QF1,[83] 5QF2,[83] 5QF3,[83] 5QF4,[83] 5QF5,[83] 5QF6,[83] 5QF7,[83] 5QF8,[83] 5QF9,[83] 5QFA,[83] 5QFB,[83] 5QFC,[83] 5QFD,[83] 5QFE,[83] 5QFF,[83] 5QFG,[83] 5QFH,[83] 5QFI,[83] 5QFJ,[83] 5QFK,[83] 5QFL,[83] 5QFM,[83] 5QFN,[83] 5QFO,[83] 5QFP,[83] 5QFQ,[83] 5QFR,[83] 5QFS,[83] 5QFT,[83] 5QFU,[83] 5QFV,[83] 5QFW,[83] 5QFX,[83] 5QFY,[83] 5QFZ,[83] 5QG0,[83] 5QG1,[83] 5QG2,[83] 5QG3,[83] 5QG4,[83] 5QG5,[83] 5QG6,[83] 5QG7,[83] 5QG8,[83] 5QG9,[83] 5QGB,[83] 5QGC,[83] 5QGD,[83] 5QGE,[83] 5QGF,[83] 6B8Z,[83] 6W30,[84] 7FQM,[85] 7FQN,[85] 7FQO,[85] 7FQP,[85] 7FQQ,[85] 7FQR,[85] 7FQS,[85] 7FQT,[85] 7FQV,[85] 7FQW,[85] 7FQX,[85] 7FQY,[85] 7FQZ,[85] 7FRE,[85] 7FRF,[85] 7FRG,[85] 7FRH,[85] 7FRI,[85] 7FRJ,[85] 7FRK,[85] 7FRL,[85] 7FRM,[85] 7FRN,[85] 7FRO,[85] 7FRP,[85] 7FRQ,[85] 7FRS,[85] 7FRT,[85] 7FRU,[85] 7GS7,[86] 7GS8,[86] 7GS9,[86] 7GSC,[86] 7GSD,[86] 7GSE,[86] 7GSF,[86] 7GSG,[86] 7GSH,[86] 7GSJ,[86] 7GSK,[86] 7GSN,[86] 7GSQ,[86] 7GSU,[86] 7GSX,[86] 7GSZ,[86] 7GT2,[86] 7GT8,[86] 7GTB,[86] 7GTC,[86] 7GTF,[86] 7GTJ,[86] 7GTQ,[86]	Open

7GTR,[86] 7GTW,[1] 7GTX,[1] 7GTY,[1] 7GTZ,[1] 7GU0,[1] 7GU1,[1] 7GU2,[1] 7GU3,[1] 7GU4,[1] 7GU5,[1] 7GU6,[1] 7GU7,[1] 7GU8,[1] 7GU9,[1] 7GUA,[1] 7GUB,[1] 7GUC,[1] 7KEY,[87] 7KLX,[87] 7LFO,[84] 8DU7,[88] 8G65,[89] 8G67,[89] 8G68,[89] 8G69,[89] 8G6A,[89] 8U1E[90], 9CYO,[91]	
1AAX,[92] 1BZJ,[93] 1EEN,[94] 1EEO,[94] 1G7G,[95], 1KAK,[96] 1KAV,[97] 1LQF,[98], 1OEO,[68] 1PTU,[99]1PTY,[92] 1PXH,[100] 1Q1M,[101] 1Q6J,[102] 1Q6M,[102] 1Q6N, 1Q6P,[102] 1Q6S,[102] 1Q6T,[102] 1QXK,[103] 1SUG,[104] 1WAX,[105] 2AZR,[106] 2B07,[106] 2BGD,[107] 2BGE,[108] 2CM7,[75] 2CM8,[75] 2CMA,[75] 2CMB,[75] 2CMC,[75] 2CNE,[109] 2CNF,[109] 2CNG,[109] 2CNH, 2CNI,[109] 2F6T,[23] 2F6V,[23] 2F6W,[23] 2F6Y,[23] 2F6Z,[23] 2F70,[23] 2F71,[23] 2FJM,[110] 2FJN,[111]2H4G,[112] 2H4K,[112] 2HB1,[112] 2QBP,[113] 2QBQ,[113] 2QBR,[113] 2QBS,[113] 2VEU,[114] 2VEV,[114] 2VEW,[114] 2VEX,[114] 2VEY,[114] 2ZMM,[113] 2ZN7,[115] 3CWE,[116] 3I7Z,[117] 3I80,[117] 3ZMP,[118] 4BJO,[119] 4Y14,[120] 5K9W,[82] 5KA1,[82] 5T19,[121] 6B8E,[83] 6B8T,[83] 6B8X,[83] 6B90,[83] 7FQU,[85] 7FRR,[83] 7GTU,[86] 7GTV,[86] 7MM1,[122] 7RIN,[122] 8SKL,[123] 8XOY,[1]	Closed

[a] PTP1B structures that did not include crystallographic water molecules (PDB IDs: 1T4J,[73] 2HNP,[124] 2HNQ,[124] 1PTT[99] and 3ZMQ[113]) were omitted from analysis.

Table S2. Crystal structures of non-receptor PTPs[125] available in the Protein Data Bank,[1] and used for analysis in this work.

PDB ID	Gene	WPD-loop Conformation	Resolution (Å)	Ligand
2F71[82]	PTPN1	Closed	1.55	3-[3-(3(S)-methylcarbamoyl-7-sulfoamino-3,4-dihydro-1H-isoquinolin-2-yl)-3-oxo-propyl]-benzoic acid
8U1E[90]	PTPN1	Open	1.43	Unliganded
2QEP[22]	PTPN2	Closed	2.5	Unliganded
4S0G[126]	PTPN3	Closed	1.72	Unliganded
2B49[22]	PTPN4	Open	1.54	Unliganded
8SLS[28]	PTPN5	Open	1.71	SO_4
4GRZ[21]	PTPN6	Closed	1.37	PO_4
4HJP[127]	PTPN6	Open	1.4	Unliganded
1ZC0[26]	PTPN7	Closed	1.85	PO_4
3O4U[29]	PTPN7	Open	2.25	L-(+)-tartaric acid
6KZQ[128]	PTPN9	Closed	1.7	Unliganded
4GE6[24]	PTPN9	Open	1.4	N-(4-bromo-3-methylbenzoyl)-4-[difluoro(phosphono)methyl]-L-phenylalanyl-N~5~-(3-iodobenzoyl)-L-ornithyl-3-{[(4-hydroxy-3-methoxyphenyl)acetyl]amino}-D-alaninamide
3ZM1[129]	PTPN11	Open	1.4	Unliganded
5HDE[1]	PTPN12	Closed	1.62	PO_4
5J8R[130]	PTPN12	Open	2.04	Unliganded
1WCH[25]	PTPN13	Closed	1.85	PO_4

6IWD[131]	PTPN14	Open	1.8	Unliganded
4GFU[1]	PTPN18	Closed	2.0	Unliganded
2OC3[22]	PTPN18	Open	1.5	Unliganded
8GVV[27]	PTPN21	Closed	1.8	PO_4
8GWH[27]	PTPN21	Open	2.0	Unliganded
3BRH[1]	PTPN22	Closed	2.2	PO_4
3OLR[1]	PTPN22	Open	2.5	Unliganded

Table S3. Crystal structures of wild-type triosephosphate isomerase available in the Protein Data Bank,[1] and used for analysis in this work.[a]

PDB ID	Species	Resolution (Å)	Ligand
1M6J[132]	*Entamoeba histolytica*	1.50	Unliganded
1MO0[133]	*Caenorhabditis elegans*	1.70	Acetate
1R2R[134]	*Oryctolagus cuniculus*	1.50	Unliganded
1YPI[135]	*Saccharomyces cerevisiae*	1.90	Unliganded
2JK2[64]	*Homo sapiens*	1.70	Unliganded
3GVG[136]	*Mycobacterium tuberculosis*	1.55	Glycerol
3KRS[137]	*Cryptosporidium parvum Iowa II*	1.55	Unliganded
3KXQ[1]	*Bartonella henselae*	1.60	Nitrate
3M9Y[138]	*Staphylococcus aureus subsp. aureus MRSA252*	1.90	Citric Acid
4GNJ[1]	*Leishmania sp. 'siamensis*	1.93	Unliganded
4IOT[139]	*Escherichia coli str. K-12 substr. DH10B*	1.85	Unliganded
* 4MKN[140]	*Chlamydomonas reinhardtii*	1.1	Unliganded
4OBT[141]	*Arabidopsis thaliana*	1.6	Unliganded
* 4Y8F[142]	*Clostridium perfringens str. 13*	1.54	Unliganded
4Y96[142]	*Gemmata obscuriglobus*	1.58	Unliganded
5CSR[143]	*Thermoplasma acidophilum DSM 1728*	1.94	Unliganded

5TIM[144]	*Trypanosoma brucei*	1.83	Unliganded
5ZFX[145]	*Opisthorchis viverrini*	1.75	Unliganded
6R8H[146]	*Fasciola hepatica*	1.90	Unliganded
7N8U[1]	*Promethearchaeum syntrophicum*	1.98	Unliganded
7PEJ[1]	*Schizosaccharomyces pombe 972h-*	1.79	Acetate
* 7RCQ[1]	*Ktedonobacter racemifer*	1.70	Nitrate
* 7RPN[1]	*Bacteroides thetaiotaomicron*	1.37	Unliganded
7SKJ[1]	*Cuscuta australis*	1.90	Acetate (borderline, near the active site)

[a] PDB IDs marked with an * provide only the asymmetric unit and not the full biological assembly.

Supplementary References

(1) Berman, H. M.; Westbrook, J.; Feng, Z.; Gilliland, G.; Bhat, T. N.; Weissig, H.; Shindyalov, I. N.; Bourne, P. E. The Protein Data Bank. *Nucleic Acids Res.* **2000**, *28*, 235-242.

(2) Case, D. A.; Aktulga, H. M.; Belfon, K.; Cerutti, D. S.; Cisneros, G. A.; Cruzeiro, V. W. D.; Forouzesh, N.; Giese, T. J.; Götz, A. W.; Gohlke, H.; et al. AmberTools. *J. Chem. Inf. Model.* **2023**, *63*.

(3) Naden, L. N.; Nash, J.; Crawford, T. D.; McDonald, A. R. Cookiecutter for Computational Molecular Sciences: A Best Practices Ready Python Project Generator. *J. Chem. Ed.* **2024**, *101*, 5105-5109.

(4) Crean, R. M.; Biler, M.; van der Kamp, M. W.; Hengge, A. C.; Kamerlin, S. C. L. Loop Dynamics and Enzyme Catalysis in Protein Tyrosine Phosphatases. *J. Am. Chem. Soc.* **2021**, *143*, 3830-3845.

(5) van der Spoel, D.; Lindahl, E.; Hess, B.; Groenhof, G.; Mark, A. E.; Berendsen, H. J. C. GROMACS: Fast, Flexible and Free. *J. Comp. Chem.* **2005**, *26*, 1701-1718.

(6) Maier, J. A.; Martinez, C.; Kasavajhala, K.; Wickstrom, L.; Hauser, K. E.; Simmerling, C. ff14SB: Improving the Accuracy of Protein Side Chain and Backbone Parameters from FF99SB. *J. Chem. Theory. Comput.* **2015**, *11*, 3696-3713.

(7) Jorgensen, W. L.; Chandrasekhar, J.; Madura, J. D.; Impey, R. W.; Klein, M. L. Comparison of Simple Potential Functions for Simulating Liquid Water. *J. Chem. Phys.* **1983**, *79*, 926-935.

(8) Parrinello, M.; Rahman, A. Crystal Structure and Pair Potentials: A Molecular Dynamics Study. *Phys. Rev. Lett.* **1980**, *45*, 1196-1199.

(9) Parrinello, M.; Rahman, A. Polymorphic Transitions in Single Crystals: A New Molecular Dynamics Method. *J. Appl. Phys.* **1981**, *52*, 7182-7190.

(10) Ryckaert, J.-P.; Ciccotti, G.; Berendsen, H. J. C. Numerical Integration of the Cartesian Equations of Motion of a System with Constraints: Molecular Dynamics of *n*-Alkanes. *J. Comput. Phys.* **1977**, *23*, 327-341.

(11) Darden, T.; York, D.; Pedersen, L. Particle Mesh Ewald: An *N*·log(*N*) Method for Ewald Sums in Large Systems. *J. Chem. Phys.* **1993**, *98*, 10089-10092.

(12) Oakley, A. J.; Klvana, M.; Otyepka, M.; Nagata, Y.; Wilce, M. C.; Damborsky, J. Crystal Structure of Haloalkane Dehalogenase LinB from *Sphingomonas paucimobilis* UT26 at 0.95Å Resolution: Dynamics of Catalytic Residues. *Biochemistry* **2004**, *43*, 870-878.

(13) Brezovsky, J.; Babkova, P.; Degtjarik, O.; Fortova, A.; Gora, A.; Iemark, I.; Rezacova, P.; Dvorak, P.; Smatanova, I. K.; Prokop, Z.; et al. Engineering a De Novo Transport Tunnel. *ACS Catal.* **2016**, *6*, 7597-7610.

(14) Apweiler, R.; Bairoch, A.; Wu, C. H.; Barker, W. C.; Boeckmann, B.; Ferro, S.; Gasteiger, E.; Huang, H.; Lopez, R.; Magrane, M.; et al. UniProt: The Universital Protein Knowledgebase. *Nucleic Acids Res.* **2004**, *32*, D115-D119.

(15) McInnes, L.; Healy, J.; Astels, S. hdbscan: Hierarchical density based clustering. *J. Open Source Softw.* **2017**, *2*, 205.

(16) Mills, J. E. J.; Dean, P. M. Three-Dimensional Hydrogen-Bond Geometry and Probability Information from a Crystal Survey. *J. Comp.-Aid. Mol. Des.* **1996**, *10*, 607-622.

(17) Huš, M.; Urbic, T. Strength of Hydrogen Bonds of Water Depends on Local Environment. *J. Chem. Phys.* **2012**, *136*, 144305.

(18) Petukhov, M.; Rychkov, G.; Firsov, L.; Serrano, L. H-Bonding in Protein Hydration Revisited. *Prot. Sci.* **2009**, *13*, 2120-2129.

(19) Ippolito, J. A.; Alexander, R. S.; Christianson, D. W. Hydrogen Bond Stereochemistry in Protein Structure and Function. *J. Mol. Biol.* **1990**, *215*, 457-471.

(20) Gowers, R.; Linke, M.; Barnoud, J.; Reddy, T.; Melo, M.; Seyler, S.; Domanski, J.; Dotson, D.; Buchoux, S.; Kenney, I.; et al. MDAnalysis: A Python Package for the Rapid Analysis of Molecular Dynamics Simulations. *Proc. 15th Python Sci. Conf.* **2016**.

(21) Alicea-Velázquez, N. L.; Jakoncic, J.; Boggon, T. J. Structure-Guided Studies of the SHP-1/JAK1 Interaction Provide New Insights Into Phosphatase Catalytic Domain Substrate Recognition. *J. Struct. Biol.* **2013**, *181*, 243-251.

(22) Barr, A. J.; Ugochukwu, E.; Lee, W. H.; King, O. N.; Filippakopoulus, P.; Alfano, I.; Savitsky, P.; Burgess-Brown, N. A.; Muller, S.; Knapp, S. Large-Scale Structural Analysis of the Classical Human Protein Tyrosine Phosphatome. *Cell* **2009**, *136*, 352-363.

(23) Klopfenstein, S. R.; Evdokimov, A. G.; Colson, A.-O.; Fairweather, N. T.; Neuman, J. J.; Maier, M. B.; Gray, J. L.; Gerwe, G. S.; Stake, G. E.; Howard, B. W.; et al. 1,2,3,4-Tetrahydroisoquinolinyl Sulfamic Acids as Phosphatase PTP1B Inhibitors. *Bioorg. Med. Chem. Lett.* **2006**, *16*, 1574-1578.

(24) Zhang, S.; Liu, S.; Tao, R.; Wei, D.; Chen, L.; Shen, W.; Yu, Z. H.; Wang, L.; Jones, D. R.; Dong, X. C.; et al. A Highly Selective and Potent PTP-MEG2 Inhibitor with Therapeutic Potential for Type 2 Diabetes. *J. Am. Chem. Soc.* **2012**, *134*, 18116-18124.

(25) Villa, F.; Deak, M.; Bloomberg, G. B.; Alessi, D. R.; Van Aalten, D. M. F. Crystal Structure of Ptpl1/Fap-1 Human Tyrosine Phosphatase Mutated in Colorectal Cancer: Eveidence for a Second Phosphotyrosine Substrate Recognition Pocket. *J. Biol. Chem.* **2005**, *280*, 8180.

(26) Mustelin, T.; Tautz, L.; Page, R. Structure of the Hematopoietic Tyrosine Phosphatase (HePTP) Catalytic Domain: Structure of a KIM Phosphatase with Phosphate Bound at the Active Site. *J. Mol. Biol.* **2005**, *354*, 150-163.

(27) Chen, L.; Qian, Z.; Zheng, Y.; Zhang, J.; Sun, J.; Zhou, C.; Xiao, H. Structural Analysis of PTPN21 Reveals a Dominant-Negative Effect of the FERM Domain On Its Phosphatase Activity. *Sci. Adv.* **2024**, *10*, eadi7404-eadi7404.

(28) Guererro, L.; Ebrahim, A.; Riley, B. T.; Kim, M.; Huang, Q.; Finke, A. D.; Keedy, D. A. Pushed to Extremes: Distinct Effects of High Temperature vs. Pressure on the Structure of an Atypical Phosphatase. *Commun. Biol.* **2024**, *7*, 59.

(29) Critton, D. A.; Tautz, L.; Page, R. Visualizing Active-Site Dynamics in Single Crystals of HePTP: Opening of the WPD Loop Involves Coordinated Movement of the E Loop. *J. Mol. Biol.* **2011**, *405*, 619-629.

(30) Sheldrick, G. M. Phase Annealing in SHELX-90: Direct Methods for Larger Structures. *Acta Crystallogr. Sect. A. Found. Crystallogr.* **1990**, *46*, 467-473.

(31) Wlodawer, A.; Dauter, Z.; Rubach, P.; Minor, W.; Loch, J. I.; Brzezinski, D.; Gilski, M.; Jaskolski, M. Waterless Structures in the Protein Data Bank. *IUCrJ* **2024**, *11*, 966-976.

(32) S., J. J.; Brünger, A. T. Protein Hydration Observed by X-ray Diffraction. Solvation Properties of Penicillopepsin and Neuarminidase Crystal Structures. *J. Mol. Biol.* **1994**, *243*, 100-115.

(33) Nguyen, C. N.; Kurtzman Young, T.; Gilson, M. K. Grid Inhomogeneous Solvation Theory: Hydration Structure and Thermodynamics of the Miniature Receptor Cucurbit[7]Uril. *J. Chem. Phys.* **2012**, *137*, 044101.

(34) Patel, H.; Grüning, B. A.; Günther, S.; Merfort, I. PyWATER: A PyMOL Plug-In to Find Conserved Water Molecules in Proteins By Clustering. *Bioinformatics* **2014**, *30*, 2978-2980.

(35) López, E. D.; Arcon, J. P.; Gauto, D. F.; Petruk, A. A.; Modenutti, C. P.; Dumas, V. G.; Marti, M. A.; Turjanski, A. G. WATCLUST: A Tool for Improving the Design of Drugs Based on Protein-Water Interactions. *Bioinformatics* **2015**, *31*, 3697-3699.

(36) Magdziarz, T.; Mitusińska, K.; Gołdowska, S.; Płuciennik, A.; Stolarczyk, M.; Ługowska, M.; Góra, A. AQUA-DUCT: A Ligands Tracking Tool. *Bioinformatics* **2017**, *33*, 2045-2046.

(37) Haider, K.; Cruz, A.; Ramsey, S.; Gilson, M. K.; Kurtzman, T. Solvation Structure and Thermodynamic Mapping (SSTMap): An Open-Source, Flexible Package for the Analysis of Water in Molecular Dynamics Trajectories. *J. Chem. Theory Comput.* **2017**, *14*, 418-425.

(38) Fusani, L.; Wall, I.; Palmer, D.; Cortes, A. Optimal Water Networks in Protein Cativities with GAsol and 3D-RISM. *Bioinformatics* **2018**, *34*, 1947-1948.

(39) Cuzzolin, A.; Deganutti, G.; Salmaso, V.; Sturlese, M.; Moro, S. AquaMMapS: An Alternative Tool to Monitor the Role of Water Molecules During Protein-Ligand Association. *ChemMedChem* **2018**, *13*, 522-531.

(40) Nittinger, E.; Gibbons, P.; Eigenbrot, C.; Davies, D. R.; Maurer, B.; Yu, C. L.; Kiefer, J. R.; Kuglstatter, A.; Murray, J.; Ortwine, D. F.; et al. Water Molcules in Protein-Ligand Interfaces. Evaluation of Software Tools and SAR Comparison. *J. Comput. Aided Mol. Des.* **2019**, *33*, 307-330.

(41) Mitusińska, K.; Raczyńska, A.; Bzówka, M.; Bagrowska, W.; Góra, A. Applications of Water Molecules for Analysis of Macromolecule Properties. *Comp. Struct. Biotechnol. J.* **2020**, *18*, 355-365.

(42) Magdziarz, T.; Mitusińska, K.; Bzówka, M.; Raczyńska, A.; Stańczak, A.; Banas, M.; Bagrowska, W.; Góra, A. AQUA-DUCT 1.0: Structural and Functional Analysis of Macromolecules from an Intramolecular Voids Perspective. *Bioinformatics* **2020**, *36*, 2599-2601.

(43) Park, S.; Seok, C. GalaxyWater-CNN: Prediction of Water Positions on the Protein Structure by a 3D-Convolutional Neural Network. *J. Chem. Inf. Model.* **2022**, *62*, 3157-3168.

(44) Tošović, J.; Fijan, D.; Jukič, M.; Bren, U. Conserved Water Networks Identification for Drug Design Using Density Clustering Approaches on Positional and Orientational Data. *J. Chem. Inf. Mod.* **2022**, *62*, 6105-6117.

(45) Brezovsky, J.; Thirunavukarasu, A. S.; Surpeta, B.; Sequeiros-Borja, C. E.; Mandal, N.; Sarkar, D. K.; Foumthuim, C. J. D.; Agrawal, N. TransportTools: A Library for High-Throughput Analyses of Internal Voids in Biomolecules and Ligand Transport Through Them. *Bioinformatics* **2022**, *38*, 1752-1753.

(46) Kriegel, M.; Muller, Y. A. De Novo Prediction of Explicit Water Molecule Positions by a Novel Algorithm Within the Protein Design Software MUMBO. *Sci. Rep.* **2023**, 16680.

(47) Park, S. Water Position Prediction With SE(3)-Graph Neural Network. *bioRxiv* **2024**, DOI: 10.1101/2024.1103.1125.586555.

(48) Zamanos, A.; Ioannakis, G.; Emiris, I. Z. HydraProt: A New Deep Learning Tool for Fast and Accurate Prediction of Water Molecule Positions for Protein Structures. *J. Chem. Inf. Mod.* **2024**, *64*, 2594-2611.

(49) Krieger, J. M.; Doljanin, F.; Bogetti, A. T.; Zhang, F.; Manivarma, T.; Bahar, I.; Mikulska-Ruminska, K. WatFinder: A ProDy Tool for Protein–Water Interactions. *Bioinformatics* **2024**, *40*, btae516.

(50) Carugo, O.; Bordo, D. How Many Water Molecules Can Be Detected By Protein Crystallography? *Acta Crystallogr. D Biol. Crystallogr.* **1999**, *55*, 479-483.

(51) Jumper, J.; Evans, R.; Pritzel, A.; Green, T.; Figurnov, M.; Ronneberger, O.; Tunyasuvunakool, K.; Bates, R.; Žídek, A.; Potapenko, A.; et al. Highly Accurate Protein Structure Prediction With AlphaFold. *Nature* **2021**, *596*, 583-589.

(52) Wall, M. E.; Calabréo, G.; Bayly, C. I.; Mobley, D. L.; Warren, G. L. Biomolecular Solvation Structure Revealed by Molecular Dynamics Simulations. *J. Am. Chem. Soc.* **2019**, *141*, 4711-4720.

(53) Caldararu, O.; Ignjatović, M. M.; Oksanen, E.; Ryde, U. Water Structure in Solution and Crystal Molecular Dynamics Simulations Compared to Protein Crystal Structures. *RSC Adv.* **2020**, *10*, 8435-8443.

(54) Dahanayake, J. N.; Gautam, D. N.; Verma, R.; Mitchell-Koch, K. To Keep or Not to Keep? The Question of Crystallographic Waters for Enzyme Simulations in Organic Solvent. *Mol. Simul.* **2016**, *42*, 1001-1013.

(55) Anandakrishnan, R.; Izadi, S.; Onufriev, A. V. Why Computed Protein Folding Landscapes Are Sensitive to the Water Model. *J. Chem. Theory Comput.* **2018**, *15*, 625-636.

(56) Pathirannahalage, S. P. K.; Meftahi, N.; Elbourne, A.; Weiss, A. C. G.; McConville, C. F.; Padua, A.; Winkler, D. A.; Gomes, M. C.; Greaves, T. L.; Le, T. C.; et al. Systematic Comparison of the Structural and Dynamic Properties of Commonly Used Water Models for Molecular Dynamics Simulations. *J. Chem. Inf. Model.* **2021**, *61*, 4521-4536.

(57) Thirunavukarasu, A. S.; Szleper, K.; Tanriver, G.; Marchlewski, I.; Mitusinska, K.; Gora, A.; Brezovsky, J. Water Migration through Enzyme Tunnels Is Sensitive to the Choice of Explicit Water Model. *J. Chem. Inf. Model.* **2024**, *65*, 326-337.

(58) Sequeiros-Borja, C.; Surpeta, B.; Thirunavukarasu, A. S.; Foumthuim, C. J. D.; Marchlewski, I.; Brezovsky, J. Water will Find Its Way: Transport through Narrow Tunnels in Hydrolases. *J. Chem. Inf. Model.* **2024**, *64*, 6014-6025.

(59) Duran, C.; Casadevall, G.; Osuna, S. Harnessing Conformational Dynamics in Enzyme Catalysis to Achieve Nature-Like Catalytic Efficiencies: The Shortest Path Map Tool for Computational Enzyme Redesign. *Faraday Discuss.* **2025**, *252*, 306-322.

(60) Shen, R.; Brownless, A.-L.; Alansson, N.; Corbella, M.; Kamerlin, S. C. L.; Hengge, A. C. SHP-1 Variants Broaden the Understanding of pH-Dependent Activities in Protein Tyrosine Phosphatases. *JACS Au* **2024**, *4*, 2874-2885.

(61) Jogl, G.; Rozovsky, S.; McDermott, A. E.; Tong, L. Optimal Alignment for Enzymatic Proton Transfer: Structure of the Michaelis Complex of Triosephosphate Isomerase at 1.2-Å Resolution. *Proc. Natl. Acad. Sci. USA* **2003**, *100*, 50-55.

(62) Malabanan, M. M.; Amyes, T. L.; Richard, J. P. A Role for Flexible Loops in Catalysis. *Curr. Opin. Struct. Biol.* **2010**, *20*, 702-710.

(63) Liao, Q.; Kulkarni, Y.; Sengupta, U.; Petrovic, D.; Mulholland, A. J.; van der Kamp, M.; Strodel, B.; Kamerlin, S. C. L. Loop Motion in Triosephosphate Isomerase Is Not a Simple Open and Shut Case. *J. Am. Chem. Soc.* **2018**, *140*, 15889-15903.

(64) Rodríguez-Almazán, C.; Arreola, R.; Rodríguez-Larrea, D.; Aguirre-López, B.; Tuena de Gómez-Puyou, M.; Pérez-Montfrot, R.; Costas, M.; Gómez-Puyou, A.; Torres-Larios, A. Structural Basis of Human Triosephosphate Isomerase Deficiency: Mutation E104D is Related to Alterations of a Conserved Water Network at the Dimer Interface. *J. Biol. Chem.* **2008**, *283*, 23254-23263.

(65) Larsen, S. D.; Barf, T.; Liljebris, C.; May, P. D.; Ogg, D.; O'Sullivan, T. J.; Palazuk, B. J.; Schostarez, H. J.; Stevens, F. C.; Bleasdale, J. E. Synthesis and Biological Activity of a Novel Class of Small Molecular Weight Peptidomimetic Competitive Inhibitors of Protein Tyrosine Phosphatase 1B. *J. Med. Chem.* **2002**, *45*, 598-622.

(66) Szczepankiewicz, B. G.; Liu, G.; Hajduk, P. J.; Abad-Zapatero, C.; Pei, Z.; Xin, Z.; Lubben, T. H.; Trevillyan, J. M.; Stashko, M. A.; Ballaron, S. J.; et al. Discovery of a Potent, Selective Protein Tyrosine Phosphatase 1B Inhibitor Using a Linked-Fragment Strategy. *J. Am. Chem. Soc.* **2003**, *125*, 4087-4096.

(67) Xin, Z.; Oost, T. K.; Abad-Zapatero, C.; Hajduk, P. J.; Pei, Z.; Szczepankiewicz, B. G.; Hutchins, C. W.; Ballaron, S. J.; Stashko, M. A.; Lubben, T.; et al. Potent, Selective Inhibitors of Protein Tyrosine Phosphatase 1B. *Bioorg. Med. Chem. Lett.* **2003**, *13*, 1887-1890.

(68) Salmeen, A.; Andersen, J. N.; Myers, M. P.; Meng, T.-C.; Hinks, J. A.; Tonks, N. K.; Barford, D. Redox Regulation of Protein Tyrosine Phosphatase 1B Involves a Sulphenyl-Amide Intermediate. *Nature* **2003**, *423*, 769-773.

(69) van Montfort, R. L. M.; Congreve, M.; Tisi, D.; Carr, R.; Jhoti, H. Oxidation State of the Active-Site Cysteine in Protein Tyrosine Phosphatase 1B. *Nature* **2003**, *423*, 773-777.

(70) Lui, G.; Szczepankiewicz, B. G.; Pei, Z.; Janowick, D. A.; Xin, Z.; Hajduk, P. J.; Abad-Zapatero, C.; Liang, H.; Hutchins, C. W.; Fesik, S. W.; et al. Discovery and Structure-Activity Relationship of Oxalylarylaminobenzoic Acids as Inhibitors of Protein Tyrosine Phosphatase 1B. *J. Med. Chem.* **2003**, *46*, 2093-2103.

(71) Liu, G.; Xin, Z.; Liang, H.; Abad-Zapatero, C.; Hajduk, P. J.; Janowick, D. A.; Szczepankiewicz, B. G.; Pei, Z.; Hutchins, C. W.; Ballaron, S. J.; et al. Selective Protein Tyrosine Phosphatase 1B Inhibitors: Targeting the Second Phosphotyrosine Binding Site with Non-Carboxylic Acid-Containing Ligands. *J. Med. Chem.* **2003**, *46*, 3437-3440.

(72) Pei, Z.; Li, X.; Liu, G.; Abad-Zapatero, C.; Lubben, T.; Zhang, T.; Ballaron, S. J.; Hutchins, C. W.; Trevillyan, J. M.; Jirousek, M. R. Discovery and SAR of Novel, Potent and Selective Protein Tyrosine Phosphatase 1B Inhibitors. *Bioorg. Med. Chem. Lett.* **2003**, *13*, 3129-3132.

(73) Wiesmann, C.; Barr, K. J.; Kung, J.; Zhu, J.; Erlanson, D. A.; Shen, W.; Fahr, B. J.; Zhong, M.; Taylor, L.; Randal, M.; et al. Allosteric Inhibition of Protein Tyrosine Phosphatase 1B. *Nat. Struct. Mol. BIol.* **2004**, *11*, 730-737.

(74) Zhao, H.; Liu, G.; Xin, Z.; Serby, M. D.; Pei, Z.; Szczepankiewicz, B. G.; Hajduk, P. J.; Abad-Zapatero, C.; Hutchins, C. W.; Lubben, T. H.; et al. Isoxazole Carboxylic Acids as Protein Tyrosine Phosphatase 1B (PTP1B) Inhibitors. *Bioorg. Med. Chem. Lett.* **2004**, *14*, 5543-5546.

(75) Ala, P. J.; Gonneville, L.; Hillman, M. C.; Becker-Pasha, M.; Wei, M.; Reid, B. G.; Klabe, R.; Yue, E. W.; Wayland, B.; Douty, B.; et al. Structural Basis for Inhibition of Protein-Tyrosine Phosphatase 1B by Isothiazolidinone Heterocyclic Phosphonate Mimetics. *J. Biol. Chem.* **2006**, *281*, 32784.

(76) Abdo, M.; Liu, S.; Zhou, B.; Walls, C. D.; Wu, L.; Knapp, S.; Zhang, Z.-Y. Seleninate in Place of Phosphate: Irreversible Inhibition of Protein Tyrosine Phosphatases. *J. Am. Chem. Soc.* **2008**, *130*, 13196-13197.

(77) Liu, S.; Zeng, L. F.; Wu, L.; Yu, X.; Xue, T.; Gunawan, A. M.; Long, Y. Q.; Zhang, Z. Y. Targeting Inactive Enzyme Conformation: Aryl Diketoacid Derivatives as a New Class of PTP1B Inhibitors. *J. Am. Chem. Soc.* **2008**, *130*, 17075-17084.

(78) Chen, Y. Y.; Chu, H. M.; Pan, K. T.; Teng, C. H.; Wang, D. L.; Wang, A. H.; Khoo, K. H.; Meng, T. C. Cysteine S-Nitrosylation Protects Protein-tyrosine Phosphatase 1B against Oxidation-induced Permanent Inactivation. *J. Biol. Chem.* **2008**, *283*, 35265-35272.

(79) Zhou, H.; Singh, H.; Parsons, Z. D.; Lewis, S. M.; Bhattacharya, S.; Seiner, D. R.; LaButti, J. N.; Reilly, T. J.; Tanner, J. J.; Gates, K. S. The Biological Buffer Bicarbonate/CO_2 Potentiates H_2O_2-Mediated Inactivation of Protein Tyrosine Phosphatases. *J. Am. Chem. Soc.* **2011**, *133*, 15803-15805.

(80) Reddy, M. V.; Ghadiyaram, C.; Panigrahi, S. K.; Krishnamurthy, N. R.; Hosahalli, S.; Chandrasekharappa, A. P.; Manna, D.; Badiger, S. E.; Dubey, P. K.; Mangamoori, L. N. X-Ray Structure of PTP1B in Complex with a New PTP1B Inhibitor. *Protein Pept. Lett.* **2014**, *21*, 90-93.

(81) Xiao, P.; Wang, X.; Wang, H.-M.; Fu, X.-L.; Cui, F.; Yu, X.; Wen, S.; Bi, W.-X.; Sun, J.-P. The Second-Sphere Residue T263 Is Important for the Function and Catalytic Activity of PTP1B *via* Interaction with the WPD-Loop. *Int. J. Biochem. Cell BIol.* **2014**, *57*, 84-95.

(82) Choy, M. S.; Li, Y.; Machado, L. E. S. F.; Kunze, M. B. A.; Connors, C. R.; Wei, X.; Lindorff-Larsen, K.; Page, R.; Peti, W. Conformational Rigidity and Protein Dynamics at Distinct Timescales Regulate PTP1B Activity and Allostery. *Mol. Cell* **2017**, *65*, 644-658.e645.

(83) Keedy, D. A.; Hill, Z. B.; Biel, J. T.; Kang, E.; Rettenmaier, T. J.; Brandão-Neto, J.; Pearce, N. M.; von Delft, F.; Wells, J. A.; Fraser, J. S. An Expanded Allosteric Network in PTP1B by Multitemperature Crystallography, Fragment Screening, and Covalent Tethering. *eLife* **2018**, *7*, 1-36.

(84) Sarkar, A.; Kim, E. Y.; Jang, T.; Hongdusit, A.; Kim, H.; Choi, J. M.; Fox, J. M. Microbially Guided Discovery and Biosynthesis of Biologically Active Natural Products. *ACS Synth. biol.* **2021**, *10*, 1505-1519.

(85) Skaist Mehlman, T.; Biel, J. T.; Azeem, S. M.; Nelson, E. R.; Hossain, S.; Dunett, L.; Paterson, N. G.; Douangamath, A.; Talon, R.; Axford, D.; et al. Room-Temperature Crystallography Reveals Altered Binding of Small-Molecule Fragments to PTP1B. *eLife* **2023**, *12*, e84632.

(86) Mehlman, T. S.; Ginn, H. M.; Keedy, D. A. An Expanded View of Ligandability in the Allosteric Enzyme PTP1B From Computational Reanalysis of Large-Scale Crystallographic Data. *bioRxiv* **2024**, DOI: 10.1101/2024.1101.1105.574428.

(87) Chirgadze, Y. N.; Battaile, K. P.; Likhachev, I. V.; Balabaev, N. K.; Gordon, R. D.; Romanov, V.; Lin, A.; Karisch, R.; Lam, R.; Ruzanov, M.; et al. Signal Transfer in Human Protein Tyrosine Phosphatase PTP1B From Allosteric Inhibitor P00058. *J. Biomol. Struct. Dyn.* **2021**, *40*, 13823-13832.

(88) Sharma, S.; Ebrahim, A.; Keedy, D. A. Room-Temperature Serial Synchrotron Crystallography of the Human Phosphatase PTP1B. *Acta Crystallogr. F Struct. Biol. Commun.* **2023**, *79*, 23-30.

(89) Greisman, J. B.; Willmore, L.; Yeh, C. Y.; Giordanetto, F.; Shahamadtar, S.; Nisonoff, H.; Maragakis, P.; Shaw, D. E. Discovery and Validation of the Binding Poses of Allosteric Fragment Hits to Protein Tyrosine Phosphatase 1B: From Molecular Dynamics Simulations to X-ray Crystallography. *J. Chem. Inf. Model.* **2023**, *63*, 2644-2650.

(90) Sharma, S.; Skaist Mehlman, T.; Sagabala, R. S.; Boivin, B.; Keedy, D. A. High-Resolution Double Vision of the Allosteric Phosphatase PTP1B. *Acta Crystallogr. F Struct. Biol. Commun.* **2024**, *80*, 1-12.

(91) Perdikari, A.; Woods, V. A.; Ebrahim, A.; Lawler, K.; Bounds, R.; Singh, N. I.; Mehlman, T. S.; Riley, B. T.; Sharma, S.; Morris, J. W.; et al. Structures of Human PTP1B Variants Reveal Allosteric Sites to Target for Weight Loss Therapy. *bioRxiv* **2025**, DOI: 10.1101/2024.1108.1105.603709.

(92) Puius, Y. A.; Zhao, Y.; Sullivan, M.; Lawrence, D. S.; Almo, S. C.; Zhang, Z. Y. Identification of a Second Aryl Phosphate-Binding Site in Protein-Tyrosine Phosphatase 1B: A Paradigm for Inhibitor Design. *Proc. Natl. Acad. Sci. USA* **1997**, *94*, 13420-13425.

(93) Groves, M. R.; Yao, Z. J.; Roller, P. P.; Burke Jr., T. R.; Barford, D. Structural Basis for Inhibition of the Protein Tyrosine Phosphatase 1B by Phosphotyrosine Peptide Mimetics. *Biochemistry* **1998**, *37*, 17773-17783.

(94) Sarmiento, M.; Puius, Y. A.; Vetter, S. W.; Keng, Y. F.; Wu, L.; Zhao, Y.; Lawrence, D. S.; Almo, S. C.; Zhang, Z. Y. Structural Basis of Plasticity in Protein Tyrosine Phosphatase 1B Substrate Recognition. *Biochemistry* **2000**, *39*, 8171-8179.

(95) Bleasdale, J. E.; Ogg, D.; Palazuk, B. J.; Jacobs, C. S.; Swanson, M. L.; Wany, X. Y.; Thompson, D. P.; Conradi, R. A.; Mathews, W. R.; Laborde, A. L.; et al. Small Molecule Peptidomimetics Containing a Novel Phosphotyrosine Bioisostere Inhibit Protein Tyrosine Phosphatase 1B and Augment Insulin Action. *Biochemistry* **2001**, *40*, 5642-5654.

(96) Jia, Z.; Ye, Q.; Dinaut, A. N.; Wang, Q.; Waddleton, D.; Payette, P.; Ramachandran, C.; Kennedy, B.; Hum, G.; Taylor, S. D. Structure of Protein Tyrosine Phosphatase 1B in Complex with Inhibitors Bearing Two Phosphotyrosine Mimics. *J. Med. Chem.* **2001**, *44*, 4584-4594.

(97) Jia, Z.; Ye, Q.; Dinaut, A. N.; Wang, Q.; Waddleton, D.; Payette, P.; Ramachandran, C.; Kennedy, B.; Hum, G.; Taylor, S. D. Structure of Protein Tyrosine Phosphatase 1B in Complex with Inhibitors Bearing Two Phosphotyrosine Mimics. *J. Med. Chem.* **2001**, *44*, 4584-4594.

(98) Asante-Appiah, E.; Patel, S.; Dufresne, C.; Roy, P.; Wang, Q.; Patel, V.; Friesen, R. W.; Ramachandran, C.; Becker, J. W.; Leblanc, Y.; et al. The Structure of PTP1B in Complex With a Peptide Inhibitor Reveals an Alternative Binding Mode for Bisphosphonates. *Biochemistry* **2002**, *41*, 9043-9051.

(99) Jia, Z.; Barford, D.; Flint, A. J.; Tonks, N. K. Structural Basis for Phosphotyrosine Peptide Recognition by Protein Tyrosine Phosphatase 1B. *Science* **1995**, *268*, 1754-1758.

(100) Sun, J.-P.; Fedorov, A. A.; Lee, S.-Y.; Guo, X.-L.; Shen, K.; Lawrence, D. S.; Almo, S. C.; Zhang, Z.-Y. Crystal Structure of PTP1B Complexed with a Potent and Selective Bidentate Inhibitor. *J. Biol. Chem.* **2003**, *278*, 12406-12414.

(101) Liu, G.; Xin, Z.; Pei, Z.; Hajduk, P. J.; Abad-Zapatero, C.; Hutchins, C. W.; Zhao, H.; Lubben, T. H.; Ballaron, S. J.; Haasch, D. L.; et al. Fragment Screening and Assembly: A Highly Efficient Approach to a Selective and Cell Active Protein Tyrosine Phosphatase 1B Inhibitor. *J. Med. Chem.* **2003**, *46*, 4232-4235.

(102) Scapin, G.; Patel, S. B.; Becker, J. W.; Wang, Q.; Desponts, C.; Waddleton, D.; Skorey, K.; Cromlish, W.; Bayly, C.; Therien, M.; et al. The Structural Basis for the Selectivity of Benzotriazole Inhibitors of PTP1B. *Biochemistry* **2003**, *42*, 11451-11459.

(103) Xin, Z.; Liu, G.; Abad-Zapatero, C.; Pei, Z.; Szczepankiewicz, B. G.; Li, X.; Zhang, T.; Hutchins, C. W.; Hajduk, P. J.; Ballaron, S. J.; et al. Identification of a Monoacid-Based, Cell Permeable, Selective Inhibitor of Protein Tyrosine Phosphatase 1B. *Bioorg. Med. Chem. Lett.* **2003**, *13*, 3947-3950.

(104) Pedersen, A. K.; Peters, G. H.; Møller, K. B.; Iversen, L. F.; Kastrup, J. S. Water-Molecule Network and Active-Site Flexibility of Apo Protein Tyrosine Phosphatase 1B. *Acta Crystallogr. D Biol. Crystallogr.* **2004**, *60*, 1527-1534.

(105) Hartshorn, M. J.; Murray, C. W.; Cleasby, A.; Frederickson, M.; Tickle, I. J.; Jhoti, H. Fragment-Based Lead Discovery Using X-Ray Crystallography. *J. Med. Chem.* **2005**, *48*, 403.

(106) Moretto, A. F.; Kirincich, S. J.; Xu, W. X.; Smith, M. J.; Wan, Z.-K.; Wilson, D. P.; Follows, B. C.; Binnun, E.; Joseph-McCarthy, D.; Foreman, K.; et al. Bicyclic and Tricyclic Thiophenes as Protein Tyrosine Phosphatase 1B Inhibitors. *Bioorg. Med. Chem.* **2006**, *14*, 2162-2177.

(107) Peters, G. H.; Iversen, L. F.; Branner, S.; Andersen, H. S.; Mortensen, S. B.; Olsen, O. H.; Moller, K. B.; Moller, N. P. Residue 259 Is a Key Determinant of Substrate Specificity of Protein-Tyrosine Phosphatases 1B and Alpha. *J. Biol. Chem.* **2000**, *275*, 18201-18209.

(108) Black, E.; Breed, J.; Breeze, A. L.; Embrey, K.; Garcia, R.; Gero, T. W.; Godfrey, L.; Kenny, P. W.; Morley, A. D.; Minshull, C. A.; et al. Structure-Based Design of Protein Tyrosine Phosphatase-1B Inhibitors. *Bioorg. Med. Chem. Lett.* **2005**, *15*, 2503-2507.

(109) Ala, P. J.; Gonneville, L.; Hillman, M.; Becker-Paha, M.; Yue, E. W.; Douty, B.; Wayland, B.; Polam, P.; Crawley, M. L.; McLaughlin, E.; et al. Structural Insights Into the Design of Nonpeptidic Isothiazolidinone-Containing Inhibitors of Protein-Tyrosine Phosphatase 1B. *J. Biol. Chem.* **2006**, *281*, 38013.

(110) Asante-Appiah, E.; Patel, S.; Desponts, C.; Taylor, J. M.; Lau, C.; Dufresne, C.; Therien, M.; Friesen, R.; Becker, J. W.; LeBlanc, Y.; et al. Conformation-Assisted Inhibition of Protein-Tyrosine Phosphatase-1B Elicits Inhibitor Selectivity Over T-Cell Protein-Tyrosine Phosphatase. *J. Biol. Chem.* **2006**, *281*, 8010-8015.

(111) Asante-Appiah, E.; Patel, S.; Desponts, C.; Taylor, J. M.; Lau, C.; Dufresne, C.; Therien, M.; Friesen, R.; Becker, J. W.; Leblanc, Y.; et al. Conformation-Assisted Inhibition of Protein-Tyrosine Phosphatase-1B Elicits Inhibitor Selectivity over T- Cell Protein-Tyrosine Phosphatase. *J. Biol. Chem.* **2006**, *281*, 8010-8015.

(112) Wan, Z.-K.; Lee, J.; Xu, W.; Erbe, D. V.; Joseph-McCarthy, D.; Follows, B. C.; Zhang, Y.-L. Monocyclic Thiophenes as Protein Tyrosine Phosphatase 1B Inhibitors: Capturing Interactions with Asp48. *Bioorg. Med. Chem. Lett.* **2006**, *16*, 4941-4945.

(113) Wilson, D. P.; Wan, Z.-K.; Xu, W.-X.; Kirincich, S. J.; Follows, B. C.; Joseph-McCarthy, D.; Foreman, K.; Moretto, A.; Wu, J.; Zhu, M.; et al. Structure-Based Optimization of Protein Tyrosine

Phosphatase 1B Inhibitors: From the Active Site to the Second Phosphotyrosine Binding Site. *J. Med. Chem.* **2007**, *50*, 4681-4698.

(114) Douty, B.; Wayland, B.; Ala, P. J.; Bower, M. J.; Pruitt, J.; Bostrom, L.; Wei, M.; Klabe, R.; Gonneville, L.; Wynn, R.; et al. Isothiazolidinone Inhibitors of PTP1B Containing Imidazoles and Imidazolines. *J. Med. Chem. Lett.* **2008**, *18*, 66-71.

(115) Wan, Z.-K.; Lee, J.; Hotchandani, R.; Moretto, A.; Binnun, E.; Wilson, D. P.; Kirincich, S. J.; Follows, B. C.; Ipek, M.; Xu, W.; et al. Structure-Based Optimization of Protein Tyrosine Phosphatase-1 B Inhibitors: Capturing Interactions with Arginine 24. *ChemMedChem* **2008**, *3*, 1525-1529.

(116) Han, Y.; Belley, M.; Bayly, C. I.; Colucci, J.; Dufresne, C.; Giroux, A.; Lau, C. K.; Leblanc, Y.; McKay, D.; Therien, M.; et al. Discovery of [(3-bromo-7-cyano-2-naphthyl)(difluoro)methyl]phosphonic acid, a Potent and Orally Active Small Molecule PTP1B Inhibitor. *Bioorg. Med. Chem. Lett.* **2008**, *18*, 3200-3205.

(117) Brandão, T. A. S.; Hengge, A. C.; Johnson, S. J. Insights into the Reaction of Protein-Tyrosine Phosphatase 1B. *J. Biol. Chem.* **2010**, *285*, 15874-15883.

(118) Meyer, C.; Hoeger, B.; Temmerman, K.; Tatarek-Nossol, M.; Pogenberg, V.; Bernhagen, J.; Wilmanns, M.; Kapurniotu, A.; Kohn, M. Development of Accessibile Peptidic Tool Compounds to Study the Phosphatase PTP1B in Intact Cells. *ACS Chem. Biol.* **2014**, *9*, 769.

(119) Kenny, P. W.; Newman, J.; Peat, T. S. Nitrate in the Active Site of Protein Tyrosine Phosphatase 1B Is a Putative Mimetic of the Transition State. *Acta Crystallogr. Sect. D Biol. Crystallogr.* **2014**, *70*, 565-571.

(120) Krishnan, N.; Krishnan, K.; Connors, C. R.; Choy, M. S.; Page, R.; Peti, W.; Van Aelst, L.; Shea, S. D.; Tonks, N. K. PTP1B Inhibition Suggests a Therapeutic Strategy for Rett Syndrome. *J. Clin. Invest.* **2015**, *125*, 3163-3177.

(121) Punthasee, P.; Laciak, A. R.; Cummings, A. H.; Ruddraraju, K. V.; Lewis, S. M.; Hillebrand, R.; Singh, H.; Tanner, J. J.; Gates, K. S. Covalent Allosteric Inactivation of Protein Tyrosine Phosphatase 1B (PTP1B) by an Inhibitor-Electrophile Conjugate. *Biochemistry* **2017**, *56*, 2051-2060.

(122) Greisman, J. B.; Dalton, K. M.; Sheehan, C. J.; Klureza, M. A.; Kurinov, I.; Hekstra, D. R. Native SAD Phasing at Room Temperature. *Acta Crystallogr. D. Struct. Biol.* **2002**, *78*, 986-996.

(123) Liang, S.; Tran, E.; Du, X.; Dong, J.; Sudholz, H.; Che, H.; Qu, Z.; Huntington, N. D.; Babon, J. J.; Kershaw, N. J.; et al. A Small Molecule Inhibitor of PTP1B and PTPN2 Enhances T Cell Anti-tumor Immunity. *Nat. Commun.* **2023**, *14*, 4524.

(124) Barford, D.; Flint, A. J.; Tonks, N. K. Crystal Structure of Human Protein Tyrosine Phosphatase 1B. *Science* **1994**, *263*, 1397-1404.

(125) Alonso, A.; Pulido, R. The Extended Human PTPome: A Growing Tyrosine Phosphatase Family. *FEBS J.* **2016**, *283*, 2197-2201.

(126) Chen, K. E.; Li, M. Y.; Chou, C. C.; Ho, M. R.; Chen, G. C.; Meng, T. C.; Wang, A. H. Substrate Specificity and Plasticity of FERM-Containing Protein Tyrosine Phosphatases. *Structure* **2015**, *23*, 653-664.

(127) Alicea-Velazquez, N. L.; Boggon, T. J. SHP Family Protein Tyrosine Phosphatases Adopt Canonical Active-Site Conformations in the Apo and Phosphate-Bond States. *Protein Pept. Lett.* **2013**, *20*, 1039-1048.

(128) Xu, Y. F.; Chen, X.; Yang, Z.; Xiao, P.; Liu, C. H.; Li, K. S.; Yang, X. Z.; Wang, Y. J.; Zhu, Z. L.; Xu, Z. G.; et al. PTP-MEG2 Regulates Quantal Size and Fusion Pore Opening Through Two Distinct Structural Bases and Substrates. *EMBO Rep.* **2021**, *22*, e52141.

(129) Grosskopf, S.; Eckert, C.; Arkona, C.; Radetzki, S.; Böhm, K.; Heinemann, U.; Wolber, G.; von Kries, J.-P.; Birchmeier, W.; Rademann, J. Selective Inhibitors of the Protein Tyrosine Phosphatase SHP2 Block Cellular Motility and Growth of Cancer Cells in vitro and in vivo. *ChemMedChem* **2015**, *10*, 815-826.

(130) Li, H.; Yang, F.; Liu, C.; Xiao, P.; Xu, Y. F.; Liang, Z. L.; Liu, C.; Wang, H. M.; Wang, W. J.; Zheng, W. S.; et al. Crystal Structure and Substrate Specificty of PTPN12. *Cell Rep.* **2016**, *15*, 1-14.

(131) Yun, H. Y.; Kim, M. W.; Lee, H. S.; Kim, W.; Shin, J. H.; Kim, H.; Shin, H. C.; Park, H.; Oh, B. H.; Kim, W. K.; et al. Structural Basis for Recognition of the Tumor Suppressor Protein PTPN14 by the Oncoprotein E7 of Human Papillomavirus. *PLoS Biol.* **2019**, *17*, e3000367-e3000367.

(132) Rodríguez-Romero, A.; Hernández-Santoyo, A.; del Pozo Yauner, L.; Kornhauser, A.; Fernández-Velasco, D. A. Structure and Inactivation of Triosephosphate Isomerase from *Entamoeba histolytica*. *J. Mol. Biol.* **2002**, *322*, 669-675.

(133) Symersky, J.; Li, S.; Carson, M.; Luo, M. Structural Genomics of *Caenorhabditis elegans*: Triosephosphate isomerase. *Prot. Struct. Func. Bioinformat.* **2003**, *51*, 484-486.

(134) Aparicio, R.; Ferreia, S. T.; Polikarpov, I. Closed Conformation of the Active Site Loop of Rabbit Muscle Triosephosphate Isomerase in the Absence of Substrate: Evidence of Conformational Heterogeneity. *J. Mol. Biol.* **2003**, *334*, 1023-1041.

(135) Lolis, E.; Alber, T.; Davenport, R. C.; Rose, D.; Hartman, F. C.; Petsko, G. A. Structure of Yeast Triosephosphate Isomerase at 1.9-.ANG. Resolution. *Biochemistry* **1990**, *29*, 6609-6618.

(136) Baugh, L.; Phan, I.; Begley, D. W.; Clifton, M. C.; Armour, B.; Dranow, D. M.; Taylor, B. M.; Muruthi, M. M.; Abendroth, J.; Fairman, J. W.; et al. Increaing the Structural Coverage of Tuberculosis Drug Targets. *95* **2015**, 142-148.

(137) Nguyen, T. N.; Abendroth, J.; Leibly, D. J.; Le, K. P.; Guo, W.; Kelley, A.; Stewart, L.; Myler, P. J.; Van Voorhis, W. C. Structure of Triosephosphate Isomerase from *Cryptosporidium parvum. Acta Cryst. F Struct. Biol. Commun.* **2011**, *67*, 1095-1099.

(138) Mukherjee, S.; Roychowdhury, A.; Dutta, D.; Kumar Das, A. Crystal Structures of Triosephosphate Isomerase from Methicillin Resistant *Staphylococcus auerus* MRSA252 Provide Structural Insights into Novel Modes of Ligand Binding and Unique Conformations of Catalytic Loop. *Biochimie* **2012**, *94*, 2532-2544.

(139) Kozlov, G.; Vinaik, R.; Gehring, K. Triosephosphate Isomerase is a Common Crystallization Contaminant of Soluble His-Tagged Proteins Produced in *Escherichia coli. Acta Cryst. Struct. Biol. Commun.* **2013**, *F69*, 499-502.

(140) Zaffagnini, M.; Michelet, L.; Sciabolini, C.; Di Giacinto, N.; Morisse, S.; Marchand, C. H.; Trost, P.; Fermani, S.; Lemaire, S. D. High-Resolution Crystal Structure and Redox Properties of Chloroplastic Triosephosphate Isomerase from *Chlamydomonas reinhardtii*. *Mol. Plant* **2014**, *7*, P101-120.

(141) López-Castillo, L.; Jiménez-Sandoval, P.; Baruch-Torres, N.; Trasviña-Arenas, C. H.; Díaz-Quezada, C.; Lara-González, S.; Winkler, R.; Brieba, L. G. Structural Basis for Redox Regulation of Cytoplasmic and Chloroplastic Triosephosphate Isomerases from *Arabidopsis thaliana*. *Front. Plant Sci.* **2016**, *7*, 1817.

(142) Romero-Romero, S.; Costas, M.; Rodríguez-Romero, A.; Fernández-Velasco, D. A. Reversibility and Two State Behaviour in the Thermal Unfolding of Oligomeric TIM Barrel Proteins. *Phys. Chem. Chem. Phys.* **2015**, *17*, 20699-20714.

(143) Park, S. H.; Kim, H. S.; Park, M. S.; Moon, S.; Song, M. K.; Park, H. S.; Hahn, H.; Kim, S.-J.; Bae, E.; Kim, H.-J.; et al. Structure and Stability of the Dimeric Triosephosphate Isomerase from the Thermophilic Archaeon *Thermoplasma acidophilum*. *PLoS One* **2015**, *10*, e0145331.

(144) Hegazy, R.; Richard, J. P. Triosephosphate Isomerase: The Crippling Effect of the P168A/I172A Substitution at the Heart of an Enzyme Active Site. *Biochemistry* **2023**, *62*, 2916-2927.

(145) Son, J.; Kim, S.; Kim, S. E.; Lee, H.; Lee, M.-R.; Hwang, K. Y. Structural Analysis of an Epitope Candidate of Triosephosphate Isomerase in *Opisthorchis viverrini*. *Sci. Rep.* **2018**, *8*, 15075.

(146) Ferraro, F.; Corvo, I.; Bergalli, L.; Ilarraz, A.; Cabrera, M.; Gil, J.; Susuki, B. M.; Caffrey, C. R.; Timson, D. J.; Robert, X.; et al. Novel and Selective Inactivators of Triosephosphate Isomerase With Anti-Trematode Activity. *Sci. Rep.* **2020**, *10*, 2587.
